# Multiple Osmotic Stress Responses in *Acidihalobacter prosperus* Result in Tolerance to Chloride Ions

**DOI:** 10.3389/fmicb.2016.02132

**Published:** 2017-01-05

**Authors:** Mark Dopson, David S. Holmes, Marcelo Lazcano, Timothy J. McCredden, Christopher G. Bryan, Kieran T. Mulroney, Robert Steuart, Connie Jackaman, Elizabeth L. J. Watkin

**Affiliations:** ^1^Centre for Ecology and Evolution in Microbial Model Systems, Linnaeus UniversityKalmar, Sweden; ^2^Facultad de Ciencias Biologicas, Universidad Andres BelloSantiago, Chile; ^3^Center for Bioinformatics and Genome Biology, Fundacion Ciencia y VidaSantiago, Chile; ^4^School of Biomedical Sciences, Curtin Health Innovation Research Institute, Curtin UniversityPerth, WA, Australia

**Keywords:** salt, acidophile, biomining, bioleaching, proteomics, pyrite, chalcopyrite, environmental stress

## Abstract

Extremely acidophilic microorganisms (pH optima for growth of ≤3) are utilized for the extraction of metals from sulfide minerals in the industrial biotechnology of “biomining.” A long term goal for biomining has been development of microbial consortia able to withstand increased chloride concentrations for use in regions where freshwater is scarce. However, when challenged by elevated salt, acidophiles experience both osmotic stress and an acidification of the cytoplasm due to a collapse of the inside positive membrane potential, leading to an influx of protons. In this study, we tested the ability of the halotolerant acidophile *Acidihalobacter prosperus* to grow and catalyze sulfide mineral dissolution in elevated concentrations of salt and identified chloride tolerance mechanisms in *Ac. prosperus* as well as the chloride susceptible species, *Acidithiobacillus ferrooxidans*. *Ac. prosperus* had optimum iron oxidation at 20 g L^−1^ NaCl while *At. ferrooxidans* iron oxidation was inhibited in the presence of 6 g L^−1^ NaCl. The tolerance to chloride in *Ac. prosperus* was consistent with electron microscopy, determination of cell viability, and bioleaching capability. The *Ac. prosperus* proteomic response to elevated chloride concentrations included the production of osmotic stress regulators that potentially induced production of the compatible solute, ectoine uptake protein, and increased iron oxidation resulting in heightened electron flow to drive proton export by the F_0_F_1_ ATPase. In contrast, *At. ferrooxidans* responded to low levels of Cl^−^ with a generalized stress response, decreased iron oxidation, and an increase in central carbon metabolism. One potential adaptation to high chloride in the *Ac. prosperus* Rus protein involved in ferrous iron oxidation was an increase in the negativity of the surface potential of Rus Form I (and Form II) that could help explain how it can be active under elevated chloride concentrations. These data have been used to create a model of chloride tolerance in the salt tolerant and susceptible species *Ac. prosperus* and *At. ferrooxidans*, respectively.

## Introduction

Acidophilic and extremely acidophilic microorganisms have pH optima for growth of ≤5 and ≤3, respectively, and comprise a phylogenetically and phenotypically diverse group of microorganisms from all three domains of life (reviewed in Aguilera et al., 2016; Dopson, 2016; Golyshina et al., 2016). As a group, they comprise species across wide temperature ranges for growth (from eurypsychrophilic to thermophiles), with the ability to exploit organic and/or inorganic electron donors and carbon sources, and can utilize molecular oxygen, ferric iron, and sulfate as electron acceptors. Acidophilic microorganisms have generated considerable interest as: (i) they catalyze the dissolution of sulfide minerals for recovery of valuable metals, termed “biomining” or “bioleaching” (Vera et al., 2013); (ii) they can cause uncontrolled sulfide mineral oxidation leading to the release of toxic, acidic and metal containing waters, called “acid mine drainage” (Mendez-Garcia et al., 2015); (iii) they are a source of extremozymes for use in biotechnologies (Elleuche et al., 2014); (iv) liposomes from these species have been investigated as a method for drug delivery (Jensen et al., 2015); and (v) these microorganisms may be analogs for early life on earth and potential life on other planets (Bauermeister et al., 2014).

*Acidithiobacillus ferrooxidans* was the first microorganism recognized to generate acid mine drainage (Colmer and Hinkle, 1947) and has since been identified in many acidic environments playing an important role during heap bioleaching of sulfide minerals. *At. ferrooxidans* fixes carbon dioxide for cellular carbon and couples ferrous iron, inorganic sulfur compound, and hydrogen oxidation to the reduction of either molecular oxygen or ferric iron. The type strain genome sequence is available (Valdes et al., 2008) and the genetic basis of many aspects of its metabolism has been elucidated (Osorio et al., 2008, 2013; Quatrini et al., 2009; Esparza et al., 2010; Ponce et al., 2012). *Acidihalobacter prosperus* (originally described as “*Thiobacillus prosperus*”) is another autotrophic and acidophilic species capable of growth via oxidation of ferrous iron and inorganic sulfur compounds (Huber and Stetter, 1989; Cardenas et al., 2015). The *Ac. prosperus* type strain was isolated from a volcanic marine environment and is halotolerant, being able to grow in chloride concentrations from 0.04 to 0.6 M (2.3–35 g L^−1^; Nicolle et al., 2009). The underlying mechanisms for *Ac. prosperus* growth are far less well-understood than for *At. ferrooxidans*, although the recent publication of its genome sequence (Ossandon et al., 2014) now aids investigation of this species.

Acidophiles employ a number of methods to maintain their intracellular pH near to neutral despite a proton ion gradient of up to 10,000 fold across the cytoplasmic membrane (reviewed in Slonczewski et al., 2009; Zammit and Watkin, 2016). These mechanisms include: (i) primary proton pumps that export protons during electron transport; (ii) cytoplasmic membranes that are highly resistant to the influx of protons; (iii) an inside positive membrane potential that creates a chemiosmotic gradient that reduces proton influx by electrostatic repulsion; (iv) alterations in cytoplasmic membrane structure; (v) proton-consuming reactions such as carboxylases; and (vi) cytoplasmic buffering. Osmotic stress occurs when the soluble extracellular salts differ from the concentration within the cell that either leads to cellular dehydration or lysis (Zammit and Watkin, 2016). Typical acidophile biomining strains are highly sensitive to anions and in particular chloride that have been demonstrated to inhibit ferrous iron oxidation by a *Leptospirillum ferriphilum*-dominated culture (Gahan et al., 2010) and the bioleaching ability of an undefined mixed acidophile consortium (Shiers et al., 2005). One exception is the salt tolerant industrial isolate, *L. ferriphilum* Sp-Cl and its genome sequence will aid in discovering adaptations to high salt concentrations in acidophiles (Issotta et al., 2016). The greater sensitivity to the membrane permeable anion chloride is due to its ability to cross the cell membrane. This reduces the transmembrane potential resulting in an influx of protons and acidification of the cytoplasm (Suzuki et al., 1999). Other anions such as ${\text{SO}}_{4}^{2-}$, and cations such as Na^+^, have little effect beyond their impact on osmotic potential (Blight and Ralph, 2004; Shiers et al., 2005; Davis-Belmar et al., 2008; Rea et al., 2015; Boxall et al., 2016).

The isolation and investigation of halotolerant/halophilic acidophiles has long been a goal due to their potential use for biomining in countries where freshwater is limited and the use of seawater would be beneficial (Zammit et al., 2012; Rea et al., 2015). The major constituents of standard seawater are; Cl^−^ (0.56M) and Na^+^ (0.48M) with ${\text{SO}}_{4}^{2-}$ at much lower concentrations (0.03M; Millero et al., 2008). Given the sensitivity of acidophiles to Cl^−^, those to be utilized in biomining with seawater must be able to tolerate the dual stresses of low pH and high Cl^−^ concentrations. Adaptations to high salt concentrations exhibited by halophilic/halotolerant microorganisms include: (i) accumulation of cytoplasmic potassium; production of osmo-protectants in the cytoplasm to maintain an even turgor pressure inside and outside of the cell; (ii) alterations in the cell membrane, and (iii) an increase in acidic amino acids on the surface of proteins resulting in an elevated negative potential that aids in keeping the protein in solution (Shivanand and Mugeraya, 2011; Oren, 2013; Graziano and Merlino, 2014). In addition, changes in the surface electrostatic potential of a halophilic/halotolerant electron transport proteins is likely to affect their interactions with redox partners as has been shown for the interaction of the blue copper protein amicyanin with methylamine dehydrogenase (Ma et al., 2007; Choi et al., 2011). The combined effect of low pH and an anion such as chloride is to collapse the inside positive membrane potential involved in pH homeostasis (Alexander et al., 1987; Suzuki et al., 1999). However, the mechanisms halo-acidophiles utilize to combat these combined stresses are poorly understood and the majority of the studies to date have focused on species susceptible to increased salt while halotolerant acidophiles have been neglected.

Acidophilic bacteria have proven to be recalcitrant to the development of genetic methods, such as the creation of knockout mutants, and such techniques are only recently becoming more common (Wen et al., 2014; Yu et al., 2014). As a result, many acidophile studies have utilized “omics” techniques, including proteomics to investigate not only whole communities (Belnap et al., 2011; Mueller et al., 2011; Goltsman et al., 2013) but also specific responses in a single species (Baker-Austin et al., 2010; Mykytczuk et al., 2011; Potrykus et al., 2011; Mangold et al., 2013). In this study, iron oxidation and biomining studies along with a proteomic strategy are used to investigate the differing responses to chloride by the salt susceptible and tolerant acidophiles *At. ferrooxidans* and *Ac. prosperus*, respectively.

## Materials and methods

### Strains and growth conditions

*At. ferrooxidans* ATCC 23270^T^ and *Ac. prosperus* DSM 5130^T^ were obtained from Deutsche Sammlung von Mikroorganismen und Zellkulturen GmbH (DSMZ) and grown under the following conditions. *At. ferrooxidans*^T^ was cultured in pH 1.8 basal salts medium (BSM) (Plumb et al., 2002) and *Ac. prosperus*^T^ in DSMZ media 477 with 12.5 g/L NaCl (pH 2.5). Filter sterilized (0.2 μm Minisart, Sartorius Stedim) substrates (50 mM FeSO_4_·7 H_2_O and 5 mM K_2_S_4_O_6_) were added to both media. Cultures were incubated on a rotary shaker at 120 rpm at 30°C. The effect of NaCl on the iron oxidizing activity of *At. ferrooxidans*^T^ and *Ac. prosperus*^T^ was investigated by addition of NaCl to the media to achieve the required Cl^−^ concentrations. Cells were counted using a Helber bacteria counting chamber (Hawksley) at 400-fold magnification. Iron(II) oxidation was determined in *At. ferrooxidans*^T^ using spectrophotometry (Govender et al., 2012) and in *Ac. prosperus*^T^ experiments by titration against CeSO_4_ (Dopson and Lindström, 1999).

### Electron microscopy

*At. ferrooxidans*^T^ and *Ac. prosperus*^T^ cells (80 mL) were removed from log phase planktonic grown cultures, filtered (8.0 μm pore size nitrocellulose filters; Miltex™) to remove iron hydroxysulfate precipitate, and concentrated by centrifugation for 20 min at 48,000 × g at 4°C. Cell pellets were washed and resuspended with growth media and then 5.0 × 10^6^ cells were collected by centrifugation for 20 min at 48,000 × g at 4°C and resuspended in 100 μL of the appropriate growth media. Of this concentrated culture, 30 μL was pipetted onto an A1 SEM aluminum stub and incubated at 37°C for 10–40 min (until the surface appeared barely dry). Twenty Five microliters of 2.5% gluteraldehyde in BSM pH 2.5 was pipetted onto the surface of the stubs and then incubated at 4°C for 3 h. The sample stub was then washed by gently pipetting Invitrogen Gibco Ultrapure Distilled Water over the surface. Samples were dehydrated by sequential 30 min incubations with 70, 90, and 100% ethanol at 37°C before being transferred to a desiccator for 24 h. Stubs were coated with a 5 nm layer of platinum and imaged using a Zeiss Neon 40ESB Crossbeam Electron Microscope. Cell debris was differentiated from inorganic precipitates using SEM-EDX Spectra.

### Determination of cell viability by flow cytometry

A single dye viability assay was developed for cells with an internal positive membrane potential. SYTO 9 is natively fluorescent although the fluorescence increases by a factor of ten when bound to DNA (Ankarcrona et al., 1995; Knowles et al., 1996). SYTO9 will cross the membrane of cells with an inside negative membrane potential via passive diffusion. However, the inside positive membrane potential of live acidophiles will exclude SYTO9. Cells of non-viable acidophiles will lose their membrane potential and SYTO 9 will be able to cross the membrane by passive diffusion, binding to the DNA, and fluorescing brighter. The difference in fluorescence intensity between live and dead cells is 10-fold (Supplemental File 1). The cell viability assay was carried out by first removing ferric iron precipitates from cultures by filtration using 8.0 μm Miltex™ nitrocellulose filters followed by centrifugation at 700 × g for 1 min at 4°C. Cells were harvested from the supernatant by centrifugation at 48,000 × g for 20 min at 4°C and re-suspended in either BSM, pH 1.8 (*At. ferrooxidans*^T^) or DSMZ 477 media, pH 2.5 (*Ac. prosperus*^T^). Cell suspensions were adjusted to a density of 5.0 × 10^5^ cells/mL. SYTO 9 (ThermoFisher, Eugene, OR) was added to a concentration of 5 μM and the samples were incubated, protected from light, for 15 min. Three controls were prepared: (i) “no stain,” a 1 mL aliquot of cells was incubated at 4°C until time of acquisition with no further handling; (ii) “untreated,” a 1 mL aliquot of cells was incubated at 4°C for 15 min prior to acquisition, at which time it was stained with 5 μM SYTO 9, and (iii) “heat treated,” a 1 mL aliquot was heated to 60°C for 120 min and then incubated in 80% vol/vol molecular biology grade ethanol at room temperature for 60 min and then stained with 5 μM SYTO 9. To confirm the non-viability of this sample, a 200 μL aliquot was inoculated into 800 μL of appropriate growth media with 50 mM FeSO_4_.7 H_2_O and 5 mM K_2_S_4_O_6_ and incubated for 48 h at 30°C.

Samples were acquired in technical triplicates on an Attune Acoustic Flow Cytometer (ThermoFisher) using Attune Cytometric software version 1.2.5. on high sensitivity mode at a flow rate of 25 μL/min. Acquisition was terminated once 10,000 events on all gates were collected or 3 min had elapsed. Photomultiplier tube (PMT) voltages for forward scatter and side scatter were adjusted such that the bacterial population was clearly visible, with no truncation of relevant populations. PMT voltages were set on an unstained aliquot of cells with mean fluorescence intensity (MFI) of ~10^2^ arbitrary fluorescence units excited at 488 nm with a 515–545 nm filter. Gating strategies and the determination of the fluorescence properties of populations of interest were established using FlowJo v10.0 (FlowJo LLC) and an unpaired *t*-test between conditions was performed using the GraphPad Prism v6 Software Suite (Graphpad Software, Inc.).

### Bioleaching of sulfide minerals

Milled concentrates (<0.75 μm) of pyrite (FeS_2_), chalcopyrite (CuFeS_2_), and pentlandite [(Ni,Fe)_9_S_8_] were sterilized by gamma irradiation (50 kGray). The elemental compositions of the concentrates were determined using inductively coupled plasma—atom emission spectroscopy (ICP-AES) after borax flux and re-dissolution in 5% (vol/vol) HNO_3_. The pyrite concentrate contained (wt/wt) 36.6% Fe, 0.24% Cu, 0.04% Ni, and 39.8% S; the chalcopyrite contained 26.6% Fe, 26.8% Cu, and 29.8% S; and the pentlandite contained 40.7% Fe, 0.73% Cu, 7.01% Ni, and 35.4% S. *Ac. prosperus*^T^ was incubated in 100 mL of DSM 144 media containing increasing concentrations of NaCl and 0.5% (wt/vol) of the respective sulfide mineral concentrates. The cultures were incubated at 30°C with shaking at 130 rpm and evaporation was compensated for by the addition of sterile deionized water acidified to pH 2.5 with sulfuric acid. Leachates were assayed for pH and ORP (vs. Ag/AgCl) using Ionode pH (IJ44A) and ORP (IJ64) electrodes connected to a TPS SmartCHEM pH reader; Fe(III) concentration using the method of Govender et al. (2012); and Cu and Ni concentrations using flame atomic absorption spectrophotometry (Avanta Σ) with standards supplied by FLUKA chemicals.

### Proteomic analysis of growth at high or low chloride concentrations

Two liter cultures of the isolates were grown as described above with low and high concentrations of NaCl (0 and 8 g/L for *At. ferrooxidans*^T^ and 3.5 and 30 g/L for *Ac. prosperus*^T^). To avoid alterations within the proteome as a result of differences in the growth phase, cultures were harvested by centrifugation for 20 min at 48,000 × g and 4°C during mid-exponential phase as determined by Fe^3+^ concentrations. Cell pellets were washed in acidified ultrapure water (HpH_2_O, pH 1.8 or 2.5), re-pelleted by centrifugation for 20 min at 48,000 × g and 4°C, and stored at −80°C.

The *At. ferrooxidans*^T^ total soluble proteome was analyzed by 2D-PAGE as described by Mangold et al. (2011) except the initially extracted proteins were concentrated through methanol precipitation and 400 μg of protein was added to each IPG strip. Two dimensional gels were run in duplicate for cells grown in high salt and four gels were run for cells grown at low salt. Images of gels were taken on a PerkinElmer ProXPRESS and analyzed using Progenesis Same Spots program (Non-Linear Dynamics, USA). The two stained gels, of proteins isolated from cells grown in high NaCl concentrations, were aligned to the four stained gels, of proteins isolated from cells grown in low NaCl concentrations. Protein spots that showed change in abundance >1.8-fold and *p* < 0.05 were included in the identification process. Protein spots were analyzed at the Proteomics Node of the Lotterywest State Biomedical Facility within the Western Australian Institute for Medical Research. Protein samples were trypsin digested and analyzed by tandem mass spectrometry using a 5800 Proteomics Analyser (AB Sciex, USA) and peptides identified using Mascot sequence matching software with Ludwig NR Database and taxonomy set to bacteria.

Differential expression of the *Ac. prosperus*^T^ proteome was analyzed by isobaric tags for relative and absolute quantification (iTRAQ). The cell pellet was suspended in lysis buffer [0.2% vol/vol IGEPAL, 0.2% vol/vol Triton X, 0.2% wt/vol CHAPS, 75 mM NaCl, 1 mM EDTA, protease inhibitors; in phosphate buffered saline (PBS)] and sonicated using Misonix Ultrasonic Liquid Processor S-4000 (Sonica LLC) with the following parameters; Amplitude 30% and 5 s cycles (pulse on and off) for a total of 2 min. Cellular debris was removed by centrifugation at 13,000 × g for 10 min at 4°C and the supernatant stored at −80°C. iTraq analysis was performed by Proteomics International as follows: protein samples were acetone diafiltrated, reduced, alkylated, and trypsin digested according to the iTRAQ protocol (Applied Biosystems) and labeled using the iTRAQ reagents. Peptides were desalted on a Strata-X 33 μm polymeric reversed phase column (Phenomenex) and dissolved in a buffer containing 10 mM KH_2_PO_4_ pH 3 in 10% vol/vol acetonitrile before separation by strong cation exchange liquid chromatography (SCX) on an Agilent 1100 High Performance Liquid Chromatography system using a PolySulfoethyl column (4.6 × 100 mm; 5 μm; 300 A). Peptides were eluted with a linear gradient of 0 to 400 mM KCl. Eight fractions containing the peptides were collected and after desalting on Strata-X columns were loaded onto a Agilent Zorbax 300SB-C18, 3.5 μm column (Agilent Technologies) running on an Shimadzu Prominence nano HPLC system (Shimadzu). Peptides were resolved with a gradient of 10–40% vol/vol acetonitrile (0.1% vol/vol trifluoroacetic acid) over 160 min. The resultant spots were analyzed on a 5600 Triple Time of Flight mass spectrometer (AB Sciex). Spectral data were analyzed against a protein sequence database for the whole genome (Ossandon et al., 2014) using ProteinPilot™ 4.5 Software (AB Sciex).

### Rusticyanin discovery, multiple alignments, and model building

The amino acid sequence of the rusticyanin protein of *At. ferrooxidans*^T^ (locus tag: AFE_3146) was used as a query in a BlastP search (Altschul et al., 1997) to predict similar proteins (genes) in the genome of *Ac. prosperus*^T^ (Ossandon et al., 2014). Predicted protein sequences were aligned with Clustal Omega (Sievers et al., 2011) using the server at http://www.ebi.ac.uk/Tools/msa/clustalo/. Predictions of peptide signal and subcellular location were carried out using SignalP 4.1 (Petersen et al., 2011) and Cello (Yu et al., 2006) using the servers at http://www.cbs.dtu.dk/services/SignalP/ and http://cello.life.nctu.edu.tw/, respectively.

Three dimensional models of the structures of Rus Forms I and II from *Ac. prosperus*^T^ were constructed using the experimentally determined structure of rusticyanin from *At. ferrooxidans*^T^ (PDB “1RCY”) as a template (Walter et al., 1996). Electrostatic potentials of all three rusticyanins were determined using SWISS MODEL and visualized in Swiss-PDBViewer using the Swiss Model server at https://swissmodel.expasy.org/ (Bordoli et al., 2008). Default parameters were used [dielectric constant (solvent) 80.000, using only charged residues] using the Coulomb computational method with a dielectric constant (protein) 1.000 and solvent ionic strength (mol/L) 0.0.

## Results and discussion

### Iron oxidation by *At. ferrooxidans* and *Ac. prosperus* in the presence of chloride

**Figure 1:**
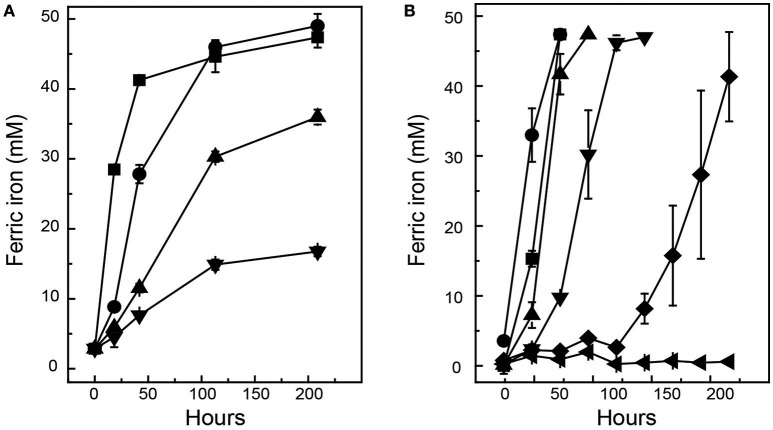
**Effect of NaCl upon of ferric iron generation during growth of ***At. ferrooxidans***^**T**^ (A)** and *Ac. prosperus*^T^ **(B)**. Symbols for *At. ferrooxidans*^T^: ■, 0 g L^−1^; ●, 6 g L^−1^; ▲, 8 g L^−1^; and ▼, 10 g L^−1^ while the symbols for *Ac. prosperus*^T^ are: ■, 3.8 g L^−1^; ●, 20 g L^−1^; ▲, 35 g L^−1^; ▼, 50 g L^−1^; ♦, 60 g L^−1^; and ◄, 75 g L^−1^. Data are averages ± *SD* of duplicate biological replicates and one to three technical replicates.

**Figure 2:**
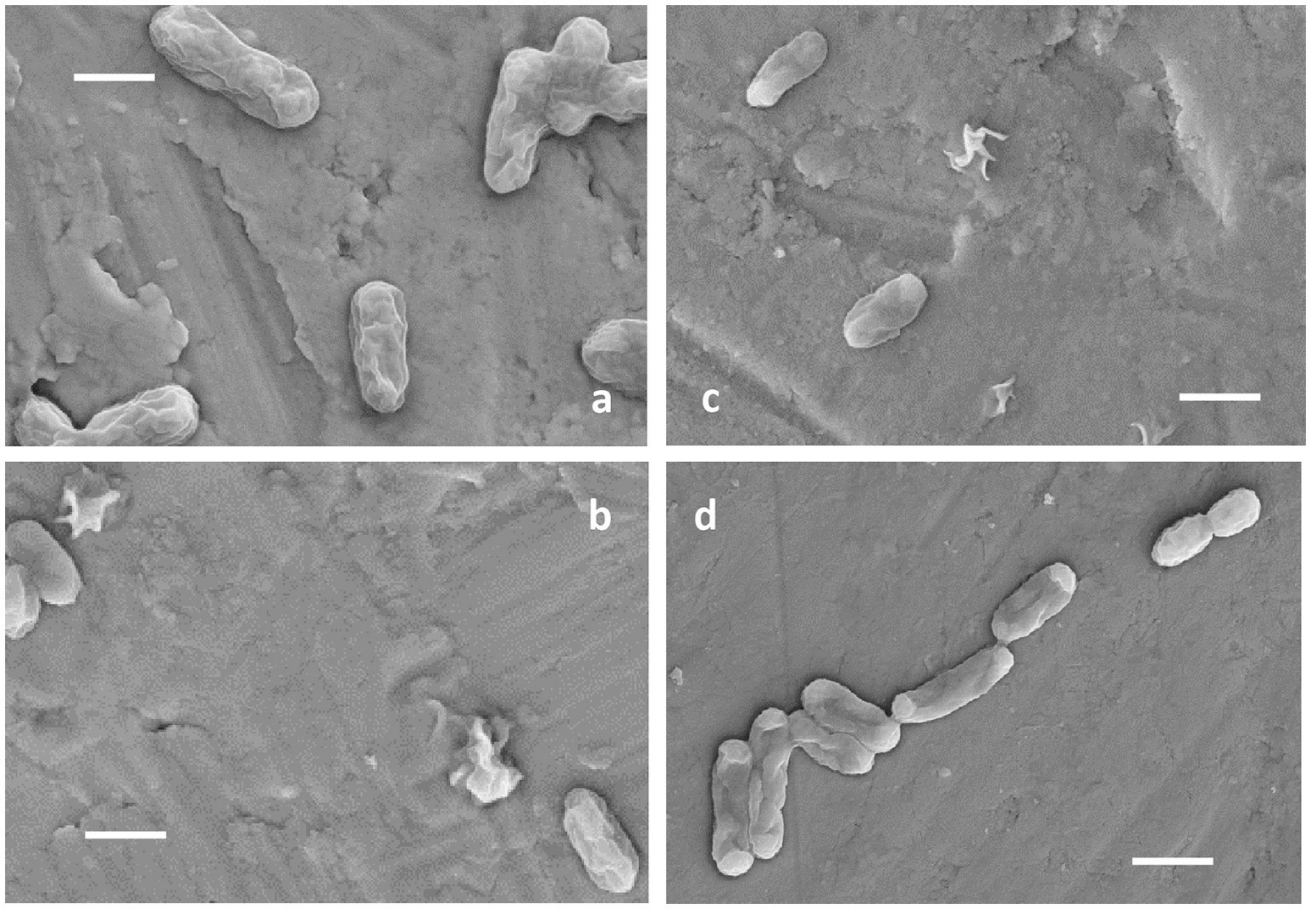
**Electron micrographs of ***At. ferrooxidans***^**T**^ grown in the presence of 0 g L^**−1**^ (A)** and 3.5 g L^−1^ NaCl **(B)** and *Ac. prosperus*^T^ in the presence of 12.5 g L^−1^ **(C)**, and 30 g L^−1^ NaCl **(D)**. All scale bars are 1 μm.

*Ac. prosperus*^T^ maintained activity (as defined by iron oxidation) at a higher concentration of NaCl compared to *At. ferrooxidans*^T^ (Figure 1). *At. ferrooxidans*^T^ ferrous iron oxidation was ~25% inhibited in the presence of 8 g L^−1^ NaCl and ~65% inhibited with the addition of 10 g L^−1^ NaCl (Figure 1A). In contrast, *Ac. prosperus*^T^ had the highest rate of ferric iron generation in the presence of 20 g L^−1^ NaCl. In addition, while *Ac. prosperus*^T^ ferrous iron oxidation in the presence of 50 g L^−1^ NaCl was less rapid, the ferrous iron was completely oxidized within 96 h (Figure 1B). Scanning electron micrographs indicate that *At. ferrooxidans*^T^ was healthier at 0 g L^−1^ NaCl compared to 3.5 g L^−1^ NaCl with many of the *At. ferrooxidans*^T^ cells lysed at the higher concentration. The lysed cells were confirmed as organic in nature by SEM-EDX Spectra (data not shown). In comparison *Ac. prosperus*^T^ cells appeared more healthy in the presence of 30 g L^−1^ NaCl compared with 12.5 g L^−1^ NaCl (Figure 2). An optimum NaCl concentration of 20 g L^−1^ (342 mM) suggests *Ac. prosperus*^T^ is a “slight halophile” (Ollivier et al., 1994).

A single dye viability assay using SYTO9 was developed based on positively charged SYTO 9 being excluded by live cells with an inside positive membrane potential. The cell viability of *At. ferrooxidans*^T^ grown at 3.5 g L^−1^ NaCl decreased by 50% (*P* < 0.01) compared to 0 g L^−1^ NaCl whereas *Ac. prosperus*^T^ had a 30% increase (*P* < 0.001) in viable cells when grown at 30 g L^−1^ NaCl compared to 12.5 g L^−1^ NaCl (Supplemental File 1).

### *Ac. prosperus* catalyzed sulfide mineral bioleaching in the presence of chloride

**Figure 3:**
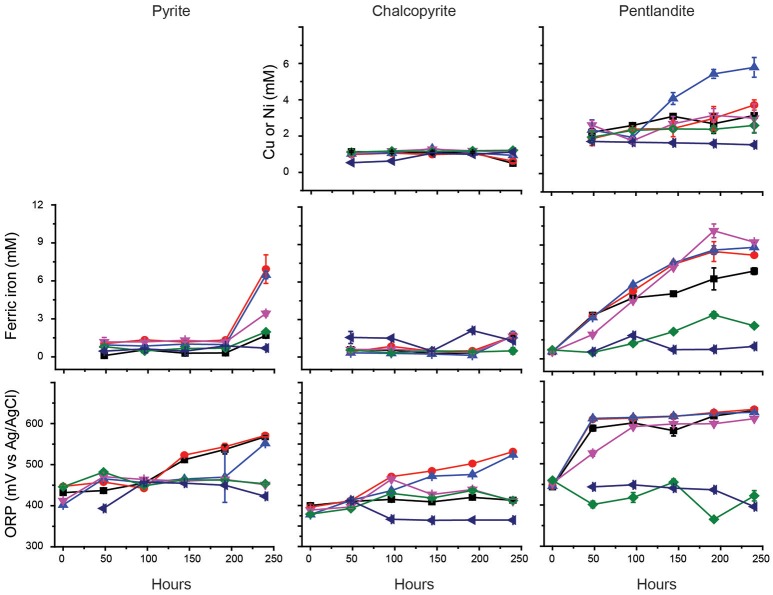
**Effect of NaCl on the bioleaching of pyrite, chalcopyrite, and pentlandite by ***Ac. prosperus***^**T**^: ■, 3.8 g L^**−1**^; ●, 15 g L^**−1**^; ▲, 30 g L^**−1**^; ▼, 50 g L^**−1**^; ♦, 75 g L^**−1**^; and ◄ abiotic control in the presence of 15 g L^**−1**^ NaCl (chosen as closest to the optimal NaCl concentration for ***Ac. prosperus***^**T**^ growth)**. The figure shows the copper and nickel concentrations during chalcopyrite and pentlandite leaching, respectively. Data are averages ± *SD* of triplicate biological replicates and three technical replicates.

Previous studies have indicated that the ability of *At. ferrooxidans*^T^ to leach metal sulfides in the presence of chloride is completely inhibited in the presence of ~3.5 g/L NaCl (Deveci, 2002; Deveci et al., 2008; Zammit et al., 2012; Bevilaqua et al., 2013). Due to the potential use of halo-acidophiles to carry out biomining in arid environments where saline groundwater is used (Zammit et al., 2012; Rea et al., 2015), the ability of *Ac. prosperus*^T^ to catalyze metal release from sulfide mineral concentrates was evaluated (Figure 3). The generation of ferric iron during *Ac. prosperus*^T^ catalyzed pyrite bioleaching was more rapid in the presence of 15 and 30 g L^−1^ NaCl compared to either 3.8 or 50 g L^−1^ NaCl. The pyrite bioleaching in the presence of NaCl confirmed that *Ac. prosperus*^T^ is a slight halophile. Ferric iron generation from pentlandite was similar in the presence of 15, 30, and 50 g L^−1^ NaCl and more rapid than observed at 3.5 and 75 g L^−1^ NaCl. Nickel release was greatest at 30 g L^−1^ NaCl (Figure 3). However, this trend was not supported for ferric iron generation and copper release from chalcopyrite where the leaching rates were very low and there was no statistically significant difference between 3.5 and 75 g L^−1^ NaCl. This lack of difference in leaching rates was potentially due to the advantages of chalcopyrite bioleaching in chloride systems as opposed to sulfate systems (reviewed in Watling, 2014). However, not all studies find an advantage with higher chloride ions at temperatures below 50°C (Dutrizac and Macdonald, 1971).

### *Ac. prosperus* proteomic response to the presence of chloride

Differential expression of the total soluble proteome from *Ac. prosperus*^T^ cultures grown in the presence of 3.5 and 30 g L^−1^ NaCl identified 617 proteins in each of the proteomes of which 125 were differentially expressed (*P* < 0.05; Table 1 plus the complete data set in Supplemental File 2). The COG classifications with the highest number of differentially expressed proteins were cell envelope integrity, protein synthesis, energy acquisition, central carbon metabolism, and protein fate (Supplemental File 3). This likely reflected the need to adjust the cell envelope to maintain cellular integrity and the increased energy required to maintain pH and osmotic balance (reviewed in Slonczewski et al., 2009; Zammit and Watkin, 2016).

**Table 1:** **Up- and down-regulated ***Ac. prosperus***^**T**^ proteins in the presence of high (30 g L^**−1**^) and low (3.5 g L^**−1**^) concentrations of sodium chloride**.

**Accession^a^**		**Fold^b^**	***SE*^c^**
**UP-REGULATED IN HIGH SALT CONDITIONS**			
**Osmoregulation, Cell Envelope, and Its Integrity**			
WP_038086993	Osmolarity response regulator, OmpR	Unique^d^	NA^e^
WP_038089003	UDP-N-acetylglucosamine 1-carboxyvinyltransferase, murA	Unique	NA
WP_038093711	ADP-L-glycero-D-mannoheptose-6-epimerase, RfaD	Unique	NA
WP_038088939	D-alanine–D-alanine ligase, DdL	Unique	NA
WP_038089461	Ectoine ABC transporter solute-binding protein	55.3	1.6
WP_052064239	MlaC, ABC transporter	10.5	1.8
WP_052064215	Peptidoglycan-associated lipoprotein OmpA	8.4	3.3
WP_038091948	Cytoskeleton protein RodZ	7.6	1.1
WP_038087491	ATP-dependent zinc metalloprotease, FtsH	6.3	2.3
WP_038089009	Outer membrane lipid asymmetry maintenance protein MlaD	5.8	2.6
WP_070079811	Pilus Assembly Protein PilG	5.7	1.2
WP_038093779	BtuB, Outer membrane cobalamin receptor protein	5.6	0.8
WP_065089743	Gram-negative porin	5.6	0.2
WP_052064070	Probable peptidyl-prolyl cis-trans isomerase, SurA	4.8	1.0
WP_038088090	Preprotein translocase subunit SecB	4.6	0.8
WP_065089354	Chaperone SurA	4.5	0.7
WP_038086391	3-ketoacyl-(Acyl-carrier-protein) reductase	3.9	0.4
WP_065089408	Tol-pal system protein YbgF	3.7	0.3
WP_065089387	Translocation protein TolB	2.2	0.3
**Stress Response**			
WP_065089122	Chaperone protein HscA	Unique	NA
WP_038091761	50S ribosomal protein L25/general stress protein Ctc	21.0	11.1
WP_038092421	10 kDa chaperonin, GroES	15.7	3.7
WP_038092418	60 kDa chaperonin, GroEL	9.2	3.7
WP_038092694	AhpC/TSA family	7.9	1.4
WP_038086510	ADP-ribose pyrophosphatase, NudF	7.4	2.5
WP_038086634	RNA polymerase-binding transcription factor, DksA	7.0	2.0
WP_065089055	Molecular chaperone, DnaK	7.0	1.1
WP_065089054	Heat shock protein, GrpE	4.3	0.8
OBS10750	ATP-dependent Clp protease	4.3	0.9
WP_038086793	Ribosome recycling factor	3.9	0.5
WP_038089562	Rubrerythrin protein	3.3	1.5
WP_065089340	Dyp-type peroxidase family	1.1	0.2
**Metabolism and Energy Conservation**			
OBS09221	Cytochrome *c*_1_ family	Unique	NA
WP_065089467	Rusticyanin protein	9.7	2.3
WP_038087805	ATP synthase subunit b	8.2	3.5
WP_038092630	SirA-like protein	6.6	3.3
WP_038089319	50S ribosomal protein L29	3.8	0.4
WP_038088471	Sulfur oxidation protein, SoxZ	2.9	0.3
**UP-REGULATED IN LOW SALT CONDITIONS**			
**Osmoregulation**			
WP_052064171	OmpA	0.5	0.3
WP_038092666	Protein AsmA	0.3	0.1
**Cell Envelope and Its Integrity**			
OBS10484	UDP-glucose pyrophosphorylase, GalU	0.1	0.0
**Metabolism and Energy Conservation**			
WP_038093510	Ribulose bisphosphate carboxylase large chain	0.7	0.3
WP_065089545	ATP synthase subunit alpha	0.7	0.2
WP_038089302	50S ribosomal protein L23, RplW	0.5	0.3
WP_038093481	Glyceraldehyde-3-phosphate dehydrogenase	0.4	0.0
WP_038091971	Enolase	0.4	0.1
WP_065089725	SoxAX cytochrome complex subunit A	0.4	0.2
WP_038089305	50S ribosomal protein L2, RplB	0.2	0.0
WP_038094109	50S ribosomal protein L10	0.2	0.0
OBS10998	Translation initiation factor IF-3	0.2	0.1
WP_038089313	30S ribosomal protein S3, RpsC	0.1	0.0
WP_038093513	Ribulose bisphosphate carboxylase small chain	0.1	0.0
WP_038089345	30S ribosomal protein S13, RpsM	0.1	0.0
WP_070077256	Major carboxysome shell protein 1A	0.1	0.0
WP_038093488	Fructose-1,6-bisphosphate aldolase	0.1	0.0

a *Accession numbers refers to the identified protein within the non-redundant protein sequence database for Acidihalobacter prosperus*.

b *Average fold up-regulation of the four independent pairwise comparisons between the duplicate high and low salt proteomes*.

c *Standard error of the mean of the average fold up-regulation for the four independent comparisons between treatments*.

d *Unique protein not expressed in low salt conditions*.

e *NA, not available as the protein was unique*.

Growth in high salt compared to low salt resulted in the unique identification of the osmolarity response regulator, OmpR. This regulator senses alterations in the membrane surface tension as a result of changes in the medium osmolarity (Cai and Inouye, 2002). A further regulatory protein, PilG which acts to control the transcription of many genes including osmotically inducible (Bouvier et al., 1998) and osmotic control (Lucht et al., 1994) genes, was 5.7 ± 1.2 fold up-regulated. On exposure to osmotic stress microorganisms will accumulate compatible solutes of which the most common is ectoine (Empadinhas and da Costa, 2008) and its ABC transporter was 55.3 ± 1.6 fold up-regulated in high salt conditions.

Another known response to osmotic stress is the production of proteins involved in the maintenance of the cell membrane integrity (reviewed: Zammit and Watkin, 2016). Growth in high salt conditions resulted in up-regulation of many *Ac. prosperus*^T^ membrane integrity proteins. These included cytoskeleton protein RodZ (7.6 ± 1.1 fold) that is linked to maintaining cell shape (Bendezu et al., 2009); the cell membrane integrity Tol-Pal system (Lloubes et al., 2001; Zakharov et al., 2004) proteins BtuB (5.6 ± 0.8 fold), SecB (4.6 ± 0.8 fold), YbgF (3.7 ± 0.3 fold), and TolB (2.2 ± 0.3 fold); the MlaD outer membrane lipid asymmetry maintenance protein (5.8 ± 2.6 fold) and MlaC phospholipid ABC transporter (10.5 ± 1.8 fold) that maintain outer membrane integrity (Malinverni and Silhavy, 2009); a Gram-negative porin (5.6 ± 0.2 fold) and SurA (4.8 ± 1.0 and 4.5 ± 0.7 fold) involved in outer membrane protein folding (Vertommen et al., 2009). Additionally, several proteins that form the cell membrane had higher levels of abundance including MurA (unique in high salt conditions); RfaD and DdL (both unique) involved in lipopolysaccharide and peptidoglycan biosynthesis, respectively; and a peptidoglycan-associated lipoprotein (8.4 ± 3.3 fold). An increase in membrane biosynthesis proteins in the presence of chloride has also been observed in *Acidithiobacillus caldus* (Guo et al., 2014).

A second group of *Ac. prosperus*^T^ proteins with increased concentrations in response to high salt conditions were related to the stress response. These proteins included protein folding chaperones such as DnaK (7.0 ± 1.1 fold) and GrpE (4.3 ± 0.8 fold) that form a homolog of the eukaryotic Hsp70 chaperone (Mayer and Bukau, 2005); HscA (unique) that forms part of a chaperone similar to DnaK (Silberg et al., 1998); and GroL (9.2 ± 3.7 fold) that acts under stress conditions (Chuang and Blattner, 1993). A further group of up-regulated proteins were involved in oxidative stress and included a AhpC/TSA family protein (7.9 ± 1.4 fold), ruberythrin (3.3 ± 1.5 fold), and a Dyp-type peroxidase family protein (1.1 ± 0.2 fold). These proteins may have been produced due to the increased metabolic and electron transport rate (see below) necessary to remove protons from the cytoplasm. This response has previously been observed in the acidophiles *At. caldus* (Zammit et al., 2012; Guo et al., 2014), *Acidimicrobium ferrooxidans* (Zammit et al., 2012), and *Leptospirillum ferrooxidans* (Parro et al., 2007). The final up-regulated proteins involved in the stress response to chloride were an ATP-dependent Clp protease (4.3 ± 0.9 fold) that degrades proteins (Katayama et al., 1988); GroS (15.7 ± 3.7 fold) that acts in concert with GroE in the response to DNA mutation (Al Mamun et al., 2012); ADP-ribose pyrophosphatase, NudF (7.4 ± 2.5 fold) that if deleted increases sensitivity to heat stress (Krisko et al., 2014); and RNA polymerase-binding transcription factor, DksA (7.0 ± 2.0 fold) that is induced at low pH (Stancik et al., 2002).

Metabolic and electron transport proteins with a higher concentration in high salt conditions included rusticyanin (9.7 ± 2.3 fold) and cytochrome *c*_1_ (unique) involved in ferrous iron oxidation (Quatrini et al., 2009). As mentioned above, these proteins were likely used during proton exclusion from the cytoplasm. In addition, ATP synthase subunit b had an 8.2 ± 3.5 fold higher concentration in high salt conditions. In addition to synthesizing ATP, the *Enterococcus hirae* ATPase extrudes protons from the cytoplasm to regulate pH (Shibata et al., 1992) and increasing the concentration of subunit b may result in the same function.

Proteins with a statistically higher concentration in low salt conditions generally had much lower fold differences (Table 1). These proteins included OmpA (0.5 ± 0.3 fold) and AsmA (0.3 ± 0.1 fold) involved in OMP assembly that were likely decreased in high salt conditions to reduce pores in the outer membrane that allow influx of chloride, as has been reported for OmpC and OmpF in *E. coli* (Csonka and Hanson, 1991). In addition, the ATPase α-subunit (0.7 ± 0.2 fold) had a higher concentration in low salt conditions, potentially as the complex was being used to produce ATP rather than extrude protons (Shibata et al., 1992). In a similar vein, several central carbon metabolism (e.g., enolase; 0.4 ± 0.1 fold), Calvin-Benson-Bassham cycle (e.g., ribulose bisphosphate carboxylase large chain; 0.7 ± 0.3 fold), and ribosomal (e.g., 50S ribosomal protein L23, RplW; 0.5 ± 0.3 fold) proteins had higher concentrations as energy production via ferrous iron oxidation was likely utilized for cellular growth rather than as a response to osmotic and pH stress.

### *At. ferrooxidans* proteomic response to the presence of chloride

*At. ferrooxidans*^T^ response to growth in high (8 g/L) and low (0 g/L) salt conditions was investigated by two-dimensional polyacrylamide gel based proteomics (Supplemental File 4) that identified a total of 24 statistically valid up-regulated proteins during growth in high salt conditions (Supplemental File 5 with proteins discussed in the text in Table 2). *At. ferrooxidans*^T^ growth in high salt exhibited several similar strategies as employed by *Ac. prosperus*^T^ such as the increased abundance of peptidyl-prolyl *cis-trans* isomerase (two protein spots that were 2.8 and 2.5 fold up-regulated in 8 vs. 0 g/L salt) which is involved in outer membrane protein folding (Vertommen et al., 2009). Another three protein spots with increased abundance were identified as periplasmic solute binding proteins that are involved in the maintenance of the cell envelope integrity (2.8, 2.6, and 2.3 fold). However, the periplasmic solute binding protein also had a 3.1 higher concentration in low salt conditions suggesting that it had undergone regulation via post-translational modification. Several *At. ferrooxidans*^T^ stress proteins with higher concentrations in 8 g/L NaCl included heat shock protein Hsp20 (2.5 fold) that aids in reducing protein denaturation (Lindquist and Craig, 1988); ribosome recycling factor (4.0 fold) also observed when *Ac. prosperus*^T^ was cultured in high salt conditions; and a serine protease, DO/DeqQ family protein (2.0 fold) that has a chaperone function and also has a higher concentration in the *At. ferrooxidans* response to heat stress (Ribeiro et al., 2011). Finally, the major outer membrane protein 40 had 1.8 fold lower concentration in high salt conditions, potentially to reduce the influx of chloride (Csonka and Hanson, 1991).

**Table 2:** *****At. ferrooxidans***^**T**^ proteins with statistically supported altered abundance when grown in high or low NaCl concentration**.

**UniProt^a^**	**Protein**	***P*-value^b^**	**Fold^c^**
**UP-REGULATED IN HIGH SALT CONDITIONS**			
**Cell Envelope and Its Integrity**			
B7JA08	Survival protein SurA	0.049	3.4
B7J3E4	Periplasmic solute binding protein	0.038	2.8
B7J3E4	Periplasmic solute binding protein	0.04	2.6
B7J541	PpiC-type peptidyl-prolyl *cis-trans* isomerase	0.026	2.8
B7J541	PpiC-type peptidyl-prolyl *cis-trans* isomerase	3.9 E-04	2.5
B7J3E4	Periplasmic solute binding protein	0.007	2.3
**Stress**			
B7J9P4	Ribosome recycling factor	0.009	4.0
B7J4U6	Heat shock protein Hsp20	0.026	2.5
B7J942	Serine protease, DO/DeqQ family	0.123	2.0
**Metabolism and Energy Conservation**			
B7JBC9	Glyceraldehyde-3-phosphate dehydrogenase, type I	0.004	4.0
B7J3E6	Sulfur/pyrite/thiosulfate/sulfide-induced protein	0.001	2.9
B7J6R4	Enolase	0.026	2.2
B7JBC9	Glyceraldehyde-3-phosphate dehydrogenase, type I	0.050	2.0
**UP-REGULATED IN LOW SALT CONDITIONS**			
**Cell Envelope and Its Integrity**			
B7J3E4	Periplasmic solute binding protein	3.71 E-04	3.1
B7J8H1	Major outer membrane protein 40	0.005	1.8
**Stress**			
B7J7L4	Glycine cleavage system H protein	0.007	1.9
**Metabolism and Energy Conservation**			
P0C918	Rusticyanin (Form I)	0.006	2.5
B7J913	50S ribosomal protein L9	0.022	1.8

a *Uniprot accession number, refers to the identified protein within this database*.

b *Significance as calculated by ANOVA*.

c *Average fold up-regulation between the high and low salt proteomes*.

In contrast to the increase in rusticyanin seen in *Ac. prosperus*^T^ when cultured in high salt conditions, *At. ferrooxidans*^T^ had a 2.5 fold decrease implying a reduction in iron oxidation (Quatrini et al., 2009) as was demonstrated in the growth experiments, where a reduction of iron oxidation by 25% was observed.

### Rusticyanin tolerance to increased salt concentration

Iron oxidation in the well-studied acidophile *At. ferrooxidans*^T^ involves a protein complex that transfers electrons from iron to oxygen (Castelle et al., 2008; Li et al., 2015) and includes the copper protein rusticyanin encoded in the *rus* operon (Levicán et al., 2002). Rusticyanin is located in the periplasmic space where the pH is low. A cluster of genes has been detected in *Ac. prosperus* V6 (DSM 14174) that has similarity to the *rus* operon of *At. ferrooxidans*^T^ (Nicolle et al., 2009) and it is hypothesized that expression of the rusticyanin gene is actively involved in Fe oxidation, presumably in a similar way to that described for *At. ferrooxidans*^T^. However, a major difference in the two systems is that iron oxidation in *At. ferrooxidans*^T^ is inhibited by low concentrations of chloride (Blake et al., 1991; Harahuc et al., 2000), whereas chloride is required for expression of rusticyanin in *Ac. prosperus* V6 (Nicolle et al., 2009) and maximum iron oxidation in *Ac. prosperus*^T^ was seen at 20 g/L NaCl.

Using the rusticyanin gene of *At. ferrooxidans*^T^ (locus tag: AFE_3146) as a query, two rusticyanin genes, termed Form I and Form II (locus tags: Thpro_021557 and Thpro_020703, respectively) were predicted in the genome of *Ac. prosperus*^T^ (Ossandon et al., 2014). Relative to the rusticyanin of *At. ferrooxidans*^T^, Form I was detected with a score of 142, a query coverage of 100%, an *E*-value of 2e-48, and an identity of 46%. The same parameters for Form II were 116, 89%, 2e-38, and 43%. The extent of sequence similarity and coverage suggest that the two forms of Rus in *Ac. prosperus*^T^ are encoded by genes that are orthologs of *rus* from *At. ferrooxidans*^T^. Both Rus Forms I and II are predicted to contain signal peptides and to reside in the periplasm. If this is true, then they are most likely to be subjected to the low pH and high salt conditions typical for *Ac. prosperus*. However, the genetic contexts of the two Forms differ (Supplemental File 6). Form I is embedded in a gene cluster very similar to the classical *rus* operon of *At. ferrooxidans*^T^ (Valdes et al., 2008). This supports the hypothesis that Form I Rus is involved in iron oxidation in a manner similar to that described for *At. ferrooxidans*^T^. In contrast, the gene encoding Form II Rus is found as a singleton gene with no other known genes involved in iron oxidation in the gene neighborhood (Supplemental File 6). The function of this Rus remains unknown. However, because of its sequence similarity to Rus from *At. ferrooxidans*^T^, it can be speculated that it is also involved in iron oxidation, perhaps under different growth conditions from Form I Rus.

**Figure 4:**
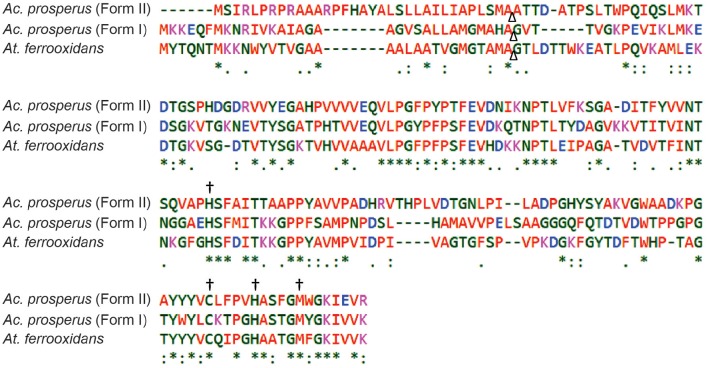
**Clustal Omega multiple alignment of rusticyanins from ***At. ferrooxidans***^**T**^ (locus tag: AFE_3146), ***Ac. prosperus***^**T**^ Form I (locus tag: Thpro_021557) and ***Ac. prosperus***^**T**^ Form II (locus tag: Thpro_020703)**. An ^*^ (asterisk) indicates positions which have a single, fully conserved residue. (A), (colon) indicates conservation between groups of strongly similar properties, a. (period) indicates conservation between groups of weakly similar properties. A Δ (triangle) indicates the position of cutting of the peptide signal using *At. ferrooxidans*^T^ as reference. The meaning of the colors is described in (Sievers et al. (2011), Mol Sys Bio 7:539). A ^†^ (dagger) indicates the four conserved amino acids that bind the copper ion.

As Form I Rus increases in abundance (9.7 ± 2.3 fold) when *Ac. prosperus*^T^ is subjected to high salt conditions, both its primary amino acid sequence and its predicted tertiary structure were interrogated for clues that might suggest how it maintains activity in high salt conditions. Form II Rus (no change in abundance with increasing salt concentration) and Rus from *At. ferrooxidans*^T^ (2.5 fold decrease) were included for comparison (Figure 4). Four critical amino acids (two histidines, one cysteine, and one methionine) have been shown to be ligands in the inner sphere coordinating the copper ion in Rus in *At. ferrooxidans*^T^ and many other members of the family of small blue copper proteins (Gray et al., 2000). The ligands Cys, Met, and one of the histidines are close to each other at the C terminal end in the primary sequence whereas the other histidine is far away from them in the amino acid chain. The loop length that connects these ligands has been shown to be important for coordination of the copper in related blue copper proteins (Gough and Chothia, 2004). Also, as observed in other small blue copper proteins including Rus from *At. ferrooxidans*^T^, both Form I and Form II Rus from *Ac. prosperus*^T^ are predicted to contain the so-called Greek key β-barrel (data not shown). This is a rigid structure formed by an extended network of hydrogen bonds and tertiary interactions between amino acid side chains (Gray et al., 2000). This rigidity is transmitted to the metal ion and is essential for electron transfer. As shown in Figure 4, these ligands, their relative positions in the primary amino acid sequence, and the length of the connecting loop are conserved in Forms I and II Rus from *Ac. prosperus* and in Rus from *At. ferrooxidans*^T^. Due to the conservation of these properties between the acidophilic *At. ferrooxidans*^T^ and the haloacidophilic *Ac. prosperus*^T^, it is unlikely that they contribute to salt tolerance in Rus Form I (and perhaps Form II).

**Figure 5:**
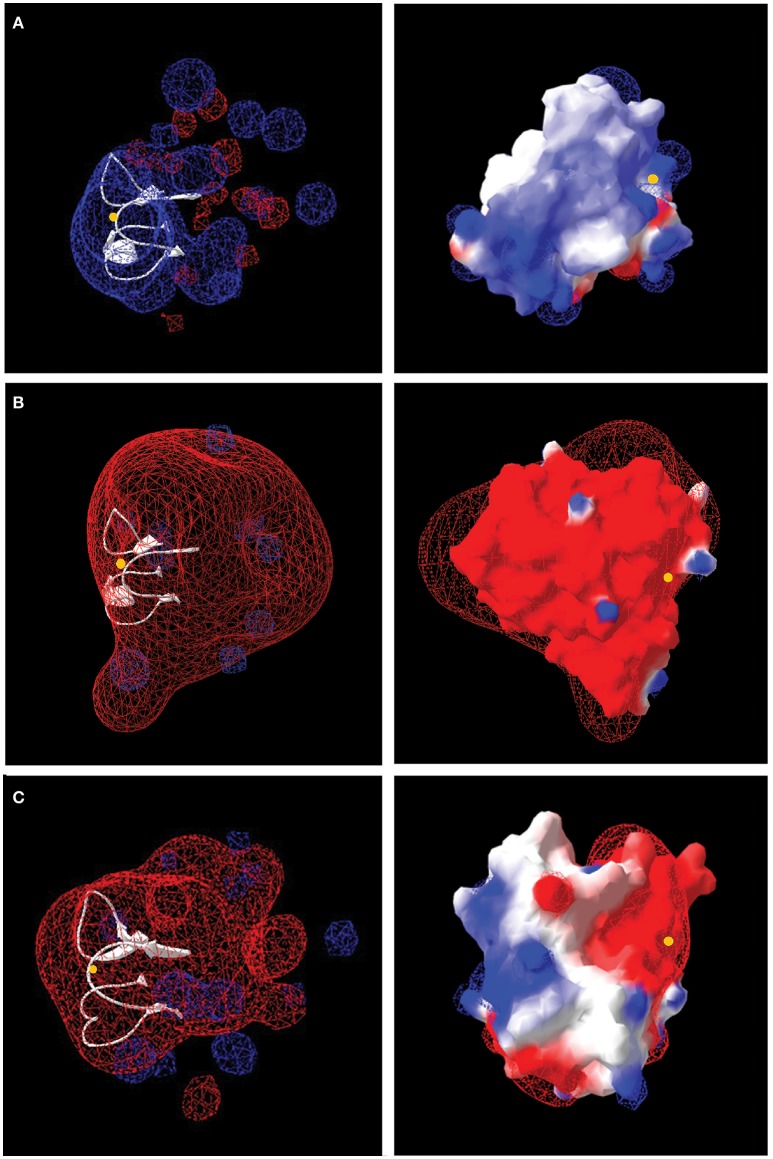
**Models of the electrostatic surface potential of rusticyanin of: (A)** *At. ferrooxidans*^T^; **(B)** *Ac. prosperus*^T^ Form I, and **(C)** *Ac. prosperus*^T^ Form II. The surface is colored according to the protein electrostatic potential from red (negative) to blue (positive); the copper ion is shown as a yellow dot. The models on the left hand side are rendered transparent to show (in white) the critical protein folds that binds the copper ion. The models on the right have been rotated 180° (*y*-axis) compared with the models on the left to provide a different perspective.

The number and distribution of positively (His, Lys, and Arg) and negatively charged (Asp and Glu) amino acids in Rus Forms I and II differ from that observed for Rus of *At. ferrooxidans*^T^ (Figure 4). This is in agreement with an observation made earlier for Rus from *Ac. prosperus* V6 (Nicolle et al., 2009). In order to examine whether these differences in charged amino acids could affect the surface electrostatic potential of the different Rus, three dimensional models of the structures of Rus Forms I and II were constructed using the experimentally determined structure of rusticyanin from *At. ferrooxidans*^T^ (PDB “1RCY”) as a template (Walter et al., 1996). The predicted surface electrostatic potentials of Rus Forms I and II of *Ac. prosperus*^T^ (Figures 5B,C) are significantly more negative compared to that of Rus from *At. ferrooxidans*^T^ (Figure 5A). In the case of Form I Rus, this negative electrostatic potential is widely distributed over the surface of the entire molecule, including around the copper ion. In contrast, in Form II, it is principally distributed around the copper ion. It has been well-established that the electrostatic field directly influences the electrostatic properties of the metal-binding site of blue copper proteins, being a major determinant of the redox potential of the copper ion (Olsson et al., 2003). It is possible that the noticeable negative shift in surface electrostatic potential of Rus Forms I and II could help stabilize them in high salt conditions and assist in the maintenance of an appropriate redox potential of the copper ion. It could also help to repel negatively charged chloride ions in the immediate environment of the proteins.

The increased negative surface electrostatic potential of Form I rusticyanin of *Ac. prosperus*^T^ likely affects its interactions with its redox partners that, based upon amino acid sequence similarities and gene neighborhood conservation, are predicted to be the same as in *At. ferrooxidans*^T^ [i.e., a high molecular weight *c*-type cytochrome Cyc2 located in the external membrane, a periplasmic diheme cytochrome *c* Cyc1, and a periplasmic diheme cytochrome Cyc_42_ (Cyc1A; Bruscella et al., 2007; Castelle et al., 2008)]. Although the potential changes evidently still permit electron transfer, their nature requires experimental verification. The redox partners of Rus Form II are not known.

Other changes in amino acid sequence between Rus of *At. ferrooxidans*^T^ and *Ac. prosperus*^T^ might reveal clues regarding stabilization and activity of Rus at high salt concentrations such as changes in the outer coordination sphere (Cascella et al., 2006; Warren et al., 2012), but these await discovery and investigation. Although a reasonable argument can be made that an increase in the negativity of the surface potential of Rus Form I (and Form II) could help explain salt tolerance perhaps by modulating the environment of copper ion and very likely by affecting its interaction with redox partners, significant effort is still required to understand and experimentally validate these ideas. However, the current suggestions do lead to testable hypotheses and can be used a basis for guiding future research.

### Model of *Ac. prosperus* responses to chloride

**Figure 6:**
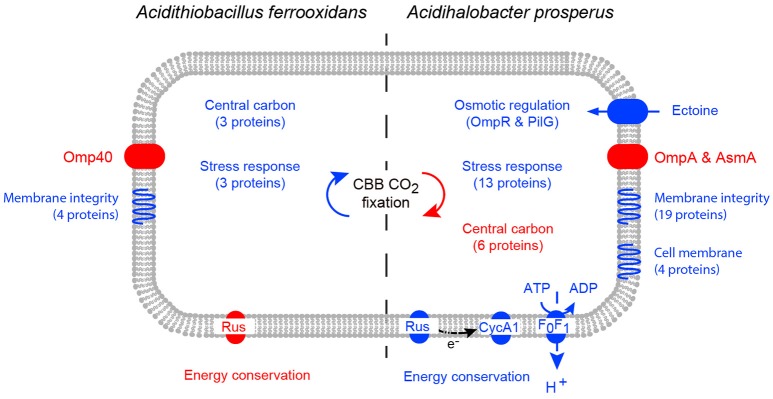
**A model of the cellular response of ***At. ferrooxidans***^**T**^ (left) and ***Ac. prosperus***^**T**^ (right) to increased NaCl levels**. Increases in protein abundance in high NaCl conditions are represented by blue and decreases in protein abundance in high NaCl conditions are represented by red. Differential protein expression was determined by 2D-PAGE for *At. ferrooxidans*^T^ and iTraq for *Ac. prosperus*^T^.

When challenged by elevated salt concentration, acidophiles experience both osmotic stress and an acidification of the intracellular pH (reviewed in Zammit and Watkin, 2016). This is due to a collapse of the inside positive membrane potential as a result of Cl^−^ crossing the cell membrane, leading to an influx of protons. Notwithstanding the caveat that the iTRAQ analysis of *Ac. prosperus*^T^ in high salt will identify many more proteins than the 2D-PAGE analysis of *At. ferrooxidans*^T^, the response of the two species were distinct (Figure 6). *At. ferrooxidans*^T^ responded to even low levels of Cl^−^ with a generalized stress response and decreased iron oxidation which was confirmed by a reduced abundance of the protein rusticyanin. However, despite the reduced ability to generate energy there was an increase in central carbon metabolism and carbon fixation. The most significant responses to increased salt concentration by *Ac. prosperus*^T^ were an increase in abundances of osmotic stress regulators; uptake of the compatible solute ectoine protein and increased iron oxidation as confirmed by the raised abundance of the proteins rusticyanin and cytochrome *c*_1_ that consumes cytoplasmic protons and/or provides reducing power for the stress response. Both central carbon metabolism and carbon fixation decreased suggesting the increased ability to generate energy is utilized for the potential efflux of protons via the F_0_F_1_ ATPase at the expense of ATP suggested by the greater abundance of the ATP synthase subunit b.

## Author contributions

EW, MD, CB, and DH conceived and designed the experiments. EW, TM, KM, and ML performed the experiments. MD, EW, DH analyzed the data. EW, MD, DH, RS, and CJ contributed to the reagents/materials/analysis tools. MD, EW, and DH wrote the paper. All authors read and approved the final manuscript.

### Conflict of interest statement

The authors declare that the research was conducted in the absence of any commercial or financial relationships that could be construed as a potential conflict of interest. The reviewer CSD declared a past co-authorship with one of the authors DH to the handling Editor, who ensured that the process met the standards of a fair and objective review. The reviewer SH and the handling Editor declared their shared affiliation, and the handling Editor states that the process nevertheless met the standards of a fair and objective review.
