# Supplementary material for: Multiple Osmotic Stress Responses in Acidihalobacter prosperus Result in Tolerance to Chloride Ions
Source: Front Microbiol. 2017 Jan 5;7:2132. doi: 10.3389/fmicb.2016.02132 (PMC5216662; doi:10.3389/fmicb.2016.02132)

Supplementary Material

Multiple osmotic stress responses in *Acidihalobacter prosperus* results in a greater tolerance to chloride ions than in *Acidithiobacillus ferrooxidans*

Mark Dopson, David S Holmes, Marcelo Lazcano, Timothy J McCredden, Christopher Bryan, Kieran T Mulroney, Robert Steuart, Connie Jackaman, Elizabeth LJ Watkin*

***Correspondence:**

Elizabeth L. Watkin (e.watkin@curtin.edu.au)

Supplemental File 1. Confirmation of difference in fluorescence intensity between live and dead cells in *At. ferrooxidans*[T] a) and *Ac. prosperus*[T] b). The effect of increasing NaCl on the viability of *At. ferrooxidans*[T] at 0 and 3.5 g/L NaCl c) and *Ac. prosperus*[T] at 12.5 and 30 g/L NaCl d).

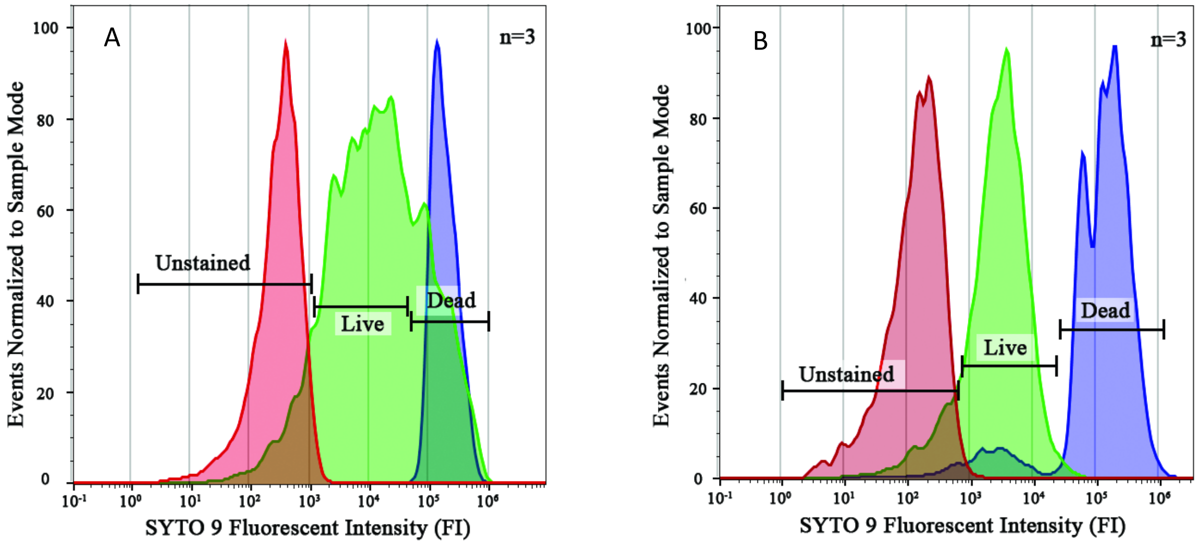

	Sample Name	SYTO9 MFI
	At. ferrooxidans - No Stain	3.00 x 10^2
	At. ferrooxidans - Untreated	5.23 x 10^4
	At. ferrooxidans - Heat Treated	1.86 x 10^5

	Sample Name	SYTO9 MFI
	Ac. prosperus - No Stain	1.75 x 10^2
	Ac. prosperus - Untreated	4.41 x 10^3
	Ac. prosperus - Heat Treated	1.65 x 10^5

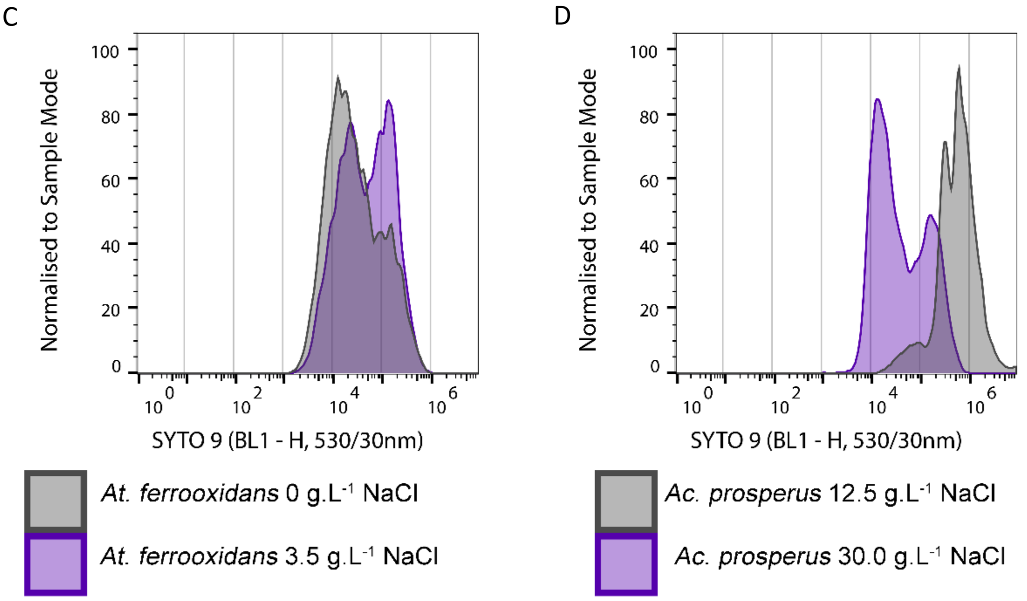

Supplemental File 2. Complete list of *Ac prosperus*^{T} differentially expressed soluble proteins identified by iTRAQ analysis in high salt (116 & 117) and low salt (114 & 115). An explanation of the different scores is given below.

Unused (ProtScore) A measure of the protein confidence for a detected protein, calculated from the peptide confidence for peptides from spectra that have not already been completely "used" by higher scoring winning proteins. A "good" Unused ProtScore is one that corresponds to the level of confidence you require in your results. For 95% confidence, the required Unused ProtScore is 1.3.

Total (ProtScore) A measure of the total amount of evidence for a detected protein. The Total ProtScore is calculated using all of the peptides detected for the protein. The Total ProtScore does not indicate the percent confidence for the identification of a protein.

% Cov (Coverage) The percentage of matching amino acids from identified peptides having confidence greater than 0 divided by the total number of amino acids in the sequence.

Ratio The average ratio for the protein, relative to the 114 and 115.

p-value For each protein ratio reported the program calculates a p-value to help you assess whether changes in protein expression are real or not. A p-value is a standard statistical metric in hypothesis testing. The p-value reports the probability that the null hypothesis "the observed value is different from unity by chance" is true. P-values range from 0 to 1.

Color coding The quantitative ratios of identified proteins are colour coded to indicate differential expression. Red indicates up-regulation and blue indicates down-regulation. The intensity of the colouring indicates the certainty of the differential expression, not the magnitude of the change. For example, the more certain the up-regulation, the more red the cells; the more certain the down-regulation, the more blue the cells. Note: The coloring is only indicative of altered expression levels, and is determined by the p-value not by the size of the ratio.

Color codes	P-value	Ratio
Dark red	< 0.001	> 1
Medium red	0.001 - < 0.01	> 1
Light red	0.01 - < 0.05	> 1
No color	>= 0.05	Any
Light blue	0.01 - < 0.05	< 1
Medium blue	0.001 - < 0.01	< 1
Dark blue	< 0.001	< 1

Unused	Total	% Cov	Accession #	Peptides (95%)	116:114	PVal 116:114
45.99	45.99	62.8	contig00006_-_ectoine/hydroxyectoine_ABC_transporter_solute-binding_protein	44	54.4503	0.0002
2	2	4.4	contig00006_-_Flagellin_protein	1	23.7684	0.1235
1.74	2	2	contig00011_-_adenylosuccinate_synthetase	1	17.2187	0.0868
2.38	2.38	19.2	contig00001_-_nudF,_ADP-ribose_pyrophosphatase_NudF	2	12.0226	0.0332
2	2	9.4	contig00004_-_helix_turn_helix_multiple_antibiotic_resistance_protein	1	11.4815	0.3233
3.82	3.82	5.6	contig00003_-_ATP_synthase_epsilon_chain	2	10.3753	0.3024
12.23	12.25	39.9	contig00005_-_Toluene_tolerance,_Ttg2	9	10.3753	0.0006
41.87	41.87	51.3	contig00002_-_dnaK,_molecular_chaperone_DnaK	33	9.8175	0
2	2	8.5	contig00009_-_ZnuA,_ABC-type_Zn2+_transport_system,_periplasmic_component/surface_adhesin	2	9.5499	0.2027
22.23	22.23	62.5	contig00015_-_10_kDa_chaperonin	23	9.2045	0.0003
2.08	2.08	17.1	contig00016_-_AhpC/TSA_family	2	9.1201	0.0019
6	6	11	contig00013_-_type_IV_pilus_biogenesis/stability_protein_PilW	3	8.3176	0.0431
2.3	2.3	20.5	contig00014_-_Flagellar_basal_body-associated_protein_FliL	1	8.2414	0.2
12.12	12.12	15.8	contig00002_-_ATP-dependent_zinc_metalloprotease_FtsH	7	8.1658	0.0002
34.54	34.58	61.5	contig00008_-_Rusticyanin_protein	52	7.7983	0.0282
2	2	16.7	contig00002_-_Escherichia_coli_YhbY_is_associated_with_pre-50S_ribosomal_subunits,_ribosome_assembly	1	7.5858	0.2427
6	6.01	11.9	contig00013_-_outer_membrane_assembly_lipoprotein_YfgL	7	7.5162	0.0565
23.4	23.4	30.2	contig00025_-_BtuB,_Outer_membrane_cobalamin_receptor_protein	19	7.4473	0.0013
4.14	4.15	22.8	contig00002_-_Thioredoxin_domain-containing_protein	2	6.6069	0.0759
2	2	5.3	contig00010_-_heavy_metal_response_regulator	1	6.4863	0.1309
16.05	16.05	41.1	contig00008_-_hypothetical_protein	12	6.2517	0.0089
12.39	12.39	42.7	contig00007_-_Protein_PilG	7	6.1376	0.0086
12.05	12.05	29.1	contig00008_-_CyoA,_Heme/copper-type_cytochrome/quinol_oxidases,_subunit_2	10	6.1376	0.0968
26.12	26.12	47.3	contig00001_-_2,3,4,5-tetrahydropyridine-2,6-dicarboxylate_N-succinyltransferase	16	6.0256	0
2.02	2.02	14.3	contig00001_-_ATP-dependent_Clp_protease_adapter_protein_ClpS	1	6.0256	0.0342
10	10	53.3	contig00002_-_heat_shock_protein_GrpE	7	6.0256	0.011
2	2	7.9	contig00005_-_Sigma_54_modulation_protein_/_S30EA_ribosomal_protein	1	5.9704	0.1595
4.57	4.57	24.9	contig00007_-_Pilus_assembly_protein,_PilP	3	5.7544	0.3026
35.75	35.75	83.2	contig00026_-_Major_outer_membrane_protein_P.IB	66	5.7016	0.0089
21.56	21.59	45.3	contig00004_-_HdrA,_Heterodisulfide_reductase,_subunit_A_and_related_polyferredoxins	14	5.6494	0.0037
30.46	30.46	43.1	contig00002_-_Probable_parvulin-type_peptidyl-prolyl_cis-trans_isomerase	24	5.4954	0
3.14	3.14	8.9	contig00002_-_Lon,_ATP-dependent_Lon_protease,_bacterial_type	3	5.3456	0.0199
97.08	97.08	60	contig00015_-_60_kDa_chaperonin	107	5.2966	0
20.56	20.56	48	contig00002_-_signal_peptidase_I	14	5.2481	0.0019
22.46	22.46	61.5	contig00056_-_Gram-negative_porin	47	5.2	0.0267
10	10	20.1	contig00013_-_Cytoskeleton_protein_RodZ	5	5.0582	0.0305
38.78	38.78	37.1	contig00008_-_Putative_outer_membrane_cytochrome_c	50	4.6559	0.1328

17.6	17.6	39.5	contig00006_-_LysM_domain/BON_superfamily_protein	34	4.529	0.0024
2	4.01	8	contig00009_-_Hemolysin_secretion_protein	2	4.529	0.1605
16	16	42.1	contig00013_-_Transmembrane_protein	9	4.529	0.007
2.43	2.43	34.3	contig00001_-_Lipoprotein	2	4.4055	0.331
12.74	12.74	26.3	contig00004_-_tol-pal_system_protein_YbgF	11	4.3251	0.0172
39.73	39.73	20.3	contig00011_-_FimV,_Tfp_pilus_assembly_protein_FimV	26	4.2855	0.0037
16	16	48	contig00030_-_50S_ribosomal_protein_L7/L12	13	4.2855	0.0032
2.33	2.33	8.5	contig00025_-_Cobalamin_adenosyltransferase	2	4.2462	0.1671
4.12	4.12	28.6	contig00001_-_putative_nucleotide-binding_protein	3	4.1687	0.1255
8	8	32.3	contig00016_-_reactive_intermediate/imine_deaminase	6	4.1687	0.0467
4	4	27.6	contig00026_-_2-nonaprenyl-3-methyl-6-methoxy-1,4-benzoquinol_hydroxylase	2	4.0926	0.7238
5.44	5.44	26.7	contig00006_-_arsenate_reductase	5	4.0551	0.2791
31.17	32.54	35	contig00002_-_rpsA,_30S_ribosomal_protein_S1	19	4.0179	0
4.17	4.17	21.9	contig00002_-_oligoribonuclease	3	3.9446	0.1363
14	14	15.3	contig00024_-_Porins_form_aqueous_channels_for_diffusion_of_hydrophillic_molecules_across_the_outer_membrane	8	3.9446	0.002
4.06	4.06	14.3	contig00021_-_inositol_monophosphatase	3	3.8726	0.2458
4.3	4.3	22.5	contig00001_-_lolA,_lipoprotein_chaperone	3	3.8371	0.1118
4.05	4.05	18	contig00003_-_F0F1_ATP_synthase_subunit_delta	2	3.8371	0.2745
8.54	8.54	33.9	contig00006_-_single-stranded_DNA-binding_protein	5	3.8371	0.1131
4.75	4.75	16.1	contig00014_-_DdpA,_ABC-type_dipeptide_transport_system,_periplasmic_component	3	3.8371	0.046
11.86	11.86	28	contig00004_-_peptidoglycan-associated_lipoprotein	21	3.767	0.0234
12.47	12.47	31.9	contig00002_-_greA,_transcription_elongation_factor_GreA	8	3.7325	0.0378
10.7	10.7	28.6	contig00006_-_Uncharacterized_enzyme_involved_in_biosynthesis_of_extracellular_polysaccharides	12	3.7325	0.0709
8	8	24.8	contig00009_-_Conserved_hypothethical_protein,_UCP019302	4	3.6644	0.0225
4.18	4.18	11.5	contig00016_-_ATP-dependent_protease_subunit_HslV	3	3.6308	0.2728
12.04	12.04	43.3	contig00017_-_Putative_lipoprotein	9	3.6308	0.0079
2	17.88	41.7	contig00027_-_Gram-negative_porin	28	3.5975	0.2218
2	2	5.2	contig00012_-_SlpA,_FKBP-type_peptidyl-prolyl_cis-trans_isomerases_2	3	3.5645	0.2527
15.72	15.72	57.1	contig00001_-_RNA_polymerase-binding_transcription_factor_DksA	10	3.4995	0.0104
6	6	21.2	contig00030_-_nusG,_transcription_antitermination_protein_NusG	5	3.4995	0.2156
17.71	17.71	28.4	contig00003_-_Periplasmic_binding_protein	16	3.4356	0.0151
13.55	13.55	71.6	contig00006_-_50S_ribosomal_protein_L29	9	3.4356	0.018
6	6	25.8	contig00010_-_secreted_protein	3	3.4356	0.1386
10.02	10.02	55.7	contig00001_-_3-ketoacyl-(Acyl-carrier-protein)_reductase	7	3.4041	0.0718
4.43	4.43	25.2	contig00002_-_Protein_GrpE	4	3.4041	0.3671
2	2	7.2	contig00011_-_ABC-type_uncharacterized_transport_system,_auxiliary_component	1	3.4041	0.1952
6.68	6.68	31.9	contig00021_-_yajC,_preprotein_translocase_subunit_YajC	5	3.4041	0.2436
6.13	6.13	32.1	contig00005_-_Thiol:disulfide_interchange_protein_DsbA	3	3.3113	0.1003
20.36	20.36	35.6	contig00004_-_Chaperone_SurA	11	3.2509	0.002

5.31	5.31	18.7	contig00016_-_Curved_DNA-binding_protein	3	3.2509	0.0864
7.79	7.79	13.9	contig00002_-_nusA,_transcription_elongation_factor_NusA	4	3.2211	0.0442
2	2	9.4	contig00010_-_Probable_ATP-dependent_RNA_helicase_ddx27	2	3.2211	0.3888
2.23	2.23	27	contig00002_-_Cupredoxin-like_domain	1	3.1915	0.2213
2	2	5.1	contig00005_-_Cell_division_protein_FtsA	1	3.1915	0.2053
10.71	10.85	22.1	contig00001_-_Ubiquinol_oxidase_subunit_2	9	3.1623	0.1487
8	8	23.1	contig00003_-_ATP_synthase_subunit_b	5	3.1333	0.076
4.12	4.14	24.8	contig00006_-_This_family_is_most_closely_related_to_the_GT1_family_of_glycosyltransferases	3	3.1333	0.2757
16	16	23.3	contig00027_-_Dienelactone_hydrolase_and_related_enzymes	12	3.1333	0.0141
8.86	8.92	24.1	contig00019_-_Outer_membrane_beta-barrel_domain_protein	7	3.1046	0.2551
2.01	2.01	9.6	contig00001_-_Predicted_transcriptional_regulator	1	3.0479	0.2127
8.19	8.19	33.9	contig00010_-_Membrane_protein_involved_in_aromatic_hydrocarbon_degradation	22	3.0479	0.1329
15.33	15.33	34	contig00003_-_preprotein_translocase_subunit_SecB	9	3.02	0.1131
6.77	6.77	14.5	contig00003_-_HemX,_Uncharacterized_enzyme_of_heme_biosynthesis	7	2.9923	0.0812
4.47	4.47	15.9	contig00011_-_L,D-transpeptidase_catalytic_domain	4	2.9648	0.0767
2	2	2.5	contig00018_-_Outer_membrane_protein_beta-barrel_domain	1	2.9376	0.4291
8.85	8.85	28.5	contig00010_-_50S_ribosomal_protein_L9	6	2.9107	0.0487
23.71	23.71	57.6	contig00006_-_rplF,_50S_ribosomal_protein_L6	20	2.884	0.0305
21.35	21.35	66.5	contig00001_-_frr,_ribosome_recycling_factor	15	2.8576	0.0014
2	2	4.1	contig00010_-_Outer_membrane_protein_transport_protein	1	2.8576	0.2253
8.08	8.08	18.9	contig00026_-_Protein_CbbQ	5	2.8576	0.0549
2.07	2.08	7.5	contig00012_-_Asparaginase	2	2.8314	0.2266
27.28	27.28	23.7	contig00030_-_elongation_factor_G	16	2.7797	0.1193
4	4	4.5	contig00006_-_glpC,_sn-glycerol-3-phosphate_dehydrogenase_subunit_C	2	2.729	0.234
2	2	11.8	contig00009_-_iron-sulfur_cluster_insertion_protein_ErpA	2	2.6792	0.2387
4	4	6.9	contig00020_-_Succinate-semialdehyde_dehydrogenase_[NADP(+)]_GabD	3	2.6792	0.2427
2.88	2.88	21.5	contig00002_-_peptidyl-prolyl_cis-trans_isomerase_B	4	2.6062	0.2852
8.05	8.05	42.2	contig00007_-_Protein_PilH	5	2.6062	0.1098
2.48	2.48	16.5	contig00004_-_Protein_TolB	2	2.5823	0.349
6	6	25.6	contig00005_-_YfaZ_family_protein	5	2.5586	0.1827
6.86	6.86	19.4	contig00007_-_Fimbrial_assembly_protein_PilQ	4	2.5586	0.2504
4.92	4.92	7.7	contig00010_-_pyruvate_dehydrogenase	3	2.5586	0.1582
2	2	3.6	contig00016_-_Sterol-binding_domain_protein	1	2.5586	0.4571
13.08	13.08	21.1	contig00036_-_cysteine_desulfurase	7	2.5586	0.0113
7.13	7.13	13.8	contig00005_-_Fimbrial_protein_P9-2	11	2.5351	0.2023
4	4	18.3	contig00001_-_PII-like_signaling_protein	2	2.5119	0.2885
10.13	10.13	24.9	contig00002_-_Universally_conserved_protein	8	2.5119	0.0108
2	2	8.5	contig00015_-_moaA,_molybdenum_cofactor_biosynthesis_protein_A	1	2.5119	0.2483
2.98	2.98	14.8	contig00007_-_DNA_topoisomerase_1	2	2.4889	0.2198

4.17	4.17	17.9	contig00002_-_Tetratricopeptide_TPR_2_repeat_protein	2	2.466	0.1596
2.51	2.51	19.4	contig00004_-_Sulfur_oxidation_protein_SoxY	2	2.466	0.4313
6.02	6.02	14.1	contig00001_-_rne,_ribonuclease_E	3	2.421	0.8732
17.35	17.35	53.5	contig00001_-_tsf,_elongation_factor_Ts	14	2.3988	0.0075
8.45	10.79	15.4	contig00006_-_Outer_membrane_protein_assembly_factor_BamC	15	2.3988	0.2734
8.49	8.49	24	contig00014_-_Chemotaxis_phosphatase,_CheZ	6	2.3988	0.1123
2.02	2.02	5	contig00016_-_dihydrolipoamide_dehydrogenase	1	2.3988	0.2644
14.02	14.02	29.1	contig00003_-_F0F1_ATP_synthase_subunit_alpha	10	2.3768	0.3822
4.91	4.91	32.2	contig00016_-_IbpA,_Molecular_chaperone	4	2.3768	0.2718
2.74	2.74	8.8	contig00007_-_DNA_topoisomerase_I	2	2.2909	0.5901
2	2	9.4	contig00027_-_Thioredoxin-like_protein	2	2.2909	0.507
4.32	4.33	16.3	contig00005_-_ATP-dependent_chaperone_ClpB	2	2.2699	0.3499
32.43	32.43	62.1	contig00006_-_Rubrerythrin_protein	66	2.2699	0.0151
2.01	2.01	9.9	contig00010_-_dihydroxy-acid_dehydratase	1	2.2699	0.2807
4.03	4.03	17.2	contig00016_-_rph,_ribonuclease_PH	2	2.2699	0.3322
10	10	32.9	contig00013_-_ndk,_mulitfunctional_nucleoside_diphosphate_kinase/apyrimidinic_endonuclease/3	10	2.2491	0.0412
2.14	2.14	17.7	contig00026_-_Indole-3-glycerol_phosphate_synthase	1	2.2491	0.4643
6	6	26.9	contig00001_-_Elongation_factor_Ts	4	2.2284	0.2592
2.32	2.32	45.1	contig00006_-_rplN,_50S_ribosomal_protein_L14	2	2.2284	0.3248
11.37	11.37	22.7	contig00011_-_HflK_protein	8	2.2284	0.2907
4	4	17.9	contig00016_-_rpmE,_50S_ribosomal_protein_L31	3	2.2284	0.3669
8.52	8.52	15.1	contig00016_-_tolC,_outer_membrane_channel_protein	10	2.208	0.0682
13.72	13.72	50.5	contig00004_-_Sulphur_oxidation_protein_SoxZ	16	2.1677	0.0323
6	6	21	contig00026_-_Predicted_redox_protein,_regulator_of_disulfide_bond_formation	4	2.1677	0.2989
23.3	23.3	60.1	contig00010_-_30S_ribosomal_protein_S6	15	2.1478	0.0036
4	4	23.7	contig00014_-_Uncharacterized_BCR,_YaiI/YqxD_family_COG1671	2	2.1478	0.2991
4	6	23	contig00016_-_Putative_phosphoribosylformimino-5-aminoimidazole_carboxamide_ribotide_isomerase	3	2.1281	0.3052
4	4	8.3	contig00020_-_Outer_membrane_beta_barrel_protein	2	2.1086	0.2121
19.7	19.7	30.4	contig00022_-_pyruvate_kinase	17	2.1086	0.0344
5.1	5.1	39.9	contig00015_-_Dihydroneopterin_aldolase	3	2.0701	0.0022
7.92	7.92	38.8	contig00005_-_Ttg2C,_ABC-type_transport_system_involved_in_resistance_to_organic_solvents,_periplasmic_component	4	2.0324	0.1934
4	4	30.2	contig00013_-_RNA_recognition_motif	2	2.0324	0.3168
4.11	4.11	23.7	contig00007_-_Yqey-like_protein	5	1.9953	0.3744
6	6	10.2	contig00017_-_hypothetical_protein	3	1.9953	0.4026
2	2	11.1	contig00001_-_Cytochrome_c4	1	1.9588	0.3303
8.25	8.25	37.3	contig00001_-_hypothetical_protein	5	1.9588	0.6295
2	2	18.5	contig00003_-_ispH,_4-hydroxy-3-methylbut-2-enyl_diphosphate_reductase	1	1.9588	0.3556
4.05	4.05	18.2	contig00008_-_ubiquinol-cytochrome_c_reductase,_iron-sulfur_subunit	2	1.9588	0.1371

68.35	68.35	46.4	contig00002_-_NADH_dehydrogenase	48	1.9231	0.5303
3.05	3.05	37.9	contig00004_-_glycine_cleavage_system_protein_H	5	1.9231	0.5259
8	8	26	contig00017_-_thioredoxin_protein	6	1.9231	0.2877
4	4	24.8	contig00006_-_iojap-like_ribosome-associated_protein	2	1.9055	0.2438
2	2	4.6	contig00006_-_LPS-assembly_lipoprotein_LptE	1	1.9055	0.344
14.7	14.7	30.8	contig00004_-_tolB,_translocation_protein_TolB	14	1.888	0.0643
2	8	25	contig00005_-_Nitrogen_regulatory_protein_P-II	5	1.888	0.5447
2.02	2.02	14.9	contig00002_-_OlmA,_Outer_membrane_lipoprotein_OmlA	2	1.8707	0.2946
16.81	16.81	37.4	contig00012_-_50S_ribosomal_protein_L25/general_stress_protein_Ctc	15	1.8535	0.1818
4.15	4.15	23	contig00026_-_Bacterioferritin_protein	3	1.8365	0.3283
2.06	2.06	7	contig00015_-_dipZ,_thiol:disulfide_interchange_protein_precursor	1	1.803	0.3735
11.02	11.02	36.9	contig00015_-_Thioredoxin_protein	9	1.803	0.3173
2	2	5.4	contig00016_-_gmk,_guanylate_kinase	1	1.803	0.5279
2.28	2.28	19.8	contig00003_-_30S_ribosomal_protein_S20	2	1.7865	0.3731
6	6	10	contig00006_-_peptidase_PmbA	3	1.7865	0.4118
6.01	6.02	32.7	contig00006_-_ATPase_with_chaperone_activity,_ATP-binding_subunit	3	1.7701	0.5313
6.01	6.01	14	contig00015_-_Disulphide_bond_corrector_protein_DsbC	5	1.7701	0.5052
5.62	5.62	11.6	contig00005_-_argJ,_bifunctional_ornithine_acetyltransferase/N-acetylglutamate_synthase_protein	3	1.7539	0.4874
2	2.01	14.3	contig00007_-_Competence_protein_A	1	1.7539	0.3492
6.57	6.57	34.2	contig00006_-_Cytochrome_c	5	1.7378	0.2884
2	2	24.8	contig00002_-_Dienelactone_hydrolase_family	1	1.7219	0.3731
1.92	2	10.9	contig00007_-_Putative_cell_wall_biogenesis_protein	1	1.7219	0.4117
2	2	11.7	contig00002_-_FHA_domain	4	1.7061	0.5345
4.63	4.63	12.6	contig00003_-_PKD_domain_containing_protein	3	1.7061	0.1654
2	2	9.7	contig00001_-_ubiquinol-cytochrome_c_reductase,_iron-sulfur_subunit	1	1.6904	0.4031
2	2	8.6	contig00004_-_Lipoate_regulatory_protein_YbeD	1	1.6904	0.4026
3.66	3.66	9	contig00004_-_SuhB,_Archaeal_fructose-1,6-bisphosphatase_and_related_enzymes	2	1.6904	0.8749
8.06	8.06	51.2	contig00001_-_NADPH-dependent_7-cyano-7-deazaguanine_reductase	4	1.6749	0.8048
4	4	16.9	contig00005_-_PTS_IIA-like_nitrogen-regulatory_protein_PtsN	2	1.6749	0.6241
2.32	2.33	21.9	contig00008_-_Uncharacterized_ACR,_YkgG_family_COG1556	1	1.6749	0.4079
4	4.04	8.2	contig00015_-_BisC,_Anaerobic_dehydrogenases,_typically_selenocysteine-containing	2	1.6749	0.1855
4.05	4.05	13	contig00002_-_Cytidylate_kinase-like_family	2	1.6596	0.4309
2	2	25.3	contig00013_-_Chaperone_protein_HscA_homolog	1	1.6596	0.3979
10.97	10.97	18	contig00005_-_Thiazole_synthase	8	1.6444	0.7544
2.36	2.37	5.6	contig00002_-_clpX,_ATP-dependent_protease_ATP-binding_subunit_ClpX	1	1.6293	0.4217
2	2	5.5	contig00013_-_RNA_polymerase_sigma-70_factor,_TIGR02943_family	1	1.6293	0.4263
4	4.01	27.5	contig00024_-_3-hydroxydecanoyl-(acyl_carrier_protein)_dehydratase	2	1.6293	0.4542
8.15	8.15	55.8	contig00006_-_rpsJ,_30S_ribosomal_protein_S10	5	1.6144	0.3788
1.92	2	4.7	contig00002_-_NADH-quinone_oxidoreductase_subunit_J	1	1.5996	0.6658

4	4	21.7	contig00015_-_hypothetical_protein	2	1.5704	0.5601
2	2.01	13.3	contig00001_-_ihfA,_integration_host_factor_subunit_alpha	1	1.5417	0.4592
2	2	4.9	contig00001_-_UDP-N-acetylglucosamine_acyltransferase	1	1.5417	0.4623
10	10	64.8	contig00006_-_rplX,_50S_ribosomal_protein_L24	8	1.5417	0.4008
4.6	4.6	51.7	contig00010_-_hypothetical_protein	11	1.5417	0.7499
2	2	10.4	contig00015_-_acetyl-CoA_carboxylase_biotin_carboxylase_subunit	1	1.5417	0.4619
2	2.01	6.8	contig00020_-_Curved_DNA-binding_protein	1	1.5417	0.4563
2	2	11.1	contig00025_-_AmpD_protein	1	1.5417	0.4661
12.83	12.83	48.4	contig00002_-_bacterial_(prokaryotic)_histone_like_domain	18	1.5276	0.5648
3.05	3.09	9.4	contig00003_-_glyS,_glycyl-tRNA_synthetase_subunit_beta	2	1.5276	0.4934
2	2	21.4	contig00015_-_CopZ,_Copper_chaperone	1	1.5276	0.3188
4.78	4.78	52.8	contig00001_-_infA,_translation_initiation_factor_IF-1	3	1.5136	0.38
6.01	6.01	13.3	contig00008_-_Cytochrome_c4	4	1.5136	0.3983
2	2	11.7	contig00009_-_Vitamin_B12_dependent_methionine_synthase,_activation_domain	1	1.5136	0.4715
6.18	6.18	13.6	contig00015_-_rho,_transcription_termination_factor_Rho	3	1.5136	0.5588
30.92	30.92	37.6	contig00022_-_Ribulose_bisphosphate_carboxylase_large_chain	26	1.5136	0.5135
6.02	6.02	27.5	contig00002_-_elongation_factor_P	3	1.4997	0.2099
2	2	23	contig00004_-_TolA_protein	2	1.4997	0.6072
2	2	9.5	contig00007_-_Protein_PilJ	1	1.4997	0.7349
4.02	4.02	29.4	contig00014_-_short_chain_dehydrogenase	3	1.4859	0.0976
2	2	8.8	contig00006_-_phosphoribosylaminoimidazole_synthetase	1	1.4723	0.4963
2.12	2.12	11.2	contig00004_-_antiporter_inner_membrane_protein	1	1.4588	0.4344
2.2	2.2	8.2	contig00004_-_aspartate_kinase	2	1.4588	0.541
4.01	4.01	14.4	contig00006_-_rbcR,_LysR_transcriptional_regulator	2	1.4588	0.6402
4	4	8	contig00012_-_GTP-binding_protein_YchF	4	1.4588	0.6769
6	6.01	12.1	contig00022_-_transketolase	5	1.4454	0.8021
2.01	2.01	7.3	contig00004_-_Dyp-type_peroxidase_family	1	1.4322	0.033
11.05	11.05	26.7	contig00006_-_rpsD,_30S_ribosomal_protein_S4	6	1.4322	0.9838
14	14	48.1	contig00016_-_oxidative_damage_protection_protein	16	1.4322	0.2907
3.47	3.56	12.7	contig00016_-_VacJ,_Surface_lipoprotein	2	1.4322	0.3379
2	2	8.7	contig00024_-_thiamine_biosynthesis_protein_ThiC	1	1.4322	0.5214
2	2	6.1	contig00002_-_3-phosphoshikimate_1-carboxyvinyltransferase	1	1.406	0.5389
26.61	26.61	29.8	contig00003_-_F0F1_ATP_synthase_subunit_beta	22	1.406	0.1279
13.15	13.15	36.5	contig00007_-_putative_outer_membrane_lipoprotein	11	1.3804	0.709
2.02	2.02	2.8	contig00003_-_c-di-GMP_phosphodiesterase_class_II	1	1.3677	0.5625
3.85	3.85	21.2	contig00005_-_Cell_division_protein_ZapD	2	1.3677	0.5639
6.01	6.01	17.3	contig00012_-_gatB,_aspartyl/glutamyl-tRNA_amidotransferase_subunit_B	3	1.3677	0.222
2.11	2.11	9.2	contig00020_-_Ferredoxin--NADP_reductase	1	1.3677	0.4048
2	2	7.9	contig00007_-_tRNA_threonylcarbamoyladenosine_biosynthesis_protein_RimN	1	1.3552	0.5663

7.6	7.6	15	contig00017_-_dihydrolipoamide_acetyltransferase	5	1.3552	0.1656
4	4	17.3	contig00002_-_DsrE/DsrF-like_family	3	1.3428	0.4937
4	4	39.6	contig00007_-_Sec-independent_protein_translocase_protein_TatA	2	1.3428	0.974
4.37	4.37	24.5	contig00010_-_nusB,_transcription_antitermination_protein_NusB	2	1.3428	0.3075
2.24	2.24	19.2	contig00004_-_Cytochrome_C_oxidase,_cbb3-type,_subunit_III	2	1.3305	0.6087
3.64	3.76	14.7	contig00017_-_dihydrolipoamide_dehydrogenase	2	1.3062	0.8517
7.89	7.89	37.2	contig00022_-_carboxysome_peptide_B	4	1.3062	0.4762
1.57	1.57	17.7	contig00026_-_Ferredoxin,_2Fe-2S	1	1.3062	0.6038
2	2	13.1	contig00007_-_glutamate_synthase_small_subunit_family_protein,_proteobacterial	1	1.2942	0.6329
6.01	6.01	43.6	contig00020_-_Bcp,_Peroxiredoxin	3	1.2942	0.5402
2	2	3.4	contig00003_-_argininosuccinate_lyase	1	1.2589	0.6992
5.35	5.35	8.6	contig00003_-_phosphoenolpyruvate_synthase	3	1.2589	0.2761
2	2	2.4	contig00004_-_Tetratricopeptide_TPR_4	1	1.2589	0.6485
4	4	30.1	contig00006_-_Putative_lipoprotein	2	1.2474	0.6262
14.13	14.13	56.7	contig00009_-_CspC,_Cold_shock_proteins	11	1.2474	0.3247
6.45	6.45	36.3	contig00030_-_rpsL,_30S_ribosomal_protein_S12	5	1.2474	0.4108
4	4	9.9	contig00007_-_Orotate_phosphoribosyltransferase	3	1.2359	0.5792
3.29	5.56	14.9	contig00001_-_clpA,_ATP-dependent_Clp_protease_ATP-binding_subunit	3	1.2246	0.2684
17.76	17.76	18.1	contig00001_-_polynucleotide_phosphorylase/polyadenylase	9	1.2246	0.5159
2	2	10.2	contig00006_-_Glycine-zipper_containing_OmpA-like_membrane_domain	2	1.2246	0.895
8.26	8.26	8.8	contig00006_-_Zn-finger_containing_NTP_pyrophosphohydrolase	4	1.2246	0.6155
2	2	4.8	contig00020_-_periplasmic_serine_protease,_Do/DeqQ_family	1	1.2246	0.6835
2.05	2.05	13.2	contig00006_-_Uncharacterized_conserved_secreted_or_membrane_protein	2	1.2134	0.6216
8.71	8.71	14.6	contig00025_-_5-methyltetrahydropteroyltriglutamate--_homocysteine_S-methyltransferase	6	1.1912	0.6083
4.82	4.82	14	contig00009_-_N-ethylmaleimide_reductase	3	1.1803	0.8615
2	2	8.1	contig00009_-_Rhodanese_Homology_Domain	1	1.1803	0.7733
2.04	2.04	11.3	contig00017_-_ABC_transporter,_phosphonate,_periplasmic_substrate-binding_protein	2	1.1803	0.6594
26.72	26.72	43.9	contig00003_-_phosphoribulokinase	20	1.1695	0.9485
5.77	5.8	43.9	contig00005_-_CopC_domain	6	1.1695	0.4929
2.02	2.02	13.9	contig00006_-_signal_recognition_particle_protein	1	1.1695	0.7384
2	2	11.3	contig00026_-_Trypsin-like_peptidase_domain	1	1.1695	0.8342
3	3	9.3	contig00002_-_Segregation_and_condensation_protein_B_homolog	2	1.1588	0.5655
2.79	2.79	50.8	contig00006_-_rpmD,_50S_ribosomal_protein_L30	3	1.1588	0.5864
3.89	3.89	11.7	contig00006_-_putative_GTP_cyclohydrolase	2	1.1376	0.3408
2.1	2.1	11.6	contig00012_-_prfA,_peptide_chain_release_factor_1	1	1.1376	0.7325
9.5	9.58	21.1	contig00011_-_HflC_protein	5	1.1272	0.1343
2	2	11.3	contig00010_-_Oxidoreductase,_aldo/keto_reductase_family	1	1.1169	0.2451
2.03	2.03	13.6	contig00001_-_General_stress_protein_69	1	1.1066	0.8237
2.05	2.05	12.5	contig00010_-_recombination_and_DNA_strand_exchange_inhibitor_protein	1	1.1066	0.5367

3.2	3.2	47.3	contig00015_-_50S_ribosomal_protein_L33	3	1.1066	0.9367
2	2	5.2	contig00002_-_N-ethylammeline_chlorohydrolase	1	1.0965	0.9287
7.07	7.07	24.1	contig00004_-_ferredoxin-NADP_reductase	4	1.0965	0.6027
2	2.01	7.9	contig00011_-_tryptophan_synthase_subunit_beta	1	1.0765	0.6903
28.36	28.36	16.1	contig00030_-_DNA-directed_RNA_polymerase_subunit_beta	16	1.0765	0.4303
4.9	4.93	29.9	contig00004_-_Putative_lipoprotein	3	1.0666	0.7322
2	2	3.5	contig00022_-_Probable_transcriptional_regulator_LumQ	1	1.0666	0.8803
4	4	19.1	contig00017_-_minE,_cell_division_topological_specificity_factor_MinE	2	1.0568	0.9173
1.52	1.52	1.7	contig00024_-_bifunctional_heptose_7-phosphate_kinase/heptose_1-phosphate_adenyltransferase	1	1.0568	0.9423
2	2	10.4	contig00002_-_Predicted_kinase	1	1.0471	0.9121
5.96	6	11.8	contig00013_-_Putative_methyl-accepting_chemotaxis_AlkN	3	1.0471	0.8243
12	12	27.3	contig00030_-_50S_ribosomal_protein_L11	6	1.0471	0.7192
10.09	10.09	32.6	contig00001_-_rpsB,_30S_ribosomal_protein_S2	9	1.028	0.9683
2	2	7.6	contig00003_-_Bacterial_protein_of_unknown_function_(DUF945)	1	1.028	0.9436
5.35	5.35	9.1	contig00003_-_putative_quinone_oxidoreductase,_YhdH/YhfP_family	3	1.028	0.9393
2	2	8.3	contig00009_-_glycine_cleavage_system_protein_H	2	1.028	0.9635
3.81	3.81	15.8	contig00006_-_tldD,_protease_TldD	3	1.0186	0.8431
3.62	3.63	14.8	contig00007_-_RNA_polymerase_sigma_factor_RpoD	2	1.0186	0.1993
2	2	6.5	contig00022_-_RuBisCO_operon_transcriptional_regulator	1	1.0186	0.9583
4	4.29	36.9	contig00026_-_rpsI,_30S_ribosomal_protein_S9	3	1.0186	0.9716
2	2	3	contig00002_-_cmk,_cytidylate_kinase	1	1.0093	0.9747
2	2	9.1	contig00002_-_Protein_YciI	1	1.0093	0.9988
2	2.01	12.8	contig00003_-_helix_turn_helix,_Arsenical_Resistance_Operon_Repressor	1	1.0093	0.9658
3.41	3.41	14.8	contig00013_-_acetyl-CoA_carboxylase_carboxyltransferase_subunit_alpha	2	1.0093	0.8505
6.88	6.88	20.9	contig00026_-_ribulose-phosphate_3-epimerase	4	1.0093	0.615
2	2	5.5	contig00004_-_LPS-assembly_protein_LptD	1	1	0.3486
2.26	4.2	9.3	contig00012_-_efflux_transporter,_outer_membrane_factor_(OMF)_lipoprotein,_NodT_family	4	1	0.9818
2	2	6.9	contig00015_-_purH,_bifunctional_phosphoribosylaminoimidazolecarboxamide_formyltransferase/IMP_cyclohydrolase	1	1	0.9899
6	6	12.2	contig00004_-_HdrC,_Heterodisulfide_reductase,_subunit_C	4	0.9908	0.8576
4.01	4.01	24.4	contig00004_-_Penicillin-binding_protein_5	2	0.9908	0.9957
7.62	7.62	12.2	contig00005_-_CirA,_Outer_membrane_receptor_proteins,_mostly_Fe_transport	5	0.9908	0.9055
6	6	10.5	contig00007_-_1-(5-phosphoribosyl)-5-[(5-_phosphoribosylamino)methylideneamino]_imidazole-4-carboxamide_isomerase	3	0.9908	0.9214
6.01	6.01	23.9	contig00011_-_FtsH_protease_regulator_HflC	5	0.9908	0.8947
4.74	4.74	11.6	contig00002_-_Translation_initiation_factor_IF-2	3	0.9817	0.7713
4.28	4.28	50.6	contig00003_-_rpmA,_50S_ribosomal_protein_L27	3	0.9817	0.9759
13.6	15.69	22	contig00004_-_DacC,_D-alanyl-D-alanine_carboxypeptidase	9	0.9817	0.7786
2	2	3.4	contig00027_-_Tas,_Predicted_oxidoreductases	1	0.9817	0.9885
2	2	19.3	contig00004_-_PspE,_Rhodanese-related_sulfurtransferase	1	0.9727	0.9545

4	4	37.2	contig00030_-_Elongation_factor_G_C-terminus	3	0.9727	0.7588
22.03	22.03	27.1	contig00003_-_Prc,_Periplasmic_protease	14	0.9638	0.9508
2.01	2.01	8.7	contig00013_-_hscB,_co-chaperone_HscB	1	0.9638	0.7899
6.84	6.84	16.9	contig00003_-_pntA,_NAD(P)_transhydrogenase_subunit_alpha	4	0.955	0.7245
4	4	8.6	contig00009_-_glycine_dehydrogenase_subunit_1	4	0.955	0.4603
31.82	31.82	52.7	contig00044_-_elongation_factor_Tu	33	0.955	0.7138
2	2	6.7	contig00010_-_NADP-dependent_L-serine/L-allo-threonine_dehydrogenase_YdfG	1	0.9376	0.9101
2	2	4.9	contig00012_-_Uncharacterized_Fe-S_protein	1	0.9376	0.9157
4.01	4.03	12.2	contig00001_-_fabG,_3-ketoacyl-(acyl-carrier-protein)_reductase	2	0.929	0.8428
6	6.01	23.6	contig00003_-_OmpR,_Response_regulators_consisting_of_a_CheY-like_receiver_domain_and_a_DNA-binding_domain	3	0.929	0.4927
2	2	18	contig00001_-_Acyl_carrier_protein	1	0.912	0.8335
4	4	24.2	contig00002_-_ihfB,_integration_host_factor_subunit_beta	2	0.912	0.7671
2.83	2.83	13.6	contig00007_-_pyrroline-5-carboxylate_reductase	2	0.912	0.7155
2	2	10.2	contig00007_-_Hemerythrin_HHE_cation_binding_domain	2	0.9036	0.5415
10.34	10.34	67.4	contig00016_-_DNA-directed_RNA_polymerase_subunit_omega	8	0.9036	0.953
13.19	13.19	34.1	contig00006_-_rplC,_50S_ribosomal_protein_L3	8	0.8954	0.8515
3.54	3.58	3.9	contig00007_-_phosphoglyceromutase	2	0.8954	0.7982
4	4	5.4	contig00003_-_hemC,_porphobilinogen_deaminase	2	0.8872	0.93
9.75	9.75	43.2	contig00006_-_rpsH,_30S_ribosomal_protein_S8	8	0.8872	0.334
6	6.01	42.2	contig00006_-_rpsS,_30S_ribosomal_protein_S19	6	0.8872	0.7732
2	2	3.6	contig00014_-_Hydrogen_peroxide-inducible_genes_activator	1	0.8872	0.9678
7.48	7.48	25.5	contig00018_-_Gram-negative_porin	7	0.8872	0.7029
6.08	6.18	17	contig00008_-_AfuA,_ABC-type_Fe3+_transport_system,_periplasmic_component	6	0.879	0.6175
2	2	19	contig00012_-_Aspartyl/glutamyl-tRNA(Asn/Gln)_amidotransferase_subunit_C	1	0.879	0.6771
2	2	5.8	contig00004_-_Enolase-phosphatase_E1	1	0.871	0.795
2.16	2.16	26.8	contig00014_-_Tetratricopeptide_TPR_1_repeat-containing_protein	1	0.871	0.1287
1.48	1.48	6.8	contig00017_-_Carboxymuconolactone_decarboxylase	1	0.871	0.8042
11.18	11.43	16	contig00005_-_ResB-like_family	9	0.863	0.7136
4.01	4.01	14.5	contig00010_-_Nucleoprotein/polynucleotide-associated_enzyme	2	0.863	0.9158
7.38	7.38	13.7	contig00002_-_acetyl-CoA_synthetase	4	0.8551	0.4757
1.6	1.6	9.7	contig00003_-_branched-chain_amino_acid_aminotransferase	1	0.8551	0.7687
2	2	7.6	contig00011_-_chorismate_synthase	1	0.8551	0.7534
4	4	24.7	contig00026_-_rplM,_50S_ribosomal_protein_L13	3	0.8551	0.7134
14.98	14.98	67.1	contig00016_-_SirA-like_protein	14	0.8472	0.9833
2.25	2.26	17.7	contig00021_-_hypothetical_protein	1	0.8472	0.8775
2	2	10.1	contig00003_-_membrane_protein_insertase	1	0.8395	0.7469
4	4	19	contig00005_-_UspA_domain_protein	3	0.8395	0.765
2	2	6.4	contig00006_-_Chaperone_protein_HtpG	1	0.8395	0.7467
2	2	28.2	contig00007_-_30S_ribosomal_protein_S21	1	0.8395	0.3994

2	2.01	8.4	contig00009_-_histidinol-phosphatase,_inositol_monophosphatase_family	1	0.8395	0.8439
2	2.01	15.1	contig00011_-_Vi_polysaccharide_biosynthesis_protein_TviB	1	0.8395	0.6119
4	4	25.8	contig00022_-_carboxysome_peptide_A	5	0.8395	0.7812
27.76	27.76	50.8	contig00003_-_ATP_synthase_subunit_alpha	20	0.8318	0.3718
8.04	8.04	18.4	contig00006_-_RND_family_efflux_transporter,_MFP_subunit	5	0.8318	0.2339
23.27	23.27	37.9	contig00002_-_Trigger_factor	25	0.8241	0.4755
2	2.01	10.8	contig00002_-_Carbamoyl-phosphate_synthase_small_chain,_CPSase_domain	1	0.8166	0.7414
10	10	24.2	contig00030_-_rplA,_50S_ribosomal_protein_L1	6	0.8166	0.5015
6	6.59	33.7	contig00002_-_SirA-like_protein	3	0.8091	0.1898
4	4	29	contig00002_-_Universal_stress_protein_MJ0531	2	0.8091	0.7106
2.05	2.05	4.7	contig00005_-_aspS,_aspartyl-tRNA_synthetase	1	0.8091	0.3111
3.62	3.62	16.2	contig00026_-_Cytochrome_c553	2	0.8091	0.5971
2	2	16.3	contig00001_-_lipoprotein_releasing_system,_ATP-binding_protein	1	0.8017	0.5506
4.22	4.22	11.5	contig00002_-_pyridoxine_5'-phosphate_synthase	2	0.8017	0.4987
3.28	3.28	14.4	contig00016_-_dapF,_diaminopimelate_epimerase	2	0.8017	0.252
2.44	2.45	4.8	contig00005_-_Cellulose_synthase_operon_protein_C_C-terminus	2	0.7943	0.6573
2	2	24.3	contig00036_-_Cysteine_desulfhydrase_/_Selenocysteine_lyase	1	0.7943	0.6681
3.8	3.8	14.8	contig00002_-_Pterin_4_alpha_carbinolamine_dehydratase	2	0.787	0.584
8.11	8.11	27.6	contig00004_-_transaldolase	6	0.787	0.2601
2.36	2.36	6.3	contig00022_-_S-adenosylmethionine_synthetase	1	0.787	0.6243
2.06	2.06	5.5	contig00001_-_NMT1-like_family	1	0.7798	0.6549
10	10	16.3	contig00003_-_Probable_chromosome-partitioning_protein_ParB	6	0.7798	0.0232
4.53	4.63	12.6	contig00006_-_Lpd,_Pyruvate/2-oxoglutarate_dehydrogenase_complex,_dihydrolipoamide_dehydrogenase_(E3)_component	2	0.7727	0.4678
2	2.01	9.8	contig00006_-_prolyl-tRNA_synthetase	1	0.7727	0.167
6	6	14.5	contig00002_-_tpiA,_triosephosphate_isomerase	3	0.7656	0.3492
6.03	6.03	14.2	contig00008_-_Outer_membrane_lipoprotein_Slp_family	4	0.7656	0.5425
2	2	4.7	contig00003_-_6-phosphofructokinase	1	0.7516	0.6101
6	6	6	contig00009_-_Protein_HelA	3	0.7516	0.4592
1.68	1.68	13.5	contig00014_-_ilvH,_acetolactate_synthase_3_regulatory_subunit	1	0.7516	0.6714
2	2	1.6	contig00016_-_dihydrolipoamide_succinyltransferase	1	0.7516	0.6072
1.74	2	9.2	contig00012_-_gatA,_aspartyl/glutamyl-tRNA_amidotransferase_subunit_A	1	0.7447	0.6164
1.41	1.41	12.9	contig00003_-_GTPase_obg	1	0.7311	0.581
25.61	25.61	33.7	contig00004_-_mdoG,_glucan_biosynthesis_protein_G	16	0.7311	0.2405
4	4	6.4	contig00003_-_ileS,_isoleucyl-tRNA_synthetase	2	0.7244	0.0794
3.48	3.48	5	contig00022_-_CheA,_Chemotaxis_protein_histidine_kinase_and_related_kinases	2	0.7244	0.574
2	2	11.4	contig00007_-_3'(2'),5'-bisphosphate_nucleotidase,_bacterial	1	0.7178	0.7374
2.01	2.01	11.4	contig00006_-_GuaA,_GMP_synthase_-_Glutamine_amidotransferase_domain	2	0.7047	0.6824
2	2.01	4.3	contig00007_-_Voltage_gated_chloride_channel	1	0.7047	0.5512

2.26	2.26	14.4	contig00022_-_ATP-dependent_dethiobiotin_synthetase_BioD	2	0.7047	0.5675
2.01	2.01	12	contig00026_-_Bacterial_microcompartments_are_primitive_organelles_composed_entirely_of_protein_subunits	2	0.7047	0.6501
2	2	6.3	contig00007_-_Cyclic_nucleotide-binding_domain	1	0.6982	0.5369
5.31	5.31	41.3	contig00009_-_molybdopterin_biosynthesis_protein_MoeB	5	0.6982	0.5468
4	4	11.1	contig00005_-_Putative_NADH_dehydrogenase/NAD(P)H_nitroreductase_AF_0226	2	0.6918	0.3999
6	6	17.8	contig00012_-_Cytochrome_C	3	0.6918	0.5741
15.27	15.27	12.5	contig00001_-_Phosphoesterase_family	9	0.6855	0.2039
2.47	2.47	9.6	contig00007_-_Hpt_domain	2	0.6855	0.309
5.73	5.73	22.3	contig00004_-_inorganic_pyrophosphatase	3	0.6792	0.9501
13.32	13.32	22.3	contig00004_-_Multidrug_resistance_outer_membrane_protein_MdtP	7	0.6792	0.2468
2	2	19.8	contig00007_-_hisE,_phosphoribosyl-ATP_pyrophosphatase	1	0.6792	0.5121
2	2	7.5	contig00004_-_Ferredoxin_1	1	0.673	0.5655
2	2	7.2	contig00005_-_Predicted_peroxiredoxins	1	0.673	0.6144
23.91	23.91	36.8	contig00008_-_Carbohydrate-selective_porin,_OprB_family	19	0.673	0.1404
2	2	11.4	contig00013_-_Histidine--tRNA_ligase	1	0.673	0.5251
25.88	25.88	39	contig00003_-_glnA,_glutamine_synthetase	21	0.6607	0.0591
4	4	26	contig00011_-_Predicted_peroxiredoxins	10	0.6607	0.7705
4.8	4.8	42.7	contig00026_-_Putative_pterin-4_alpha-carbinolamine_dehydratase-like_protein	5	0.6607	0.6868
5.82	5.82	19.8	contig00005_-_RbsK,_Sugar_kinases,_ribokinase_family	3	0.6486	0.3738
1.57	1.57	8.3	contig00006_-_RfbB,_dTDP-D-glucose_4,6-dehydratase	1	0.6486	0.4728
2	2	11.3	contig00007_-_ATPase_components_of_ABC_transporters_with_duplicated_ATPase_domains	1	0.6486	0.4716
13.6	13.6	23.4	contig00007_-_LysM_domain/BON_superfamily_protein	7	0.6486	0.1793
2.04	2.04	7.2	contig00001_-_fructose-bisphosphate_aldolase	2	0.6427	0.5423
4.01	4.01	7.4	contig00001_-_periplasmic_serine_protease,_Do/DeqQ_family	2	0.6427	0.5822
2	2	6.4	contig00005_-_Type_4_fimbrial_assembly_protein_PilC	1	0.6427	0.4697
2.02	2.02	8.4	contig00018_-_Protein_RcaC	1	0.6427	0.5405
3.92	4	34.4	contig00001_-_Trm112p-like_protein	2	0.6368	0.5043
2	2	4.4	contig00011_-_Peptidase_C26	1	0.6368	0.4631
2	2	13.3	contig00004_-_YdcF-like_protein	1	0.631	0.4551
2	2	13.8	contig00009_-_Phosphoesterase_family	1	0.6252	0.4365
8.53	8.53	25.4	contig00016_-_SoxAX_cytochrome_complex_subunit_A	5	0.6252	0.1843
4	4	43.2	contig00006_-_50S_ribosomal_protein_L15	2	0.6194	0.4393
6.06	6.06	23	contig00012_-_YecA_family_protein	5	0.6194	0.338
1.92	2	11.4	contig00002_-_Scavenger_mRNA_decapping_enzyme_C-term_binding	1	0.6138	0.5013
2	2	17.7	contig00011_-_Ttg2C,_ABC-type_transport_system_involved_in_resistance_to_organic_solvents	1	0.6138	0.4348
2	2	6.9	contig00012_-_GntR,_Transcriptional_regulators	1	0.6081	0.4318
2.08	2.08	7.4	contig00005_-_UDP-N-acetylmuramoyl-tripeptide--D-alanyl-D-_alanine_ligase	2	0.597	0.3504
11.45	11.45	21.8	contig00010_-_glyA,_serine_hydroxymethyltransferase	7	0.597	0.2424
15.33	15.33	57.7	contig00030_-_30S_ribosomal_protein_S7	13	0.597	0.1807

2.01	4.03	10.5	contig00010_-_Hemolysin_secretion_protein	3	0.5861	0.4093
2	2	4.6	contig00001_-_3-oxoacyl-(acyl_carrier_protein)_synthase_III	1	0.5808	0.4042
2.14	2.15	5.5	contig00006_-_AcrB,_Cation/multidrug_efflux_pump	1	0.5808	0.4029
2.01	2.03	11.3	contig00006_-_heat_shock_protein_90	1	0.5808	0.5073
3.1	3.1	16.3	contig00001_-_PepB,_Leucyl_aminopeptidase	2	0.5754	0.3029
2	2	5.7	contig00002_-_clpP,_ATP-dependent_Clp_protease_proteolytic_subunit	1	0.5702	0.5879
4	4	16.6	contig00012_-_EVE_domain	2	0.5702	0.3007
2.04	2.04	27.4	contig00001_-_50S_ribosomal_protein_L32	1	0.5649	0.3886
4	4	19	contig00005_-_ManX,_Phosphotransferase_system,_mannose/fructose-specific_component_IIA	2	0.5649	0.1365
4	4	6.2	contig00016_-_periplasmic_serine_protease,_Do/DeqQ_family	2	0.5649	0.9344
2	2	21.7	contig00002_-_Protein-export_membrane_protein_SecG	1	0.5598	0.6187
2	2.03	7	contig00002_-_Predicted_3'-5'_exonuclease_related_to_the_exonuclease_domain_of_PolB	1	0.5546	0.3767
2	2	18.4	contig00016_-_PAS_sensor_protein	1	0.5546	0.3613
36.97	37.02	30.4	contig00016_-_Protein_AsmA	25	0.5546	0.0233
47.72	47.72	73.3	contig00004_-_outer_membrane_protein_A	44	0.5495	0.0108
2	2	4	contig00005_-_Probable_3-mercaptopyruvate_sulfurtransferase	1	0.5495	0.3723
2	2	5.3	contig00009_-_NAD(P)H:quinone_oxidoreductase	1	0.5495	0.3312
2	2	12.3	contig00017_-_Thioredoxin_domain-containing_protein	1	0.5495	0.3718
4.01	4.01	23.9	contig00006_-_50S_ribosomal_protein_L18	3	0.5395	0.5477
2	2	9	contig00001_-_tmk,_thymidylate_kinase	1	0.52	0.349
6	6	41.2	contig00006_-_rplV,_50S_ribosomal_protein_L22	5	0.52	0.2523
4	4	33.7	contig00006_-_rpsP,_30S_ribosomal_protein_S16	2	0.52	0.4575
9.91	9.91	20.9	contig00012_-_RND_family_efflux_transporter,_MFP_subunit	8	0.52	0.5236
2	2	24.2	contig00002_-_Signal_peptidase_I	2	0.5152	0.3338
19.75	21.77	29.9	contig00013_-_eno,_enolase	15	0.5152	0.1903
4.12	4.12	11.6	contig00001_-_TolC,_Outer_membrane_protein	2	0.5105	0.3111
1.68	1.68	6.3	contig00013_-_lysS,_lysyl-tRNA_synthetase	1	0.5105	0.3265
2	2	9.9	contig00022_-_S-adenosyl-L-homocysteine_hydrolase	1	0.5105	0.3977
2.31	2.32	17.9	contig00001_-_hypoxanthine-guanine_phosphoribosyltransferase	1	0.5058	0.5067
2	4	4.8	contig00005_-_Transcriptional_regulator	2	0.4966	0.3266
2.01	2.01	13.7	contig00007_-_bifunctional_pyrimidine_regulatory_protein_PyrR_uracil_phosphoribosyltransferase	1	0.492	0.3333
2.02	2.02	4.9	contig00010_-_glutamate_decarboxylase	1	0.4786	0.3131
2	2	8.6	contig00001_-_isocitrate_dehydrogenase	1	0.4742	0.3117
4	4	11.9	contig00009_-_glycine_dehydrogenase_subunit_2	2	0.4742	0.2366
16.62	16.62	29.6	contig00022_-_Glyceraldehyde-3-phosphate_dehydrogenase	11	0.4742	0.2353
2	2	7.2	contig00004_-_metG,_methionyl-tRNA_synthetase	1	0.4656	0.304
5.78	5.78	4.5	contig00003_-_TonB-dependent_siderophore_receptor	3	0.4571	0.974
6	6.09	12.7	contig00017_-_aceE,_pyruvate_dehydrogenase_subunit_E1	3	0.4571	0.2034
1.82	2	14.3	contig00002_-_PilZ_domain	1	0.4529	0.2941

2.03	2.03	4.8	contig00006_-_purT,_phosphoribosylglycinamide_formyltransferase_2	1	0.4487	0.2676
4.02	4.02	14.7	contig00035_-_TolQ,_Biopolymer_transport_proteins	2	0.4406	0.8242
4.37	5.07	25.2	contig00011_-_leuD,_isopropylmalate_isomerase_small_subunit	3	0.4365	0.0084
2.03	2.03	12.2	contig00017_-_PleD,_Response_regulator_containing_a_CheY-like_receiver_domain_and_a_GGDEF_domain	2	0.4365	0.2362
2.01	2.01	13.9	contig00022_-_N5-carboxyaminoimidazole_ribonucleotide_mutase	1	0.4365	0.2676
3.92	4	7.2	contig00003_-_dihydroorotate_dehydrogenase_1B	2	0.4325	0.1455
2	2.03	5.3	contig00005_-_LppC_putative_lipoprotein	2	0.4325	0.2817
2	2	14.5	contig00005_-_Bacterial_cellulose_synthase_subunit	1	0.4246	0.2746
4	4	5.4	contig00005_-_glutamate-1-semialdehyde_aminotransferase	2	0.4169	0.5192
2	2	6.4	contig00026_-_Putative_transmembrane_protein_coupled_to_NADH-ubiquinone_oxidoreductase_chain_5-like_protein	1	0.413	0.2683
3.78	3.78	40.7	contig00010_-_Putative_cytoplasmic_protein	2	0.4093	0.2648
2.29	2.29	16.2	contig00008_-_SCO1_protein_homolog	2	0.4055	0.2654
2	2	6.1	contig00010_-_phosphoenolpyruvate_carboxylase	1	0.4055	0.3673
2.04	2.04	3.7	contig00001_-_phosphoribosylformylglycinamidine_synthase	1	0.4018	0.3815
2	2	10.4	contig00005_-_TolQ_protein	1	0.4018	0.4221
2.55	2.55	15.2	contig00010_-_bifunctional_3,4-dihydroxy-2-butanone_4-phosphate_synthase/GTP_cyclohydrolase_II-like_protein	2	0.4018	0.253
3.75	3.75	14.3	contig00003_-_Glutaredoxin,_GrxC_family	2	0.3981	0.8829
3.03	3.03	21.1	contig00006_-_rplS,_50S_ribosomal_protein_L19	3	0.3945	0.3748
4	4	9.9	contig00008_-_Hemerythrin_HHE_cation_binding_domain	2	0.3945	0.5108
3.39	3.39	46.9	contig00008_-_Membrane_fusogenic_activity	5	0.3945	0.2616
2	2	28.1	contig00024_-_Predicted_transcriptional_regulator	1	0.3837	0.2503
3.59	3.59	25.9	contig00004_-_Phospholipid-binding_protein	2	0.3802	0.4239
10.07	10.07	61.2	contig00006_-_rplW,_50S_ribosomal_protein_L23	6	0.3767	0.0192
4	4	27.7	contig00007_-_Pilus_assembly_protein,_PilO	2	0.3767	0.2828
2	2	5.2	contig00017_-_Chemotaxis_protein_CheY	1	0.3597	0.2377
4.02	4.02	9.2	contig00002_-_DNA_gyrase_subunit_A	2	0.3565	0.2263
8.14	8.14	32.6	contig00009_-_tpx,_lipid_hydroperoxide_peroxidase	7	0.3565	0.4099
2	2	12.2	contig00015_-_WcaG,_Nucleoside-diphosphate-sugar_epimerases	1	0.3565	0.2335
14.28	14.28	11.2	contig00001_-_aconitate_hydratase	8	0.3467	0.7702
1.92	2	4.4	contig00002_-_PTC1,_Serine/threonine_protein_phosphatase	1	0.3467	0.2278
2.02	2.03	39.7	contig00006_-_50S_ribosomal_protein_L17	1	0.3467	0.2281
8.01	8.01	19.9	contig00001_-_FabD,_(acyl-carrier-protein)_S-malonyltransferase	6	0.3436	0.1023
2.62	2.62	16.5	contig00012_-_ribose-5-phosphate_isomerase_A	2	0.3404	0.2472
2	2	5.3	contig00002_-_Uncharacterized_oxidoreductase_YfjR	1	0.3311	0.3355
12.33	12.33	22.1	contig00004_-_HdrB,_Heterodisulfide_reductase,_subunit_B	12	0.3221	0.0437
10.02	10.02	21.7	contig00016_-_multidrug_efflux_system_protein_EmrA	7	0.3221	0.0434
2	2	12	contig00001_-_pyrH,_uridylate_kinase	1	0.3192	0.2115
2	2.01	5.7	contig00011_-_Protein_CapI	1	0.3133	0.2285
2.01	2.02	4.7	contig00012_-_multidrug_efflux_protein	1	0.3133	0.2088

4	4	2.8	contig00026_-_anthranilate_synthase_component_I	2	0.3105	0.3634
6.03	6.03	13.8	contig00002_-_Aminoglycoside_phosphotransferase	3	0.3076	0.1762
4.01	4.01	8.4	contig00007_-_Phosphomannomutase/phosphoglucomutase	2	0.302	0.4651
8	8	23.6	contig00006_-_30S_ribosomal_protein_S14	4	0.2992	0.292
6.13	6.13	20.6	contig00006_-_Fe-S_oxidoreductase-like_protein	3	0.2965	0.1683
6.01	6.01	22.9	contig00010_-_The_CBS_domain,_ is_a_small_domain_identified_in_cystathionine_β-synthase	4	0.2965	0.192
12	12	29.3	contig00017_-_transcriptional_regulator_PhoU	8	0.2938	0.1765
4.06	4.06	13.7	contig00003_-_Sporulation_related_domain	3	0.2831	0.2778
6.95	6.95	39.1	contig00006_-_rpsC,_30S_ribosomal_protein_S3	5	0.2831	0.2508
14	14	39.3	contig00001_-_Translation_initiation_factor_IF-3	8	0.278	0.0022
4	4	11.3	contig00005_-_succinyl-CoA_synthetase_subunit_alpha	3	0.278	0.2096
4.01	4.01	20.3	contig00011_-_Putative_signal_peptide_protein	4	0.2729	0.1848
4	4	15.7	contig00025_-_DsrE/DsrF/DrsH-like_family	2	0.2704	0.1957
8.01	8.03	7.5	contig00005_-_Multidrug_resistance_protein_MdtB	4	0.2535	0.0916
24.06	24.06	58.4	contig00006_-_rplB,_50S_ribosomal_protein_L2	15	0.2443	0.0238
2	3.59	3.9	contig00001_-_aspartate_aminotransferase	2	0.2377	0.1702
36.27	36.27	41.2	contig00022_-_fructose-1,6-bisphosphate_aldolase	29	0.2377	0
8.12	8.12	27.7	contig00001_-_Predicted_peroxiredoxins	7	0.2355	0.3303
2	2	4.7	contig00002_-_Ureidoglycolate_lyase	1	0.227	0.2458
7.03	9.31	17.8	contig00003_-_DNA_polymerase_III_subunit_beta	6	0.2228	0.1372
8	8	18.5	contig00005_-_outer_membrane_assembly_lipoprotein_YfiO	4	0.2188	0.6094
11.64	11.64	47.3	contig00006_-_rplE,_50S_ribosomal_protein_L5	7	0.2109	0.0309
14.68	14.68	33.1	contig00014_-_ketol-acid_reductoisomerase	11	0.2089	0.2362
5.89	6.02	25.6	contig00006_-_DsrE/DsrF-like_family	4	0.207	0.1309
15.27	15.27	15.6	contig00021_-_Protein_translocase_subunit_SecD	8	0.2051	0.0034
23.78	24.31	40.4	contig00006_-_DNA-directed_RNA_polymerase_subunit_alpha	17	0.1977	0
12.11	12.11	41.1	contig00004_-_Acireductone_dioxygenase	7	0.1888	0.0038
11.73	11.73	42.6	contig00006_-_rpsE,_30S_ribosomal_protein_S5	8	0.1837	0.0114
4	4	1.9	contig00008_-_cytochrome_c_oxidase,_subunit_I	2	0.1837	0.283
2	2	12.2	contig00011_-_trpA,_tryptophan_synthase_subunit_alpha	1	0.182	0.1443
2	2	6.3	contig00006_-_PhoH,_Phosphate_starvation-inducible_protein_PhoH,_predicted_ATPase	1	0.1754	0.1771
2.76	2.81	12.5	contig00008_-_Tas,_Predicted_oxidoreductases	2	0.1738	0.141
4.76	4.76	19.7	contig00009_-_gabD1,_succinic_semialdehyde_dehydrogenase	3	0.1722	0.1236
8	10	15.1	contig00012_-_HDOD_domain	5	0.1722	0.0275
4.36	4.37	23.1	contig00008_-_Uncharacterized_isochorismatase_family_protein_YwoC	3	0.1706	0.0645
6.24	6.24	18.3	contig00003_-_LivK,_ABC-type_branched-chain_amino_acid_transport_systems,_periplasmic_component	4	0.169	0.0218
4	4	7.7	contig00003_-_glyQ,_glycyl-tRNA_synthetase_subunit_alpha	2	0.166	0.2318
2.38	2.38	13.6	contig00015_-_Domain_of_unknown_function_(DUF4340)	1	0.166	0.2114
2	2	6.5	contig00022_-_Carboxysome_Shell_Carbonic_Anhydrase	1	0.166	0.2054

8	8	18	contig00009_-_gcvT,_glycine_cleavage_system_aminomethyltransferase_T	4	0.1629	0.0166
2	2	9.8	contig00009_-_Predicted_proteasome-type_protease	2	0.16	0.3329
4.08	4.08	19.8	contig00002_-_Cupin_domain	2	0.1514	0.1857
2	2	10.2	contig00002_-_FabI,_Enoyl-[acyl-carrier-protein]	1	0.1472	0.1292
8.34	8.34	17.4	contig00004_-_recA,_recombinase_A	6	0.1343	0.0418
9.28	9.28	41.5	contig00006_-_30S_ribosomal_protein_S11	8	0.1306	0.6065
10.73	10.73	20.1	contig00002_-_GalU,_UDP-glucose_pyrophosphorylase	5	0.1282	0.0266
14	14.03	23.5	contig00022_-_Phosphoglycerate_kinase	9	0.1247	0.3697
10.02	10.02	46.6	contig00006_-_rpsM,_30S_ribosomal_protein_S13	6	0.1236	0.0063
6.22	6.22	27.8	contig00005_-_phosphoheptose_isomerase	7	0.1225	0.0359
12.69	12.69	22.8	contig00003_-_fructose-1,6-bisphosphatase	9	0.118	0.0716
9.13	9.13	54.1	contig00014_-_LysR,_Transcriptional_regulator	6	0.118	0.0133
2	2	17.7	contig00010_-_ribH,_6,7-dimethyl-8-ribityllumazine_synthase	1	0.1159	0.1816
4	4.02	12.4	contig00009_-_Prc,_Periplasmic_protease	2	0.1148	0.1162
14.63	14.71	38.3	contig00030_-_50S_ribosomal_protein_L10	9	0.1107	0.0073
2	2	10.9	contig00003_-_Sulfide:quinone_oxidoreductase,_mitochondrial	1	0.1047	0.11
2	2	6.1	contig00008_-_Transcriptional_regulator_containing_an_amidase_domain_and_an_AraC-type_DNA-binding_domain	1	0.1038	0.1094
2	2	3.4	contig00002_-_NADPH_dehydrogenase_NamA	1	0.0991	0.3217
4	4	6.1	contig00002_-_mltD,_membrane-bound_lytic_murein_transglycosylase_D	3	0.0929	0.1043
11.75	11.75	14.9	contig00012_-_hypothetical_protein	6	0.0929	0.0496
2	2	7.6	contig00004_-_fabG,_3-ketoacyl-(acyl-carrier-protein)_reductase	1	0.092	0.1041
13.44	14.82	37.2	contig00001_-_RND_family_efflux_transporter,_MFP_subunit	11	0.0912	0.0353
6	6.01	9.3	contig00003_-_Tas,_Predicted_oxidoreductases	3	0.0863	0.0371
4.03	4.03	13.8	contig00005_-_Cation_efflux_system_protein_CzcB	2	0.0863	0.0311
2	2	11.6	contig00002_-_SseA,_Rhodanese-related_sulfurtransferase	1	0.0809	0.1652
2	2	6.5	contig00001_-_ferrochelatase	1	0.0802	0.1486
2	2	3.5	contig00006_-_secY,_preprotein_translocase_subunit_SecY	1	0.078	0.1595
9.45	9.45	52.8	contig00022_-_Ribulose_bisphosphate_carboxylase_small_chain	12	0.0724	0.0289
16.77	17.73	20.7	contig00017_-_UshA,_5'-nucleotidase/2',3'-cyclic_phosphodiesterase_and_related_esterases	14	0.0679	0
21.96	21.96	73.3	contig00066_-_Major_carboxysome_shell_protein_1A	38	0.0649	0.0147
8.4	8.4	15.5	contig00008_-_signal_peptide_peptidase_SppA,_67K_type	4	0.0625	0.0177
43.59	43.59	39.8	contig00022_-_Carboxysome_shell_peptide_mid-region	42	0.0586	0.1271
6	6	21.4	contig00004_-_Glucosamine-6-phosphate_isomerases/6-phosphogluconolactonase	4	0.0535	0.0142
3.26	3.26	8.4	contig00001_-_Phosphoketolase	3	0.0515	0.1478
2	2	2.2	contig00008_-_Periplasmic_binding_protein	1	0.0506	0.0959
14.88	14.88	22.1	contig00002_-_periplasmic_folding_chaperone	8	0.0483	0.0018
4.01	4.01	20.9	contig00009_-_Membrane_Fusion_Protein_cluster_2	3	0.0449	0.0828
2	2	7.8	contig00012_-_GIY-YIG_catalytic_domain	1	0.0394	0.2057
8.03	8.03	25.9	contig00008_-_SCO1/SenC_protein	4	0.0384	0.0004

4	4	15.1	contig00006_-_Proline-rich_region	2	0.0363	0.1614
10.49	10.49	12.3	contig00001_-_MrcA,_Membrane_carboxypeptidase/penicillin-binding_protein	8	0.0313	0.0082
2.32	2.32	5.9	contig00012_-_TPR_repeat-containing_protein_PA4667	1	0.0305	0.1694
2	2	32.8	contig00005_-_3-deoxy-D-manno-octulosonate_8-phosphate_phosphatase_KdsC	1	0.0302	0.0711
12	12	37.2	contig00005_-_Putative_peptidoglycan_binding_domain	8	0.0299	0.0002
2	2	12.8	contig00005_-_sucC,_succinyl-CoA_synthetase_subunit_beta	1	0.0286	0.1501
2	2	6.2	contig00009_-_tyrosyl-tRNA_synthetase	1	0.0268	0.3368
6.01	6.01	29.1	contig00016_-_DsrE/DsrF-like_family	7	0.0261	0.0161
2	2	9.8	contig00010_-_pgi,_glucose-6-phosphate_isomerase	1	0.0254	0.1322
12.06	12.06	21.1	contig00007_-_Phosphate-binding_protein_PstS_1	16	0.0233	0
10.34	10.34	21.9	contig00001_-_6-phosphogluconate_dehydrogenase	6	0.0227	0.0058
16.59	16.59	20	contig00014_-_multifunctional_aminopeptidase_A	12	0.0209	0.0003
4	4	6.1	contig00014_-_HDOD_domain	2	0.0205	0.1401
2	2	18.2	contig00002_-_RsuA,_16S_rRNA_uridine-516_pseudouridylate_synthase_and_related_pseudouridylate_synthases	1	0.0111	0.0763
4	4	12	contig00004_-_OmpR,_Response_regulators_consisting_of_a_CheY-like_receiver_domain_and_a_DNA-binding_domain	2	0.0111	0.0881
2	2	4.7	contig00001_-_5'-methylthioadenosine_phosphorylase	1		
2	2	5.4	contig00001_-_ompR,_osmolarity_response_regulator	1		
4	4	11.6	contig00003_-_PF03625_domain_protein	2		
2	2	13.6	contig00004_-_Glucokinase	1		
2	2	5.4	contig00005_-_ddl,_D-alanine--D-alanine_ligase	1		
2	2	4.5	contig00005_-_UDP-N-acetylglucosamine_1-carboxyvinyltransferase	1		
2.01	2.01	4.1	contig00006_-_leuS,_leucyl-tRNA_synthetase	1		
2	2.02	9.6	contig00008_-_Cytochrome_C1_family	1		
8	10	23.9	contig00008_-_GlnK,_Nitrogen_regulatory_protein_PII	5		
3.11	3.11	6.5	contig00008_-_NirB,_NAD(P)H-nitrite_reductase	3		
2	2	14	contig00010_-_YciI-like_protein	1		
2	2	3.8	contig00011_-_amidophosphoribosyltransferase	1		
2	2	2	contig00011_-_beta_alanine--pyruvate_transaminase	1		
2	2.09	4.7	contig00011_-_methylmalonic_acid_semialdehyde_dehydrogenase	2		
2	2	5	contig00012_-_upp,_uracil_phosphoribosyltransferase	1		
4	4	3.8	contig00013_-_hscA,_chaperone_protein_HscA	3		
4	4	18.4	contig00015_-_acetyl-CoA_carboxylase_biotin_carboxyl_carrier_protein_subunit	4		
2	2.02	8.5	contig00015_-_ATP-dependent_RNA_helicase_RhlB	2		
2	2	13.6	contig00017_-_monothiol_glutaredoxin,_Grx4_family	1		
2	2	2.9	contig00024_-_rfaD,_ADP-L-glycero-D-mannoheptose-6-epimerase	1		
2	2	4.8	contig00027_-_TonB-dependent_siderophore_receptor	1		

Unused	Total	% Cov	Accession #	Peptides (95%)	116:115	PVal 116:115
97.08	97.08	60	contig00015_-_60_kDa_chaperonin	107	19.2309	0
68.35	68.35	46.4	contig00002_-_NADH_dehydrogenase	48	1.556	0.6801
47.72	47.72	73.3	contig00004_-_outer_membrane_protein_A	44	1.3183	0.0573
45.99	45.99	62.8	contig00006_-_ectoine/hydroxyectoine_ABC_transporter_solute-binding_protein	44	59.1562	0.0001
43.59	43.59	39.8	contig00022_-_Carboxysome_shell_peptide_mid-region	42	0.0608	0.206
41.87	41.87	51.3	contig00002_-_dnaK,_molecular_chaperone_DnaK	33	7.656	0
39.73	39.73	20.3	contig00011_-_FimV,_Tfp_pilus_assembly_protein_FimV	26	8.4723	0.0002
38.78	38.78	37.1	contig00008_-_Putative_outer_membrane_cytochrome_c	50	0.7798	0.6088
36.97	37.02	30.4	contig00016_-_Protein_AsmA	25	0.5248	0.0266
36.27	36.27	41.2	contig00022_-_fructose-1,6-bisphosphate_aldolase	29	0.2512	0.0001
35.75	35.75	83.2	contig00026_-_Major_outer_membrane_protein_P.IB	66	5.8076	0.0055
34.54	34.58	61.5	contig00008_-_Rusticyanin_protein	52	4.7424	0.0114
32.43	32.43	62.1	contig00006_-_Rubrerythrin_protein	66	7.656	0.0092
31.82	31.82	52.7	contig00044_-_elongation_factor_Tu	33	1.3677	0.5957
31.17	32.54	35	contig00002_-_rpsA,_30S_ribosomal_protein_S1	19	9.4624	0.0031
30.92	30.92	37.6	contig00022_-_Ribulose_bisphosphate_carboxylase_large_chain	26	0.7047	0.1117
30.46	30.46	43.1	contig00002_-_Probable_parvulin-type_peptidyl-prolyl_cis-trans_isomerase	24	7.1121	0
28.36	28.36	16.1	contig00030_-_DNA-directed_RNA_polymerase_subunit_beta	16	1.028	0.7985
27.76	27.76	50.8	contig00003_-_ATP_synthase_subunit_alpha	20	1.1803	0.684
27.28	27.28	23.7	contig00030_-_elongation_factor_G	16	0.9908	0.866
26.72	26.72	43.9	contig00003_-_phosphoribulokinase	20	3.8019	0.4477
26.61	26.61	29.8	contig00003_-_F0F1_ATP_synthase_subunit_beta	22	3.9811	0.1086
26.12	26.12	47.3	contig00001_-_2,3,4,5-tetrahydropyridine-2,6-dicarboxylate_N-succinyltransferase	16	10.2802	0
25.88	25.88	39	contig00003_-_glnA,_glutamine_synthetase	21	0.5754	0.4324
25.61	25.61	33.7	contig00004_-_mdoG,_glucan_biosynthesis_protein_G	16	1.5417	0.4183
24.06	24.06	58.4	contig00006_-_rplB,_50S_ribosomal_protein_L2	15	0.1871	0.0139
23.91	23.91	36.8	contig00008_-_Carbohydrate-selective_porin,_OprB_family	19	0.7943	0.3913
23.78	24.31	40.4	contig00006_-_DNA-directed_RNA_polymerase_subunit_alpha	17	0.278	0.0014
23.71	23.71	57.6	contig00006_-_rplF,_50S_ribosomal_protein_L6	20	4.4875	0.1915
23.4	23.4	30.2	contig00025_-_BtuB,_Outer_membrane_cobalamin_receptor_protein	19	4.529	0.0024
23.3	23.3	60.1	contig00010_-_30S_ribosomal_protein_S6	15	2.7542	0.0011
23.27	23.27	37.9	contig00002_-_Trigger_factor	25	3.1915	0.2755
22.46	22.46	61.5	contig00056_-_Gram-negative_porin	47	6.0813	0.0827
22.23	22.23	62.5	contig00015_-_10_kDa_chaperonin	23	21.677	0.0001
22.03	22.03	27.1	contig00003_-_Prc,_Periplasmic_protease	14	1.1695	0.9917
21.96	21.96	73.3	contig00066_-_Major_carboxysome_shell_protein_1A	38	0.0692	0.0491
21.56	21.59	45.3	contig00004_-_HdrA,_Heterodisulfide_reductase,_subunit_A_and_related_polyferredoxins	14	2.2699	0.4701

21.35	21.35	66.5	contig00001_-_frr,_ribosome_recycling_factor	15	3.4674	0.0002
20.56	20.56	48	contig00002_-_signal_peptidase_I	14	1.803	0.0315
20.36	20.36	35.6	contig00004_-_Chaperone_SurA	11	5.3951	0.0008
19.75	21.77	29.9	contig00013_-_eno,_enolase	15	0.5916	0.2923
19.7	19.7	30.4	contig00022_-_pyruvate_kinase	17	1.0864	0.8141
17.76	17.76	18.1	contig00001_-_polynucleotide_phosphorylase/polyadenylase	9	2.0137	0.3954
17.71	17.71	28.4	contig00003_-_Periplasmic_binding_protein	16	4.6132	0.0014
17.6	17.6	39.5	contig00006_-_LysM_domain/BON_superfamily_protein	34	10.666	0.0003
17.35	17.35	53.5	contig00001_-_tsf,_elongation_factor_Ts	14	4.7424	0.0029
16.81	16.81	37.4	contig00012_-_50S_ribosomal_protein_L25/general_stress_protein_Ctc	15	39.0841	0.0199
16.77	17.73	20.7	contig00017_-_UshA,_5'-nucleotidase/2',3'-cyclic_phosphodiesterase_and_related_esterases	14	0.0731	0
16.62	16.62	29.6	contig00022_-_Glyceraldehyde-3-phosphate_dehydrogenase	11	0.3767	0.0729
16.59	16.59	20	contig00014_-_multifunctional_aminopeptidase_A	12	0.0191	0.0002
16.05	16.05	41.1	contig00008_-_hypothetical_protein	12	4.6132	0.0081
16	16	48	contig00030_-_50S_ribosomal_protein_L7/L12	13	12.5892	0.0027
16	16	42.1	contig00013_-_Transmembrane_protein	9	5.9156	0.0055
16	16	23.3	contig00027_-_Dienelactone_hydrolase_and_related_enzymes	12	2.8576	0.0988
15.72	15.72	57.1	contig00001_-_RNA_polymerase-binding_transcription_factor_DksA	10	10.0925	0.0038
15.33	15.33	57.7	contig00030_-_30S_ribosomal_protein_S7	13	1.2589	0.7783
15.33	15.33	34	contig00003_-_preprotein_translocase_subunit_SecB	9	4.9659	0.0297
15.27	15.27	12.5	contig00001_-_Phosphoesterase_family	9	0.7447	0.2618
15.27	15.27	15.6	contig00021_-_Protein_translocase_subunit_SecD	8	0.2582	0.125
14.98	14.98	67.1	contig00016_-_SirA-like_protein	14	8.8716	0.0215
14.88	14.88	22.1	contig00002_-_periplasmic_folding_chaperone	8	0.0417	0.0008
14.7	14.7	30.8	contig00004_-_tolB,_translocation_protein_TolB	14	1.6749	0.0794
14.68	14.68	33.1	contig00014_-_ketol-acid_reductoisomerase	11	0.207	0.4211
14.63	14.71	38.3	contig00030_-_50S_ribosomal_protein_L10	9	0.1923	0.0183
14.28	14.28	11.2	contig00001_-_aconitate_hydratase	8	0.5495	0.4962
14.13	14.13	56.7	contig00009_-_CspC,_Cold_shock_proteins	11	9.1201	0.1168
14.02	14.02	29.1	contig00003_-_F0F1_ATP_synthase_subunit_alpha	10	1.1066	0.9032
14	14.03	23.5	contig00022_-_Phosphoglycerate_kinase	9	0.3565	0.4797
14	14	15.3	contig00024_-<br>_Porins_form_aqueous_channels_for_diffusion_of_hydrophillic_molecules_across_the_outer_membrane	8	5.2481	0.0004
14	14	48.1	contig00016_-_oxidative_damage_protection_protein	16	3.4356	0.1269
14	14	39.3	contig00001_-_Translation_initiation_factor_IF-3	8	0.2831	0.0043
13.72	13.72	50.5	contig00004_-_Sulphur_oxidation_protein_SoxZ	16	2.9923	0.0338
13.6	15.69	22	contig00004_-_DacC,_D-alanyl-D-alanine_carboxypeptidase	9	0.7798	0.1627
13.6	13.6	23.4	contig00007_-_LysM_domain/BON_superfamily_protein	7	1.2134	0.5662
13.55	13.55	71.6	contig00006_-_50S_ribosomal_protein_L29	9	2.9376	0.0076

13.44	14.82	37.2	contig00001_-_RND_family_efflux_transporter,_MFP_subunit	11	0.0895	0.0153
13.32	13.32	22.3	contig00004_-_Multidrug_resistance_outer_membrane_protein_MdtP	7	1.4454	0.6796
13.19	13.19	34.1	contig00006_-_rplC,_50S_ribosomal_protein_L3	8	0.8872	0.3399
13.15	13.15	36.5	contig00007_-_putative_outer_membrane_lipoprotein	11	3.3729	0.2425
13.08	13.08	21.1	contig00036_-_cysteine_desulfurase	7	2.1478	0.1238
12.83	12.83	48.4	contig00002_-_bacterial_(prokaryotic)_histone_like_domain	18	4.0926	0.3315
12.74	12.74	26.3	contig00004_-_tol-pal_system_protein_YbgF	11	3.2509	0.0184
12.69	12.69	22.8	contig00003_-_fructose-1,6-bisphosphatase	9	0.3133	0.336
12.47	12.47	31.9	contig00002_-_greA,_transcription_elongation_factor_GreA	8	11.1686	0.1922
12.39	12.39	42.7	contig00007_-_Protein_PilG	7	3.1046	0.1085
12.33	12.33	22.1	contig00004_-_HdrB,_Heterodisulfide_reductase,_subunit_B	12	0.3631	0.0879
12.23	12.25	39.9	contig00005_-_Toluene_tolerance,_Ttg2	9	6.7298	0.001
12.12	12.12	15.8	contig00002_-_ATP-dependent_zinc_metalloprotease_FtsH	7	12.0226	0.0004
12.11	12.11	41.1	contig00004_-_Acireductone_dioxygenase	7	0.3221	0.088
12.06	12.06	21.1	contig00007_-_Phosphate-binding_protein_PstS_1	16	0.027	0.0005
12.05	12.05	29.1	contig00008_-_CyoA,_Heme/copper-type_cytochrome/quinol_oxidases,_subunit_2	10	6.8549	0.0722
12.04	12.04	43.3	contig00017_-_Putative_lipoprotein	9	7.9433	0.0015
12	12	29.3	contig00017_-_transcriptional_regulator_PhoU	8	0.5598	0.9606
12	12	37.2	contig00005_-_Putative_peptidoglycan_binding_domain	8	0.0316	0.0001
12	12	27.3	contig00030_-_50S_ribosomal_protein_L11	6	0.4786	0.3645
11.86	11.86	28	contig00004_-_peptidoglycan-associated_lipoprotein	21	16.7494	0.1362
11.75	11.75	14.9	contig00012_-_hypothetical_protein	6	0.1047	0.0672
11.73	11.73	42.6	contig00006_-_rpsE,_30S_ribosomal_protein_S5	8	0.2831	0.0293
11.64	11.64	47.3	contig00006_-_rplE,_50S_ribosomal_protein_L5	7	0.1528	0.1354
11.45	11.45	21.8	contig00010_-_glyA,_serine_hydroxymethyltransferase	7	0.4656	0.113
11.37	11.37	22.7	contig00011_-_HflK_protein	8	6.368	0.2007
11.18	11.43	16	contig00005_-_ResB-like_family	9	1.0186	0.6053
11.05	11.05	26.7	contig00006_-_rpsD,_30S_ribosomal_protein_S4	6	0.4656	0.1677
11.02	11.02	36.9	contig00015_-_Thioredoxin_protein	9	4.0926	0.3212
10.97	10.97	18	contig00005_-_Thiazole_synthase	8	0.5861	0.1561
10.73	10.73	20.1	contig00002_-_GalU,_UDP-glucose_pyrophosphorylase	5	0.1127	0.0053
10.71	10.85	22.1	contig00001_-_Ubiquinol_oxidase_subunit_2	9	1.6596	0.3879
10.7	10.7	28.6	contig00006_-_Uncharacterized_enzyme_involved_in_biosynthesis_of_extracellular_polysaccharides	12	3.3113	0.0639
10.49	10.49	12.3	contig00001_-_MrcA,_Membrane_carboxypeptidase/penicillin-binding_protein	8	0.0319	0.0059
10.34	10.34	21.9	contig00001_-_6-phosphogluconate_dehydrogenase	6	0.0187	0.0044
10.34	10.34	67.4	contig00016_-_DNA-directed_RNA_polymerase_subunit_omega	8	10.4713	0.3341
10.13	10.13	24.9	contig00002_-_Universally_conserved_protein	8	8.1658	0.0021
10.09	10.09	32.6	contig00001_-_rpsB,_30S_ribosomal_protein_S2	9	0.6855	0.2397
10.07	10.07	61.2	contig00006_-_rplW,_50S_ribosomal_protein_L23	6	1.3428	0.6094

10.02	10.02	46.6	contig00006_-_rpsM,_30S_ribosomal_protein_S13	6	0.1542	0.0213
10.02	10.02	21.7	contig00016_-_multidrug_efflux_system_protein_EmrA	7	0.4786	0.1983
10.02	10.02	55.7	contig00001_-_3-ketoacyl-(Acyl-carrier-protein)_reductase	7	3.1623	0.0475
10	10	24.2	contig00030_-_rplA,_50S_ribosomal_protein_L1	6	0.871	0.9398
10	10	20.1	contig00013_-_Cytoskeleton_protein_RodZ	5	7.5162	0.0194
10	10	64.8	contig00006_-_rplX,_50S_ribosomal_protein_L24	8	2.704	0.1484
10	10	53.3	contig00002_-_heat_shock_protein_GrpE	7	3.1623	0.0277
10	10	32.9	contig00013_-_ndk,_mulitfunctional_nucleoside_diphosphate_kinase/apyrimidinic_endonuclease/3	10	2.5119	0.0853
10	10	16.3	contig00003_-_Probable_chromosome-partitioning_protein_ParB	6	0.7656	0.2191
9.91	9.91	20.9	contig00012_-_RND_family_efflux_transporter,_MFP_subunit	8	0.7047	0.1563
9.75	9.75	43.2	contig00006_-_rpsH,_30S_ribosomal_protein_S8	8	0.929	0.4232
9.5	9.58	21.1	contig00011_-_HflC_protein	5	2.1086	0.0053
9.45	9.45	52.8	contig00022_-_Ribulose_bisphosphate_carboxylase_small_chain	12	0.0597	0.02
9.28	9.28	41.5	contig00006_-_30S_ribosomal_protein_S11	8	0.1722	0.5863
9.13	9.13	54.1	contig00014_-_LysR,_Transcriptional_regulator	6	0.1148	0.0213
8.86	8.92	24.1	contig00019_-_Outer_membrane_beta-barrel_domain_protein	7	2.704	0.7356
8.85	8.85	28.5	contig00010_-_50S_ribosomal_protein_L9	6	4.3652	0.07
8.71	8.71	14.6	contig00025_-_5-methyltetrahydropteroyltriglutamate--_homocysteine_S-methyltransferase	6	3.0479	0.1979
8.54	8.54	33.9	contig00006_-_single-stranded_DNA-binding_protein	5	1.0765	0.3539
8.53	8.53	25.4	contig00016_-_SoxAX_cytochrome_complex_subunit_A	5	0.6792	0.2807
8.52	8.52	15.1	contig00016_-_tolC,_outer_membrane_channel_protein	10	3.4674	0.0237
8.49	8.49	24	contig00014_-_Chemotaxis_phosphatase,_CheZ	6	5.8076	0.0427
8.45	10.79	15.4	contig00006_-_Outer_membrane_protein_assembly_factor_BamC	15	5.445	0.1488
8.4	8.4	15.5	contig00008_-_signal_peptide_peptidase_SppA,_67K_type	4	0.0586	0.0101
8.34	8.34	17.4	contig00004_-_recA,_recombinase_A	6	0.0847	0.0056
8.26	8.26	8.8	contig00006_-_Zn-finger_containing_NTP_pyrophosphohydrolase	4	1.3183	0.4615
8.25	8.25	37.3	contig00001_-_hypothetical_protein	5	3.1915	0.8069
8.19	8.19	33.9	contig00010_-_Membrane_protein_involved_in_aromatic_hydrocarbon_degradation	22	4.4875	0.0934
8.15	8.15	55.8	contig00006_-_rpsJ,_30S_ribosomal_protein_S10	5	1.8197	0.4729
8.14	8.14	32.6	contig00009_-_tpx,_lipid_hydroperoxide_peroxidase	7	0.3404	0.5161
8.12	8.12	27.7	contig00001_-_Predicted_peroxiredoxins	7	0.1738	0.2306
8.11	8.11	27.6	contig00004_-_transaldolase	6	0.6252	0.0815
8.08	8.08	18.9	contig00026_-_Protein_CbbQ	5	2.7542	0.3918
8.06	8.06	51.2	contig00001_-_NADPH-dependent_7-cyano-7-deazaguanine_reductase	4	4.8306	0.5093
8.05	8.05	42.2	contig00007_-_Protein_PilH	5	15.7036	0.0154
8.04	8.04	18.4	contig00006_-_RND_family_efflux_transporter,_MFP_subunit	5	0.7047	0.2539
8.03	8.03	25.9	contig00008_-_SCO1/SenC_protein	4	0.0608	0.0006
8.01	8.03	7.5	contig00005_-_Multidrug_resistance_protein_MdtB	4	0.3133	0.3375
8.01	8.01	19.9	contig00001_-_FabD,_(acyl-carrier-protein)_S-malonyltransferase	6	1.7701	0.9816

8	10	15.1	contig00012_-_HDOD_domain	5	0.9376	0.5285
8	10	23.9	contig00008_-_GlnK,_Nitrogen_regulatory_protein_PII	5		
8	8	18	contig00009_-_gcvT,_glycine_cleavage_system_aminomethyltransferase_T	4	0.2128	0.0735
8	8	24.8	contig00009_-_Conserved_hypothethical_protein,_UCP019302	4	3.0761	0.0267
8	8	26	contig00017_-_thioredoxin_protein	6	6.8549	0.0662
8	8	32.3	contig00016_-_reactive_intermediate/imine_deaminase	6	14.1906	0.0302
8	8	23.6	contig00006_-_30S_ribosomal_protein_S14	4	0.3467	0.4277
8	8	18.5	contig00005_-_outer_membrane_assembly_lipoprotein_YfiO	4	0.092	0.0579
8	8	23.1	contig00003_-_ATP_synthase_subunit_b	5	16.5959	0.0136
7.92	7.92	38.8	contig00005_-_Ttg2C,_ABC-type_transport_system_involved_in_resistance_to_organic_solvents	4	12.2462	0.0284
7.89	7.89	37.2	contig00022_-_carboxysome_peptide_B	4	1.1695	0.7296
7.79	7.79	13.9	contig00002_-_nusA,_transcription_elongation_factor_NusA	4	1.3932	0.4744
7.62	7.62	12.2	contig00005_-_CirA,_Outer_membrane_receptor_proteins,_mostly_Fe_transport	5	0.8472	0.8068
7.6	7.6	15	contig00017_-_dihydrolipoamide_acetyltransferase	5	1.8535	0.1166
7.48	7.48	25.5	contig00018_-_Gram-negative_porin	7	0.912	0.6482
7.38	7.38	13.7	contig00002_-_acetyl-CoA_synthetase	4	0.7943	0.3036
7.13	7.13	13.8	contig00005_-_Fimbrial_protein_P9-2	11	3.0479	0.1552
7.07	7.07	24.1	contig00004_-_ferredoxin-NADP_reductase	4	1.1066	0.7361
7.03	9.31	17.8	contig00003_-_DNA_polymerase_III_subunit_beta	6	0.2754	0.2024
6.95	6.95	39.1	contig00006_-_rpsC,_30S_ribosomal_protein_S3	5	0.1259	0.1431
6.88	6.88	20.9	contig00026_-_ribulose-phosphate_3-epimerase	4	1	0.6571
6.86	6.86	19.4	contig00007_-_Fimbrial_assembly_protein_PilQ	4	5.5976	0.0868
6.84	6.84	16.9	contig00003_-_pntA,_NAD(P)_transhydrogenase_subunit_alpha	4	0.7586	0.1076
6.77	6.77	14.5	contig00003_-_HemX,_Uncharacterized_enzyme_of_heme_biosynthesis	7	2.5351	0.2098
6.68	6.68	31.9	contig00021_-_yajC,_preprotein_translocase_subunit_YajC	5	40.5508	0.0996
6.57	6.57	34.2	contig00006_-_Cytochrome_c	5	13.9316	0.0577
6.45	6.45	36.3	contig00030_-_rpsL,_30S_ribosomal_protein_S12	5	1.0965	0.4029
6.24	6.24	18.3	contig00003_-_LivK,_ABC-type_branched-chain_amino_acid_transport_systems,_periplasmic_component	4	0.2377	0.2528
6.22	6.22	27.8	contig00005_-_phosphoheptose_isomerase	7	0.3837	0.9462
6.18	6.18	13.6	contig00015_-_rho,_transcription_termination_factor_Rho	3	11.9124	0.1558
6.13	6.13	32.1	contig00005_-_Thiol:disulfide_interchange_protein_DsbA	3	3.1333	0.1945
6.13	6.13	20.6	contig00006_-_Fe-S_oxidoreductase-like_protein	3	0.3311	0.3267
6.08	6.18	17	contig00008_-_AfuA,_ABC-type_Fe3+_transport_system,_periplasmic_component	6	1.1482	0.5225
6.06	6.06	23	contig00012_-_YecA_family_protein	5	0.4446	0.1344
6.03	6.03	14.2	contig00008_-_Outer_membrane_lipoprotein_Slp_family	4	2.0893	0.7853
6.03	6.03	13.8	contig00002_-_Aminoglycoside_phosphotransferase	3	0.2911	0.1557
6.02	6.02	14.1	contig00001_-_rne,_ribonuclease_E	3	3.4674	0.6698
6.02	6.02	27.5	contig00002_-_elongation_factor_P	3	1.2823	0.4053
6.01	6.02	32.7	contig00006_-_ATPase_with_chaperone_activity,_ATP-binding_subunit	3	2.2909	0.419

6.01	6.01	17.3	contig00012_-_gatB,_aspartyl/glutamyl-tRNA_amidotransferase_subunit_B	3	2.208	0.1159
6.01	6.01	22.9	contig00010_-_The_CBS_domain,_is_a_small_domain_originally_identified_in_cystathionine_beta-synthase	4	0.4018	0.2862
6.01	6.01	13.3	contig00008_-_Cytochrome_c4	4	0.955	0.9818
6.01	6.01	43.6	contig00020_-_Bcp,_Peroxiredoxin	3	1.4859	0.4095
6.01	6.01	14	contig00015_-_Disulphide_bond_corrector_protein_DsbC	5	2.5586	0.3761
6.01	6.01	23.9	contig00011_-_FtsH_protease_regulator_HflC	5	1.3062	0.2509
6.01	6.01	29.1	contig00016_-_DsrE/DsrF-like_family	7	0.0174	0.0084
6	6.59	33.7	contig00002_-_SirA-like_protein	3	0.7379	0.1201
6	6.09	12.7	contig00017_-_aceE,_pyruvate_dehydrogenase_subunit_E1	3	0.8241	0.2895
6	6.01	23.6	contig00003_-_OmpR,_Response_regulators_consisting_of_a_CheY-like_receiver_domain	3	0.7379	0.3175
6	6.01	11.9	contig00013_-_outer_membrane_assembly_lipoprotein_YfgL	7	3.767	0.1088
6	6.01	12.1	contig00022_-_transketolase	5	1.2942	0.6845
6	6.01	42.2	contig00006_-_rpsS,_30S_ribosomal_protein_S19	6	1.0666	0.3198
6	6.01	9.3	contig00003_-_Tas,_Predicted_oxidoreductases	3	0.177	0.0601
6	6	25.8	contig00010_-_secreted_protein	3	2.9923	0.0839
6	6	6	contig00009_-_Protein_HelA	3	0.2559	0.0971
6	6	41.2	contig00006_-_rplV,_50S_ribosomal_protein_L22	5	0.52	0.2853
6	6	26.9	contig00001_-_Elongation_factor_Ts	4	5.2	0.1083
6	6	21	contig00026_-_Predicted_redox_protein,_regulator_of_disulfide_bond_formation	4	2.5119	0.3754
6	6	10.2	contig00017_-_hypothetical_protein	3	3.9446	0.2337
6	6	17.8	contig00012_-_Cytochrome_C	3	4.5709	0.3063
6	6	12.2	contig00004_-_HdrC,_Heterodisulfide_reductase,_subunit_C	4	0.8954	0.446
6	6	21.4	contig00004_-_Glucosamine-6-phosphate_isomerases/6-phosphogluconolactonase	4	0.0488	0.0111
6	6	14.5	contig00002_-_tpiA,_triosephosphate_isomerase	3	0.871	0.1071
6	6	21.2	contig00030_-_nusG,_transcription_antitermination_protein_NusG	5	4.6989	0.1473
6	6	11	contig00013_-_type_IV_pilus_biogenesis/stability_protein_PilW	3	10.8643	0.0323
6	6	10.5	contig00007_-_1-(5-phosphoribosyl)-5-[(5-_phosphoribosylamino)methylideneamino]_imidazole-4-carboxamide_isomerase	3	0.787	0.1013
6	6	10	contig00006_-_peptidase_PmbA	3	1.888	0.3834
6	6	25.6	contig00005_-_YfaZ_family_protein	5	1.1803	0.59
5.96	6	11.8	contig00013_-_Putative_methyl-accepting_chemotaxis_AlkN	3	0.9462	0.9848
5.89	6.02	25.6	contig00006_-_DsrE/DsrF-like_family	4	0.169	0.1151
5.82	5.82	19.8	contig00005_-_RbsK,_Sugar_kinases,_ribokinase_family	3	1.3305	0.6098
5.78	5.78	4.5	contig00003_-_TonB-dependent_siderophore_receptor	3	0.0384	0.0436
5.77	5.8	43.9	contig00005_-_CopC_domain	6	1.9409	0.1093
5.73	5.73	22.3	contig00004_-_inorganic_pyrophosphatase	3	8.4723	0.2167
5.62	5.62	11.6	contig00005_-_argJ,_bifunctional_ornithine_acetyltransferase/N-acetylglutamate_synthase_protein	3	3.6308	0.1497
5.44	5.44	26.7	contig00006_-_arsenate_reductase	5	6.0256	0.2397
5.35	5.35	8.6	contig00003_-_phosphoenolpyruvate_synthase	3	1.0965	0.2362

5.35	5.35	9.1	contig00003_-_putative_quinone_oxidoreductase,_YhdH/YhfP_family	3	0.912	0.7895
5.31	5.31	18.7	contig00016_-_Curved_DNA-binding_protein	3	2.8054	0.1082
5.31	5.31	41.3	contig00009_-_molybdopterin_biosynthesis_protein_MoeB	5	0.597	0.4624
5.1	5.1	39.9	contig00015_-_Dihydroneopterin_aldolase	3	1.3552	0.0391
4.92	4.92	7.7	contig00010_-_pyruvate_dehydrogenase	3	1.4588	0.3217
4.91	4.91	32.2	contig00016_-_IbpA,_Molecular_chaperone	4	2.1878	0.3271
4.9	4.93	29.9	contig00004_-_Putative_lipoprotein	3	1.0666	0.58
4.82	4.82	14	contig00009_-_N-ethylmaleimide_reductase	3	1.2023	0.7948
4.8	4.8	42.7	contig00026_-_Putative_pterin-4_alpha-carbinolamine_dehydratase-like_protein	5	0.3311	0.4088
4.78	4.78	52.8	contig00001_-_infA,_translation_initiation_factor_IF-1	3	1.8707	0.2712
4.76	4.76	19.7	contig00009_-_gabD1,_succinic_semialdehyde_dehydrogenase	3	0.1096	0.1058
4.75	4.75	16.1	contig00014_-_DdpA,_ABC-type_dipeptide_transport_system,_periplasmic_component	3	4.6132	0.0261
4.74	4.74	11.6	contig00002_-_Translation_initiation_factor_IF-2	3	1.0965	0.4912
4.63	4.63	12.6	contig00003_-_PKD_domain_containing_protein	3	1.6144	0.213
4.6	4.6	51.7	contig00010_-_hypothetical_protein	11	4.0551	0.3195
4.57	4.57	24.9	contig00007_-_Pilus_assembly_protein,_PilP	3	2.5351	0.3718
4.53	4.63	12.6	contig00006_-_Pyruvate/2-oxoglutarate_dehydrogenase_complex,_dihydrolipoamide_dehydrogenase_(E3)	2	0.5445	0.2165
4.47	4.47	15.9	contig00011_-_L,D-transpeptidase_catalytic_domain	4	0.7727	0.534
4.43	4.43	25.2	contig00002_-_Protein_GrpE	4	5.7016	0.2429
4.37	5.07	25.2	contig00011_-_leuD,_isopropylmalate_isomerase_small_subunit	3	0.3631	0.0064
4.37	4.37	24.5	contig00010_-_nusB,_transcription_antitermination_protein_NusB	2	1.406	0.2182
4.36	4.37	23.1	contig00008_-_Uncharacterized_isochorismatase_family_protein_YwoC	3	0.1754	0.0852
4.32	4.33	16.3	contig00005_-_ATP-dependent_chaperone_ClpB	2	2.9376	0.3067
4.3	4.3	22.5	contig00001_-_lolA,_lipoprotein_chaperone	3	12.7057	0.3561
4.28	4.28	50.6	contig00003_-_rpmA,_50S_ribosomal_protein_L27	3	1.1376	0.2259
4.22	4.22	11.5	contig00002_-_pyridoxine_5'-phosphate_synthase	2	0.5702	0.7365
4.18	4.18	11.5	contig00016_-_ATP-dependent_protease_subunit_HslV	3	2.9376	0.317
4.17	4.17	21.9	contig00002_-_oligoribonuclease	3	3.8371	0.1138
4.17	4.17	17.9	contig00002_-_Tetratricopeptide_TPR_2_repeat_protein	2	6.7298	0.1413
4.15	4.15	23	contig00026_-_Bacterioferritin_protein	3	13.5519	0.2174
4.14	4.15	22.8	contig00002_-_Thioredoxin_domain-containing_protein	2	10.3753	0.042
4.12	4.14	24.8	contig00006_-_This_family_is_most_closely_related_to_the_GT1_family_of_glycosyltransferases	3	1.556	0.4783
4.12	4.12	28.6	contig00001_-_putative_nucleotide-binding_protein	3	3.3113	0.1097
4.12	4.12	11.6	contig00001_-_TolC,_Outer_membrane_protein	2	0.2704	0.1926
4.11	4.11	23.7	contig00007_-_Yqey-like_protein	5	1.7378	0.4164
4.08	4.08	19.8	contig00002_-_Cupin_domain	2	0.2489	0.205
4.06	4.06	14.3	contig00021_-_inositol_monophosphatase	3	2.421	0.3723
4.06	4.06	13.7	contig00003_-_Sporulation_related_domain	3	3.6644	0.3998
4.05	4.05	18	contig00003_-_F0F1_ATP_synthase_subunit_delta	2	1.1066	0.8666

4.05	4.05	18.2	contig00008_-_ubiquinol-cytochrome_c_reductase,_iron-sulfur_subunit	2	1.977	0.3278
4.05	4.05	13	contig00002_-_Cytidylate_kinase-like_family	2	1.7061	0.3514
4.03	4.03	17.2	contig00016_-_rph,_ribonuclease_PH	2	2.884	0.2975
4.03	4.03	13.8	contig00005_-_Cation_efflux_system_protein_CzcB	2	0.0991	0.0398
4.02	4.02	9.2	contig00002_-_DNA_gyrase_subunit_A	2	0.2679	0.1796
4.02	4.02	29.4	contig00014_-_short_chain_dehydrogenase	3	1	0.9294
4.02	4.02	14.7	contig00035_-_TolQ,_Biopolymer_transport_proteins	2	0.3945	0.2316
4.01	4.03	12.2	contig00001_-_fabG,_3-ketoacyl-(acyl-carrier-protein)_reductase	2	0.863	0.8882
4.01	4.01	20.9	contig00009_-_Membrane_Fusion_Protein_cluster_2	3	0.0313	0.0749
4.01	4.01	7.4	contig00001_-_periplasmic_serine_protease,_Do/DeqQ_family	2	0.9462	0.9702
4.01	4.01	8.4	contig00007_-_Phosphomannomutase/phosphoglucomutase	2	3.02	0.9568
4.01	4.01	14.4	contig00006_-_rbcR,_LysR_transcriptional_regulator	2	2.2909	0.4076
4.01	4.01	23.9	contig00006_-_50S_ribosomal_protein_L18	3	1.4588	0.6686
4.01	4.01	20.3	contig00011_-_Putative_signal_peptide_protein	4	0.3133	0.2528
4.01	4.01	14.5	contig00010_-_Nucleoprotein/polynucleotide-associated_enzyme	2	1.0093	0.8375
4.01	4.01	24.4	contig00004_-_Penicillin-binding_protein_5	2	1.1695	0.7627
4	6	23	contig00016_-_Putative_phosphoribosylformimino-5-aminoimidazole_carboxamide_ribotide_isomerase	3	2.355	0.3614
4	4.29	36.9	contig00026_-_rpsI,_30S_ribosomal_protein_S9	3	1.0765	0.8308
4	4.04	8.2	contig00015_-_BisC,_Anaerobic_dehydrogenases,_typically_selenocysteine-containing	2	0.8872	0.8885
4	4.02	12.4	contig00009_-_Prc,_Periplasmic_protease	2	0.0752	0.1122
4	4.01	27.5	contig00024_-_3-hydroxydecanoyl-(acyl_carrier_protein)_dehydratase	2	6.792	0.3099
4	4	27.7	contig00007_-_Pilus_assembly_protein,_PilO	2	0.4365	0.3622
4	4	27.6	contig00026_-_2-nonaprenyl-3-methyl-6-methoxy-1,4-benzoquinol_hydroxylase	2	2.0137	0.5839
4	4	15.7	contig00025_-_DsrE/DsrF/DrsH-like_family	2	0.169	0.1757
4	4	8.3	contig00020_-_Outer_membrane_beta_barrel_protein	2	1.5136	0.3459
4	4	6.4	contig00003_-_ileS,_isoleucyl-tRNA_synthetase	2	0.5346	0.0961
4	4	37.2	contig00030_-_Elongation_factor_G_C-terminus	3	1.2134	0.4902
4	4	24.7	contig00026_-_rplM,_50S_ribosomal_protein_L13	3	0.8318	0.6692
4	4	21.7	contig00015_-_hypothetical_protein	2	1.7061	0.5749
4	4	26	contig00011_-_Predicted_peroxiredoxins	10	0.8395	0.6231
4	4	11.9	contig00009_-_glycine_dehydrogenase_subunit_2	2	5.4954	0.0449
4	4	8.6	contig00009_-_glycine_dehydrogenase_subunit_1	4	0.912	0.4735
4	4	11.3	contig00005_-_succinyl-CoA_synthetase_subunit_alpha	3	0.3342	0.2305
4	4	6.1	contig00002_-_mltD,_membrane-bound_lytic_murein_transglycosylase_D	3	0.2051	0.1554
4	4	29	contig00002_-_Universal_stress_protein_MJ0531	2	9.4624	0.4102
4	4	17.3	contig00002_-_DsrE/DsrF-like_family	3	0.3981	0.2891
4	4	6.9	contig00020_-_Succinate-semialdehyde_dehydrogenase_[NADP(+)]_GabD	3	2.2491	0.3513
4	4	23.7	contig00014_-_Uncharacterized_BCR,_YaiI/YqxD_family_COG1671	2	5.0582	0.1499
4	4	30.2	contig00013_-_RNA_recognition_motif	2	1.556	0.4534

4	4	8	contig00012_-_GTP-binding_protein_YchF	4	0.929	0.7981
4	4	39.6	contig00007_-_Sec-independent_protein_translocase_protein_TatA	2	1.5996	0.5113
4	4	9.9	contig00007_-_Orotate_phosphoribosyltransferase	3	1.3932	0.4928
4	4	33.7	contig00006_-_rpsP,_30S_ribosomal_protein_S16	2	0.3837	0.3046
4	4	24.8	contig00006_-_iojap-like_ribosome-associated_protein	2	1.888	0.249
4	4	4.5	contig00006_-_glpC,_sn-glycerol-3-phosphate_dehydrogenase_subunit_C	2	1.5417	0.4592
4	4	30.1	contig00006_-_Putative_lipoprotein	2	1.3932	0.4513
4	4	15.1	contig00006_-_Proline-rich_region	2	0.0344	0.1448
4	4	43.2	contig00006_-_50S_ribosomal_protein_L15	2	0.2582	0.1803
4	4	19	contig00005_-_UspA_domain_protein	3	2.3988	0.3561
4	4	11.1	contig00005_-_Putative_NADH_dehydrogenase/NAD(P)H_nitroreductase_AF_0226	2	0.8017	0.6135
4	4	12	contig00004_-_OmpR,_Response_regulators_consisting_of_a_CheY-like_receiver_domain	2	0.0118	0.1626
4	4	7.7	contig00003_-_glyQ,_glycyl-tRNA_synthetase_subunit_alpha	2	0.1406	0.2081
4	4	11.6	contig00003_-_PF03625_domain_protein	2		
4	4	24.2	contig00002_-_ihfB,_integration_host_factor_subunit_beta	2	0.9727	0.9356
4	4	2.8	contig00026_-_anthranilate_synthase_component_I	2	4.7863	0.3168
4	4	25.8	contig00022_-_carboxysome_peptide_A	5	0.8551	0.695
4	4	19.1	contig00017_-_minE,_cell_division_topological_specificity_factor_MinE	2	1.0864	0.9049
4	4	17.9	contig00016_-_rpmE,_50S_ribosomal_protein_L31	3	3.2211	0.2637
4	4	6.2	contig00016_-_periplasmic_serine_protease,_Do/DeqQ_family	2	0.263	0.5096
4	4	18.4	contig00015_-_acetyl-CoA_carboxylase_biotin_carboxyl_carrier_protein_subunit	4		
4	4	6.1	contig00014_-_HDOD_domain	2	0.015	0.1026
4	4	3.8	contig00013_-_hscA,_chaperone_protein_HscA	3		
4	4	16.6	contig00012_-_EVE_domain	2	0.5598	0.2789
4	4	1.9	contig00008_-_cytochrome_c_oxidase,_subunit_I	2	0.0425	0.1176
4	4	9.9	contig00008_-_Hemerythrin_HHE_cation_binding_domain	2	0.278	0.3221
4	4	5.4	contig00005_-_glutamate-1-semialdehyde_aminotransferase	2	0.4055	0.5546
4	4	16.9	contig00005_-_PTS_IIA-like_nitrogen-regulatory_protein_PtsN	2	1.0965	0.9698
4	4	19	contig00005_-_ManX,_Phosphotransferase_system,_mannose/fructose-specific_component_IIA	2	0.4875	0.1293
4	4	5.4	contig00003_-_hemC,_porphobilinogen_deaminase	2	1.1169	0.8435
4	4	18.3	contig00001_-_PII-like_signaling_protein	2	2.2491	0.3156
3.92	4	7.2	contig00003_-_dihydroorotate_dehydrogenase_1B	2	0.4406	0.1477
3.92	4	34.4	contig00001_-_Trm112p-like_protein	2	2.355	0.4616
3.89	3.89	11.7	contig00006_-_putative_GTP_cyclohydrolase	2	1.0568	0.534
3.85	3.85	21.2	contig00005_-_Cell_division_protein_ZapD	2	18.3654	0.0846
3.82	3.82	5.6	contig00003_-_ATP_synthase_epsilon_chain	2	1.2246	0.6129
3.81	3.81	15.8	contig00006_-_tldD,_protease_TldD	3	2.208	0.3696
3.8	3.8	14.8	contig00002_-_Pterin_4_alpha_carbinolamine_dehydratase	2	0.6194	0.355
3.78	3.78	40.7	contig00010_-_Putative_cytoplasmic_protein	2	0.2208	0.1624

3.75	3.75	14.3	contig00003_-_Glutaredoxin,_GrxC_family	2	0.2188	0.4264
3.66	3.66	9	contig00004_-_SuhB,_Archaeal_fructose-1,6-bisphosphatase_and_related_enzymes	2	8.3946	0.3481
3.64	3.76	14.7	contig00017_-_dihydrolipoamide_dehydrogenase	2	2.208	0.3477
3.62	3.63	14.8	contig00007_-_RNA_polymerase_sigma_factor_RpoD	2	0.9727	0.6228
3.62	3.62	16.2	contig00026_-_Cytochrome_c553	2	0.2032	0.2267
3.59	3.59	25.9	contig00004_-_Phospholipid-binding_protein	2	0.787	0.6323
3.54	3.58	3.9	contig00007_-_phosphoglyceromutase	2	0.6368	0.4059
3.48	3.48	5	contig00022_-_CheA,_Chemotaxis_protein_histidine_kinase_and_related_kinases	2	0.2679	0.1848
3.47	3.56	12.7	contig00016_-_VacJ,_Surface_lipoprotein	2	1.1272	0.6783
3.41	3.41	14.8	contig00013_-_acetyl-CoA_carboxylase_carboxyltransferase_subunit_alpha	2	17.8649	0.2168
3.39	3.39	46.9	contig00008_-_Membrane_fusogenic_activity	5	0.6486	0.4895
3.29	5.56	14.9	contig00001_-_clpA,_ATP-dependent_Clp_protease_ATP-binding_subunit	3	1.028	0.8824
3.28	3.28	14.4	contig00016_-_dapF,_diaminopimelate_epimerase	2	0.8241	0.2607
3.26	3.26	8.4	contig00001_-_Phosphoketolase	3	0.0406	0.1345
3.2	3.2	47.3	contig00015_-_50S_ribosomal_protein_L33	3	2.3988	0.3698
3.14	3.14	8.9	contig00002_-_Lon,_ATP-dependent_Lon_protease,_bacterial_type	3	1.7219	0.1702
3.11	3.11	6.5	contig00008_-_NirB,_NAD(P)H-nitrite_reductase	3		
3.1	3.1	16.3	contig00001_-_PepB,_Leucyl_aminopeptidase	2	0.0337	0.0792
3.05	3.09	9.4	contig00003_-_glyS,_glycyl-tRNA_synthetase_subunit_beta	2	0.4169	0.3271
3.05	3.05	37.9	contig00004_-_glycine_cleavage_system_protein_H	5	3.6308	0.3787
3.03	3.03	21.1	contig00006_-_rplS,_50S_ribosomal_protein_L19	3	1.4859	0.9654
3	3	9.3	contig00002_-_Segregation_and_condensation_protein_B_homolog	2	0.879	0.4925
2.98	2.98	14.8	contig00007_-_DNA_topoisomerase_1	2	2.355	0.248
2.88	2.88	21.5	contig00002_-_peptidyl-prolyl_cis-trans_isomerase_B	4	11.272	0.1574
2.83	2.83	13.6	contig00007_-_pyrroline-5-carboxylate_reductase	2	1.1803	0.5257
2.79	2.79	50.8	contig00006_-_rpmD,_50S_ribosomal_protein_L30	3	1.3428	0.6799
2.76	2.81	12.5	contig00008_-_Tas,_Predicted_oxidoreductases	2	0.1343	0.1233
2.74	2.74	8.8	contig00007_-_DNA_topoisomerase_I	2	2.5119	0.3464
2.62	2.62	16.5	contig00012_-_ribose-5-phosphate_isomerase_A	2	0.3597	0.2788
2.55	2.55	15.2	contig00010_-_bifunctional_3,4-dihydroxy-2-butanone_4-phosphate_synthase/GTP_cyclohydrolase_II-like_protein	2	0.8318	0.4
2.51	2.51	19.4	contig00004_-_Sulfur_oxidation_protein_SoxY	2	4.2462	0.3131
2.48	2.48	16.5	contig00004_-_Protein_TolB	2	1.7061	0.5795
2.47	2.47	9.6	contig00007_-_Hpt_domain	2	0.9638	0.3364
2.44	2.45	4.8	contig00005_-_Cellulose_synthase_operon_protein_C_C-terminus	2	0.631	0.4278
2.43	2.43	34.3	contig00001_-_Lipoprotein	2	2.8054	0.4353
2.38	2.38	19.2	contig00001_-_nudF,_ADP-ribose_pyrophosphatase_NudF	2	3.3113	0.0411
2.38	2.38	13.6	contig00015_-_Domain_of_unknown_function_(DUF4340)	1	0.2377	0.2223
2.36	2.37	5.6	contig00002_-_clpX,_ATP-dependent_protease_ATP-binding_subunit_ClpX	1	1.3183	0.5975
2.36	2.36	6.3	contig00022_-_S-adenosylmethionine_synthetase	1	0.4742	0.315

2.33	2.33	8.5	contig00025_-_Cobalamin_adenosyltransferase	2	1.1912	0.7261
2.32	2.33	21.9	contig00008_-_Uncharacterized_ACR,_YkgG_family_COG1556	1	0.1629	0.136
2.32	2.32	45.1	contig00006_-_rplN,_50S_ribosomal_protein_L14	2	2.3121	0.6177
2.32	2.32	5.9	contig00012_-_TPR_repeat-containing_protein_PA4667	1	0.0256	0.1653
2.31	2.32	17.9	contig00001_-_hypoxanthine-guanine_phosphoribosyltransferase	1	3.0761	0.3862
2.3	2.3	20.5	contig00014_-_Flagellar_basal_body-associated_protein_FliL	1	3.9084	0.2679
2.29	2.29	16.2	contig00008_-_SCO1_protein_homolog	2	0.3532	0.2306
2.28	2.28	19.8	contig00003_-_30S_ribosomal_protein_S20	2	3.1623	0.2077
2.26	4.2	9.3	contig00012_-_efflux_transporter,_outer_membrane_factor_(OMF)_lipoprotein,_NodT_family	4	0.9462	0.9231
2.26	2.26	14.4	contig00022_-_ATP-dependent_dethiobiotin_synthetase_BioD	2	0.597	0.4335
2.25	2.26	17.7	contig00021_-_hypothetical_protein	1	3.1333	0.5473
2.24	2.24	19.2	contig00004_-_Cytochrome_C_oxidase,_cbb3-type,_subunit_III	2	1.3804	0.5485
2.23	2.23	27	contig00002_-_Cupredoxin-like_domain	1	3.5975	0.1896
2.2	2.2	8.2	contig00004_-_aspartate_kinase	2	0.5105	0.2938
2.16	2.16	26.8	contig00014_-_Tetratricopeptide_TPR_1_repeat-containing_protein	1	1.1482	0.0512
2.14	2.15	5.5	contig00006_-_AcrB,_Cation/multidrug_efflux_pump	1	0.5808	0.3634
2.14	2.14	17.7	contig00026_-_Indole-3-glycerol_phosphate_synthase	1	2.5823	0.581
2.12	2.12	11.2	contig00004_-_antiporter_inner_membrane_protein	1	1.1272	0.7047
2.11	2.11	9.2	contig00020_-_Ferredoxin--NADP_reductase	1	1.1695	0.6298
2.1	2.1	11.6	contig00012_-_prfA,_peptide_chain_release_factor_1	1	1.5136	0.3946
2.08	2.08	17.1	contig00016_-_AhpC/TSA_family	2	5.2	0.0697
2.08	2.08	7.4	contig00005_-_UDP-N-acetylmuramoyl-tripeptide--D-alanyl-D-_alanine_ligase	2	0.2858	0.1666
2.07	2.08	7.5	contig00012_-_Asparaginase	2	5.0582	0.1499
2.06	2.06	7	contig00015_-_dipZ,_thiol:disulfide_interchange_protein_precursor	1	3.1333	0.2102
2.06	2.06	5.5	contig00001_-_NMT1-like_family	1	0.7798	0.6536
2.05	2.05	12.5	contig00010_-_recombination_and_DNA_strand_exchange_inhibitor_protein	1	0.5598	0.204
2.05	2.05	13.2	contig00006_-_Uncharacterized_conserved_secreted_or_membrane_protein	2	2.0137	0.3064
2.05	2.05	4.7	contig00005_-_aspS,_aspartyl-tRNA_synthetase	1	0.7047	0.1926
2.04	2.04	11.3	contig00017_-_ABC_transporter,_phosphonate,_periplasmic_substrate-binding_protein	2	1.2359	0.5719
2.04	2.04	3.7	contig00001_-_phosphoribosylformylglycinamidine_synthase	1	0.5598	0.7532
2.04	2.04	7.2	contig00001_-_fructose-bisphosphate_aldolase	2	0.3311	0.2676
2.04	2.04	27.4	contig00001_-_50S_ribosomal_protein_L32	1	0.7586	0.6144
2.03	2.03	13.6	contig00001_-_General_stress_protein_69	1	0.8954	0.6015
2.03	2.03	4.8	contig00006_-_purT,_phosphoribosylglycinamide_formyltransferase_2	1	0.4742	0.3416
2.03	2.03	12.2	contig00017_-_PleD,_Response_regulator_containing_a_CheY-like_receiver_domain_and_a_GGDEF_domain	2	1.0666	0.823
2.02	2.03	39.7	contig00006_-_50S_ribosomal_protein_L17	1	0.1259	0.1195
2.02	2.02	13.9	contig00006_-_signal_recognition_particle_protein	1	1.5417	0.4523
2.02	2.02	8.4	contig00018_-_Protein_RcaC	1	0.5916	0.4549
2.02	2.02	5	contig00016_-_dihydrolipoamide_dehydrogenase	1	1.3552	0.5672

2.02	2.02	4.9	contig00010_-_glutamate_decarboxylase	1	0.4446	0.2898
2.02	2.02	2.8	contig00003_-_c-di-GMP_phosphodiesterase_class_II	1	2.704	0.2375
2.02	2.02	14.9	contig00002_-_OlmA,_Outer_membrane_lipoprotein_OmlA	2	4.7424	0.1456
2.02	2.02	14.3	contig00001_-_ATP-dependent_Clp_protease_adapter_protein_ClpS	1	2.704	0.402
2.01	4.03	10.5	contig00010_-_Hemolysin_secretion_protein	3	1.0186	0.9567
2.01	2.03	11.3	contig00006_-_heat_shock_protein_90	1	0.157	0.1659
2.01	2.02	4.7	contig00012_-_multidrug_efflux_protein	1	0.3945	0.2461
2.01	2.01	9.9	contig00010_-_dihydroxy-acid_dehydratase	1	1.888	0.3466
2.01	2.01	4.1	contig00006_-_leuS,_leucyl-tRNA_synthetase	1		
2.01	2.01	12	contig00026_-_Bacterial_microcompartments_are_primitive_organelles_composed_entirely_of_protein_subunits	2	1.1169	0.6876
2.01	2.01	11.4	contig00006_-_GuaA,_GMP_synthase_-_Glutamine_amidotransferase_domain	2	2.1086	0.4107
2.01	2.01	13.9	contig00022_-_N5-carboxyaminoimidazole_ribonucleotide_mutase	1	0.5916	0.3905
2.01	2.01	8.7	contig00013_-_hscB,_co-chaperone_HscB	1	1.0471	0.8427
2.01	2.01	13.7	contig00007_-_bifunctional_pyrimidine_regulatory_protein_PyrR_uracil_phosphoribosyltransferase	1	0.5916	0.3084
2.01	2.01	7.3	contig00004_-_Dyp-type_peroxidase_family	1	0.8241	0.2704
2.01	2.01	9.6	contig00001_-_Predicted_transcriptional_regulator	1	4.8753	0.1535
2	17.88	41.7	contig00027_-_Gram-negative_porin	28	1.7701	0.3561
2	8	25	contig00005_-_Nitrogen_regulatory_protein_P-II	5	2.1086	0.47
2	4.01	8	contig00009_-_Hemolysin_secretion_protein	2	2.0137	0.3225
2	4	4.8	contig00005_-_Transcriptional_regulator	2	0.3162	0.2109
2	3.59	3.9	contig00001_-_aspartate_aminotransferase	2	0.2168	0.1609
2	2.09	4.7	contig00011_-_methylmalonic_acid_semialdehyde_dehydrogenase	2		
2	2.03	5.3	contig00005_-_LppC_putative_lipoprotein	2	0.3908	0.2545
2	2.03	7	contig00002_-_Predicted_3'-5'_exonuclease_related_to_the_exonuclease_domain_of_PolB	1	0.5012	0.3328
2	2.02	8.5	contig00015_-_ATP-dependent_RNA_helicase_RhlB	2		
2	2.02	9.6	contig00008_-_Cytochrome_C1_family	1		
2	2.01	15.1	contig00011_-_Vi_polysaccharide_biosynthesis_protein_TviB	1	11.0662	0.3063
2	2.01	9.8	contig00006_-_prolyl-tRNA_synthetase	1	0.7586	0.1616
2	2.01	7.9	contig00011_-_tryptophan_synthase_subunit_beta	1	1.3552	0.4641
2	2.01	14.3	contig00007_-_Competence_protein_A	1	1.6749	0.3756
2	2.01	6.8	contig00020_-_Curved_DNA-binding_protein	1	1.5996	0.4203
2	2.01	4.3	contig00007_-_Voltage_gated_chloride_channel	1	0.7047	0.5498
2	2.01	5.7	contig00011_-_Protein_CapI	1	0.2992	0.2172
2	2.01	8.4	contig00009_-_histidinol-phosphatase,_inositol_monophosphatase_family	1	7.656	0.3464
2	2.01	12.8	contig00003_-_helix_turn_helix,_Arsenical_Resistance_Operon_Repressor	1	0.8954	0.8232
2	2.01	10.8	contig00002_-_Carbamoyl-phosphate_synthase_small_chain,_CPSase_domain	1	0.8091	0.4021
2	2.01	13.3	contig00001_-_ihfA,_integration_host_factor_subunit_alpha	1	3.4995	0.178
2	2	7.2	contig00004_-_metG,_methionyl-tRNA_synthetase	1	0.6427	0.4707
2	2	8.7	contig00024_-_thiamine_biosynthesis_protein_ThiC	1	0.6427	0.4695

2	2	12.8	contig00005_-_sucC,_succinyl-CoA_synthetase_subunit_beta	1	0.0182	0.0965
2	2	14.5	contig00005_-_Bacterial_cellulose_synthase_subunit	1	0.7311	0.5819
2	2	32.8	contig00005_-_3-deoxy-D-manno-octulosonate_8-phosphate_phosphatase_KdsC	1	0.0337	0.0733
2	2	10.1	contig00003_-_membrane_protein_insertase	1	1.3183	0.6037
2	2	18.2	contig00002_-_RsuA,_16S_rRNA_uridine-516_pseudouridylate_synthase_and_related_pseudouridylate_synthases	1	0.0111	0.0583
2	2	11.3	contig00026_-_Trypsin-like_peptidase_domain	1	1.2942	0.5928
2	2	6.4	contig00026_-_Putative_transmembrane_protein_coupled_to_NADH-ubiquinone_oxidoreductase_chain	1	0.4406	0.2859
2	2	9.9	contig00022_-_S-adenosyl-L-homocysteine_hydrolase	1	0.2858	0.2139
2	2	18.5	contig00003_-_ispH,_4-hydroxy-3-methylbut-2-enyl_diphosphate_reductase	1	0.929	0.8848
2	2	11.6	contig00002_-_SseA,_Rhodanese-related_sulfurtransferase	1	0.0731	0.1462
2	2	24.8	contig00002_-_Dienelactone_hydrolase_family	1	0.6026	0.4586
2	2	8.5	contig00015_-_moaA,_molybdenum_cofactor_biosynthesis_protein_A	1	1.9588	0.3273
2	2	11.4	contig00013_-_Histidine--tRNA_ligase	1	0.4093	0.299
2	2	17.7	contig00011_-_Ttg2C,_ABC-type_transport_system_involved_in_resistance_to_organic_solvents,_periplasmic_component	1	3.3419	0.1988
2	2	9.4	contig00010_-_Probable_ATP-dependent_RNA_helicase_ddx27	2	3.1333	0.3475
2	2	13.1	contig00007_-_glutamate_synthase_small_subunit_family_protein,_proteobacterial	1	5.5463	0.1558
2	2	9.5	contig00007_-_Protein_PilJ	1	3.4041	0.4643
2	2	8.8	contig00006_-_phosphoribosylaminoimidazole_synthetase	1	1.1803	0.7307
2	2	13.6	contig00004_-_Glucokinase	1		
2	2	10.9	contig00003_-_Sulfide:quinone_oxidoreductase,_mitochondrial	1	0.15	0.1303
2	2	8.6	contig00001_-_isocitrate_dehydrogenase	1	0.2606	0.1819
2	2	11.1	contig00001_-_Cytochrome_c4	1	6.6069	0.1297
2	2	4.8	contig00027_-_TonB-dependent_siderophore_receptor	1		
2	2	28.1	contig00024_-_Predicted_transcriptional_regulator	1	0.0453	0.0804
2	2	6.5	contig00022_-_Carboxysome_Shell_Carbonic_Anhydrase	1	0.0429	0.127
2	2	13.6	contig00017_-_monothiol_glutaredoxin,_Grx4_family	1		
2	2	18.4	contig00016_-_PAS_sensor_protein	1	0.5395	0.337
2	2	6.9	contig00015_-_bifunctional_phosphoribosylaminoimidazolecarboxamide_formyltransferase/IMP_cyclohydrolase	1	1.5849	0.4393
2	2	25.3	contig00013_-_Chaperone_protein_HscA_homolog	1	1.1272	0.8965
2	2	7.8	contig00012_-_GIY-YIG_catalytic_domain	1	0.0394	0.2063
2	2	12.2	contig00011_-_trpA,_tryptophan_synthase_subunit_alpha	1	0.1803	0.1435
2	2	6.1	contig00010_-_phosphoenolpyruvate_carboxylase	1	0.3281	0.2902
2	2	9.8	contig00010_-_pgi,_glucose-6-phosphate_isomerase	1	0.0233	0.1347
2	2	6.2	contig00009_-_tyrosyl-tRNA_synthetase	1	0.0225	0.2271
2	2	8.5	contig00009_-_ZnuA,_ABC-type_Zn2+_transport_system,_periplasmic_component/surface_adhesin	2	8.091	0.177
2	2	19.8	contig00007_-_hisE,_phosphoribosyl-ATP_pyrophosphatase	1	2.5119	0.2538
2	2	6.4	contig00005_-_Type_4_fimbrial_assembly_protein_PilC	1	0.871	0.7952
2	2	9.4	contig00004_-_helix_turn_helix_multiple_antibiotic_resistance_protein	1	4.2073	0.473

2	2	13.3	contig00004_-_YdcF-like_protein	1	1.8365	0.3633
2	2	5.5	contig00004_-_LPS-assembly_protein_LptD	1	1.0186	0.645
2	2	24.2	contig00002_-_Signal_peptidase_I	2	1.5276	0.5394
2	2	10.4	contig00002_-_Predicted_kinase	1	1.5136	0.4728
2	2	5.2	contig00002_-_N-ethylammeline_chlorohydrolase	1	1.028	0.7152
2	2	11.7	contig00002_-_FHA_domain	4	3.1333	0.3125
2	2	16.7	contig00002_-_Escherichia_coli_YhbY_is_associated_with_pre-50S_ribosomal_subunits	1	0.7943	0.7195
2	2	16.3	contig00001_-_lipoprotein_releasing_system,_ATP-binding_protein	1	0.9638	0.6269
2	2	6.5	contig00001_-_ferrochelatase	1	0.052	0.1049
2	2	24.3	contig00036_-_Cysteine_desulfhydrase_/_Selenocysteine_lyase	1	0.9817	0.9871
2	2	6.5	contig00022_-_RuBisCO_operon_transcriptional_regulator	1	1.2589	0.6465
2	2	4.8	contig00020_-_periplasmic_serine_protease,_Do/DeqQ_family	1	1.7701	0.3789
2	2	12.3	contig00017_-_Thioredoxin_domain-containing_protein	1	1.4191	0.5276
2	2	10.4	contig00015_-_acetyl-CoA_carboxylase_biotin_carboxylase_subunit	1	0.9376	0.9047
2	2	12.2	contig00015_-_WcaG,_Nucleoside-diphosphate-sugar_epimerases	1	0.3981	0.2592
2	2	6.9	contig00012_-_GntR,_Transcriptional_regulators	1	0.2938	0.1983
2	2	19	contig00012_-_Aspartyl/glutamyl-tRNA(Asn/Gln)_amidotransferase_subunit_C	1	0.8091	0.7979
2	2	7.6	contig00011_-_chorismate_synthase	1	0.4786	0.3196
2	2	3.8	contig00011_-_amidophosphoribosyltransferase	1		
2	2	17.7	contig00010_-_ribH,_6,7-dimethyl-8-ribityllumazine_synthase	1	0.0955	0.1595
2	2	11.3	contig00010_-_Oxidoreductase,_aldo/keto_reductase_family	1	1.4454	0.2014
2	2	4.1	contig00010_-_Outer_membrane_protein_transport_protein	1	3.1915	0.206
2	2	6.7	contig00010_-_NADP-dependent_L-serine/L-allo-threonine_dehydrogenase_YdfG	1	0.5808	0.4002
2	2	8.3	contig00009_-_glycine_cleavage_system_protein_H	2	0.955	0.8251
2	2	11.7	contig00009_-_Vitamin_B12_dependent_methionine_synthase,_activation_domain	1	0.5649	0.3876
2	2	9.8	contig00009_-_Predicted_proteasome-type_protease	2	0.138	0.2551
2	2	13.8	contig00009_-_Phosphoesterase_family	1	1.3677	0.5776
2	2	2.2	contig00008_-_Periplasmic_binding_protein	1	0.0398	0.0846
2	2	7.9	contig00007_-_tRNA_threonylcarbamoyladenosine_biosynthesis_protein_RimN	1	0.1306	0.1215
2	2	11.3	contig00007_-_ATPase_components_of_ABC_transporters_with_duplicated_ATPase_domains	1	0.6982	0.5369
2	2	28.2	contig00007_-_30S_ribosomal_protein_S21	1	0.863	0.4226
2	2	11.4	contig00007_-_3'(2'),5'-bisphosphate_nucleotidase,_bacterial	1	0.3105	0.241
2	2	6.3	contig00006_-_PhoH,_Phosphate_starvation-inducible_protein_PhoH,_predicted_ATPase	1	0.3467	0.2567
2	2	10.2	contig00006_-_Glycine-zipper_containing_OmpA-like_membrane_domain	2	1.1066	0.8518
2	2	4.4	contig00006_-_Flagellin_protein	1	8.9536	0.1498
2	2	6.4	contig00006_-_Chaperone_protein_HtpG	1	1.1482	0.7727
2	2	5.4	contig00005_-_ddl,_D-alanine--D-alanine_ligase	1		
2	2	4.5	contig00005_-_UDP-N-acetylglucosamine_1-carboxyvinyltransferase	1		
2	2	10.4	contig00005_-_TolQ_protein	1	10	0.277

2	2	5.1	contig00005_-_Cell_division_protein_FtsA	1	2.704	0.2369
2	2	23	contig00004_-_TolA_protein	2	1.1169	0.9841
2	2	19.3	contig00004_-_PspE,_Rhodanese-related_sulfurtransferase	1	0.6546	0.4809
2	2	5.8	contig00004_-_Enolase-phosphatase_E1	1	0.7447	0.6036
2	2	3.4	contig00003_-_argininosuccinate_lyase	1	1.1482	0.77
2	2	7.6	contig00003_-_Bacterial_protein_of_unknown_function_(DUF945)	1	1.7539	0.3835
2	2	4.7	contig00003_-_6-phosphofructokinase	1	1.3183	0.6003
2	2	5.7	contig00002_-_clpP,_ATP-dependent_Clp_protease_proteolytic_subunit	1	0.4246	0.3635
2	2	5.3	contig00002_-_Uncharacterized_oxidoreductase_YfjR	1	0.4875	0.688
2	2	10.2	contig00002_-_FabI,_Enoyl-[acyl-carrier-protein]	1	2.1086	0.3031
2	2	6.1	contig00002_-_3-phosphoshikimate_1-carboxyvinyltransferase	1	0.52	0.3474
2	2	9	contig00001_-_tmk,_thymidylate_kinase	1	0.2655	0.1843
2	2	12	contig00001_-_pyrH,_uridylate_kinase	1	0.5445	0.367
2	2	4.6	contig00001_-_3-oxoacyl-(acyl_carrier_protein)_synthase_III	1	0.4055	0.2634
2	2	9.4	contig00027_-_Thioredoxin-like_protein	2	2.7797	0.2397
2	2	3.4	contig00027_-_Tas,_Predicted_oxidoreductases	1	0.5495	0.3726
2	2	11.1	contig00025_-_AmpD_protein	1	1.5417	0.3892
2	2	2.9	contig00024_-_rfaD,_ADP-L-glycero-D-mannoheptose-6-epimerase	1		
2	2	3.5	contig00022_-_Probable_transcriptional_regulator_LumQ	1	0.787	0.6611
2	2	2.5	contig00018_-_Outer_membrane_protein_beta-barrel_domain	1	2.3988	0.4176
2	2	5.2	contig00017_-_Chemotaxis_protein_CheY	1	0.3565	0.2336
2	2	5.4	contig00016_-_gmk,_guanylate_kinase	1	14.0605	0.2206
2	2	1.6	contig00016_-_dihydrolipoamide_succinyltransferase	1	0.492	0.3257
2	2	3.6	contig00016_-_Sterol-binding_domain_protein	1	3.02	0.3931
2	2	21.4	contig00015_-_CopZ,_Copper_chaperone	1	2.704	0.23
2	2	3.6	contig00014_-_Hydrogen_peroxide-inducible_genes_activator	1	0.6026	0.4332
2	2	5.5	contig00013_-_RNA_polymerase_sigma-70_factor,_TIGR02943_family	1	0.8318	0.7307
2	2	5	contig00012_-_upp,_uracil_phosphoribosyltransferase	1		
2	2	4.9	contig00012_-_Uncharacterized_Fe-S_protein	1	1.4997	0.4798
2	2	5.2	contig00012_-_SlpA,_FKBP-type_peptidyl-prolyl_cis-trans_isomerases_2	3	1.3804	0.3461
2	2	2	contig00011_-_beta_alanine--pyruvate_transaminase	1		
2	2	4.4	contig00011_-_Peptidase_C26	1	0.6026	0.4231
2	2	7.2	contig00011_-_ABC-type_uncharacterized_transport_system,_auxiliary_component	1	1.2359	0.6769
2	2	5.3	contig00010_-_heavy_metal_response_regulator	1	1.6144	0.4315
2	2	14	contig00010_-_YciI-like_protein	1		
2	2	11.8	contig00009_-_iron-sulfur_cluster_insertion_protein_ErpA	2	2.2284	0.2865
2	2	8.1	contig00009_-_Rhodanese_Homology_Domain	1	1.3804	0.6178
2	2	5.3	contig00009_-_NAD(P)H:quinone_oxidoreductase	1	0.4656	0.2621
2	2	6.1	contig00008_-_Transcriptional_regulator_containing_an_amidase_domain_and_an_AraC-type_DNA-	1	0.0871	0.1016

			binding_HTH_domain			
2	2	10.2	contig00007_-_Hemerythrin_HHE_cation_binding_domain	2	1.1272	0.8455
2	2	6.3	contig00007_-_Cyclic_nucleotide-binding_domain	1	0.6026	0.4208
2	2	3.5	contig00006_-_secY,_preprotein_translocase_subunit_SecY	1	0.0817	0.1501
2	2	4.6	contig00006_-_LPS-assembly_lipoprotein_LptE	1	0.3076	0.2063
2	2	7.9	contig00005_-_Sigma_54_modulation_protein_/_S30EA_ribosomal_protein	1	3.6983	0.2039
2	2	4	contig00005_-_Probable_3-mercaptopyruvate_sulfurtransferase	1	0.3467	0.2273
2	2	7.2	contig00005_-_Predicted_peroxiredoxins	1	0.302	0.3762
2	2	7.6	contig00004_-_fabG,_3-ketoacyl-(acyl-carrier-protein)_reductase	1	0.0457	0.0807
2	2	2.4	contig00004_-_Tetratricopeptide_TPR_4	1	1.4322	0.5218
2	2	8.6	contig00004_-_Lipoate_regulatory_protein_YbeD	1	0.6252	0.4499
2	2	7.5	contig00004_-_Ferredoxin_1	1	0.7178	0.5964
2	2	3	contig00002_-_cmk,_cytidylate_kinase	1	1.406	0.5357
2	2	4.7	contig00002_-_Ureidoglycolate_lyase	1	0.6668	0.9357
2	2	9.1	contig00002_-_Protein_YciI	1	1.1169	0.8227
2	2	21.7	contig00002_-_Protein-export_membrane_protein_SecG	1	4.7424	0.3289
2	2	3.4	contig00002_-_NADPH_dehydrogenase_NamA	1	0.0738	0.2339
2	2	9.7	contig00001_-_ubiquinol-cytochrome_c_reductase,_iron-sulfur_subunit	1	1.3677	0.5615
2	2	5.4	contig00001_-_ompR,_osmolarity_response_regulator	1		
2	2	4.9	contig00001_-_UDP-N-acetylglucosamine_acyltransferase	1	0.6546	0.4789
2	2	18	contig00001_-_Acyl_carrier_protein	1	1.556	0.4542
2	2	4.7	contig00001_-_5'-methylthioadenosine_phosphorylase	1		
1.92	2	10.9	contig00007_-_Putative_cell_wall_biogenesis_protein	1	1.8535	0.3145
1.92	2	11.4	contig00002_-_Scavenger_mRNA_decapping_enzyme_C-term_binding	1	0.8472	0.4192
1.92	2	4.4	contig00002_-_PTC1,_Serine/threonine_protein_phosphatase	1	0.3945	0.2569
1.92	2	4.7	contig00002_-_NADH-quinone_oxidoreductase_subunit_J	1	1.9588	0.5318
1.82	2	14.3	contig00002_-_PilZ_domain	1	0.5058	0.335
1.74	2	9.2	contig00012_-_gatA,_aspartyl/glutamyl-tRNA_amidotransferase_subunit_A	1	1.2942	0.693
1.74	2	2	contig00011_-_adenylosuccinate_synthetase	1	1.6596	0.4149
1.68	1.68	13.5	contig00014_-_ilvH,_acetolactate_synthase_3_regulatory_subunit	1	0.6546	0.5193
1.68	1.68	6.3	contig00013_-_lysS,_lysyl-tRNA_synthetase	1	0.5346	0.6329
1.6	1.6	9.7	contig00003_-_branched-chain_amino_acid_aminotransferase	1	0.7516	0.6114
1.57	1.57	8.3	contig00006_-_RfbB,_dTDP-D-glucose_4,6-dehydratase	1	0.3133	0.2089
1.57	1.57	17.7	contig00026_-_Ferredoxin,_2Fe-2S	1	1.0864	0.85
1.52	1.52	1.7	contig00024_-_bifunctional_heptose_7-phosphate_kinase/heptose_1-phosphate_adenyltransferase	1	0.9638	0.7537
1.48	1.48	6.8	contig00017_-_Carboxymuconolactone_decarboxylase	1	1.3932	0.5471
1.41	1.41	12.9	contig00003_-_GTPase_obg	1	1.2823	0.631

Unused	Total	% Cov	Accession #	Peptides (95%)	117:114	PVal 117:114
97.08	97.08	60	contig00015_-_60_kDa_chaperonin	107	2.3335	0.0001
68.35	68.35	46.4	contig00002_-_NADH_dehydrogenase	48	0.5152	0.6686
47.72	47.72	73.3	contig00004_-_outer_membrane_protein_A	44	0.0363	0
45.99	45.99	62.8	contig00006_-_ectoine/hydroxyectoine_ABC_transporter_solute-binding_protein	44	51.5229	0.0001
43.59	43.59	39.8	contig00022_-_Carboxysome_shell_peptide_mid-region	42	0.1614	0.0892
41.87	41.87	51.3	contig00002_-_dnaK,_molecular_chaperone_DnaK	33	5.9704	0
39.73	39.73	20.3	contig00011_-_FimV,_Tfp_pilus_assembly_protein_FimV	26	2.9648	0.0366
38.78	38.78	37.1	contig00008_-_Putative_outer_membrane_cytochrome_c	50	7.4473	0.029
36.97	37.02	30.4	contig00016_-_Protein_AsmA	25	0.1127	0
36.27	36.27	41.2	contig00022_-_fructose-1,6-bisphosphate_aldolase	29	0.0535	0.0001
35.75	35.75	83.2	contig00026_-_Major_outer_membrane_protein_P.IB	66	4.7424	0.0103
34.54	34.58	61.5	contig00008_-_Rusticyanin_protein	52	15.7036	0.0001
32.43	32.43	62.1	contig00006_-_Rubrerythrin_protein	66	0.7586	0.8392
31.82	31.82	52.7	contig00044_-_elongation_factor_Tu	33	0.2399	0.1306
31.17	32.54	35	contig00002_-_rpsA,_30S_ribosomal_protein_S1	19	1.3932	0.7114
30.92	30.92	37.6	contig00022_-_Ribulose_bisphosphate_carboxylase_large_chain	26	0.3698	0.0028
30.46	30.46	43.1	contig00002_-_Probable_parvulin-type_peptidyl-prolyl_cis-trans_isomerase	24	2.8314	0.0003
28.36	28.36	16.1	contig00030_-_DNA-directed_RNA_polymerase_subunit_beta	16	0.955	0.5027
27.76	27.76	50.8	contig00003_-_ATP_synthase_subunit_alpha	20	0.2443	0.0001
27.28	27.28	23.7	contig00030_-_elongation_factor_G	16	0.863	0.919
26.72	26.72	43.9	contig00003_-_phosphoribulokinase	20	0.1169	0.0014
26.61	26.61	29.8	contig00003_-_F0F1_ATP_synthase_subunit_beta	22	0.4285	0.1935
26.12	26.12	47.3	contig00001_-_2,3,4,5-tetrahydropyridine-2,6-dicarboxylate_N-succinyltransferase	16	6.4863	0
25.88	25.88	39	contig00003_-_glnA,_glutamine_synthetase	21	0.182	0.0223
25.61	25.61	33.7	contig00004_-_mdoG,_glucan_biosynthesis_protein_G	16	0.1432	0.0082
24.06	24.06	58.4	contig00006_-_rplB,_50S_ribosomal_protein_L2	15	0.2249	0.0177
23.91	23.91	36.8	contig00008_-_Carbohydrate-selective_porin,_OprB_family	19	0.2333	0.0057
23.78	24.31	40.4	contig00006_-_DNA-directed_RNA_polymerase_subunit_alpha	17	0.1028	0
23.71	23.71	57.6	contig00006_-_rplF,_50S_ribosomal_protein_L6	20	3.3419	0.1359
23.4	23.4	30.2	contig00025_-_BtuB,_Outer_membrane_cobalamin_receptor_protein	19	6.5464	0.0017
23.3	23.3	60.1	contig00010_-_30S_ribosomal_protein_S6	15	2.5823	0.0009
23.27	23.27	37.9	contig00002_-_Trigger_factor	25	0.413	0.0591
22.46	22.46	61.5	contig00056_-_Gram-negative_porin	47	5.1523	0.0046
22.23	22.23	62.5	contig00015_-_10_kDa_chaperonin	23	9.4624	0.0003
22.03	22.03	27.1	contig00003_-_Prc,_Periplasmic_protease	14	0.2421	0.0719
21.96	21.96	73.3	contig00066_-_Major_carboxysome_shell_protein_1A	38	0.0511	0.0448
21.56	21.59	45.3	contig00004_-_HdrA,_Heterodisulfide_reductase,_subunit_A_and_related_polyferredoxins	14	2.1086	0.3354

21.35	21.35	66.5	contig00001_-_frr,_ribosome_recycling_factor	15	4.2073	0
20.56	20.56	48	contig00002_-_signal_peptidase_I	14	7.8705	0.0029
20.36	20.36	35.6	contig00004_-_Chaperone_SurA	11	3.2509	0.1314
19.75	21.77	29.9	contig00013_-_eno,_enolase	15	0.182	0.0095
19.7	19.7	30.4	contig00022_-_pyruvate_kinase	17	1.2589	0.5686
17.76	17.76	18.1	contig00001_-_polynucleotide_phosphorylase/polyadenylase	9	0.4742	0.0803
17.71	17.71	28.4	contig00003_-_Periplasmic_binding_protein	16	5.4954	0.0042
17.6	17.6	39.5	contig00006_-_LysM_domain/BON_superfamily_protein	34	1.5849	0.2867
17.35	17.35	53.5	contig00001_-_tsf,_elongation_factor_Ts	14	2.1281	0.0361
16.81	16.81	37.4	contig00012_-_50S_ribosomal_protein_L25/general_stress_protein_Ctc	15	1.9231	0.1198
16.77	17.73	20.7	contig00017_-_UshA,_5'-nucleotidase/2',3'-cyclic_phosphodiesterase_and_related_esterases	14	0.0145	0
16.62	16.62	29.6	contig00022_-_Glyceraldehyde-3-phosphate_dehydrogenase	11	0.3192	0.0261
16.59	16.59	20	contig00014_-_multifunctional_aminopeptidase_A	12	0.0363	0.0014
16.05	16.05	41.1	contig00008_-_hypothetical_protein	12	8.4723	0.0011
16	16	48	contig00030_-_50S_ribosomal_protein_L7/L12	13	5.1523	0.0017
16	16	42.1	contig00013_-_Transmembrane_protein	9	3.4674	0.0183
16	16	23.3	contig00027_-_Dienelactone_hydrolase_and_related_enzymes	12	1.8535	0.1376
15.72	15.72	57.1	contig00001_-_RNA_polymerase-binding_transcription_factor_DksA	10	3.6983	0.0046
15.33	15.33	57.7	contig00030_-_30S_ribosomal_protein_S7	13	0.1738	0.0614
15.33	15.33	34	contig00003_-_preprotein_translocase_subunit_SecB	9	3.9084	0.0376
15.27	15.27	12.5	contig00001_-_Phosphoesterase_family	9	0.929	0.5719
15.27	15.27	15.6	contig00021_-_Protein_translocase_subunit_SecD	8	0.5297	0.0486
14.98	14.98	67.1	contig00016_-_SirA-like_protein	14	1.6904	0.0608
14.88	14.88	22.1	contig00002_-_periplasmic_folding_chaperone	8	0.0506	0.0016
14.7	14.7	30.8	contig00004_-_tolB,_translocation_protein_TolB	14	2.8314	0.0088
14.68	14.68	33.1	contig00014_-_ketol-acid_reductoisomerase	11	0.2831	0.1232
14.63	14.71	38.3	contig00030_-_50S_ribosomal_protein_L10	9	0.1644	0.0078
14.28	14.28	11.2	contig00001_-_aconitate_hydratase	8	0.4446	0.555
14.13	14.13	56.7	contig00009_-_CspC,_Cold_shock_proteins	11	1.2474	0.8262
14.02	14.02	29.1	contig00003_-_F0F1_ATP_synthase_subunit_alpha	10	0.8017	0.5479
14	14.03	23.5	contig00022_-_Phosphoglycerate_kinase	9	0.047	0.055
14	14	15.3	contig00024_-_Porins_form_aqueous_channels_for_the_diffusion_of_small_hydrophillic_molecules_across_the_outer_membrane	8	2.7797	0.0049
14	14	48.1	contig00016_-_oxidative_damage_protection_protein	16	0.9462	0.3414
14	14	39.3	contig00001_-_Translation_initiation_factor_IF-3	8	0.0384	0
13.72	13.72	50.5	contig00004_-_Sulphur_oxidation_protein_SoxZ	16	2.7542	0.0031
13.6	15.69	22	contig00004_-_DacC,_D-alanyl-D-alanine_carboxypeptidase	9	0.2704	0.3052
13.6	13.6	23.4	contig00007_-_LysM_domain/BON_superfamily_protein	7	0.5012	0.0887
13.55	13.55	71.6	contig00006_-_50S_ribosomal_protein_L29	9	4.8306	0.0039

13.44	14.82	37.2	contig00001_-_RND_family_efflux_transporter,_MFP_subunit	11	0.0586	0.0194
13.32	13.32	22.3	contig00004_-_Multidrug_resistance_outer_membrane_protein_MdtP	7	0.5248	0.0941
13.19	13.19	34.1	contig00006_-_rplC,_50S_ribosomal_protein_L3	8	0.871	0.6021
13.15	13.15	36.5	contig00007_-_putative_outer_membrane_lipoprotein	11	2.1677	0.2129
13.08	13.08	21.1	contig00036_-_cysteine_desulfurase	7	1.9588	0.1386
12.83	12.83	48.4	contig00002_-_bacterial_(prokaryotic)_histone_like_domain	18	3.5645	0.2632
12.74	12.74	26.3	contig00004_-_tol-pal_system_protein_YbgF	11	4.2073	0.006
12.69	12.69	22.8	contig00003_-_fructose-1,6-bisphosphatase	9	0.0738	0.0113
12.47	12.47	31.9	contig00002_-_greA,_transcription_elongation_factor_GreA	8	4.1305	0.6316
12.39	12.39	42.7	contig00007_-_Protein_PilG	7	8.8716	0.0034
12.33	12.33	22.1	contig00004_-_HdrB,_Heterodisulfide_reductase,_subunit_B	12	0.3981	0.2113
12.23	12.25	39.9	contig00005_-_Toluene_tolerance,_Ttg2	9	15.2757	0.0002
12.12	12.12	15.8	contig00002_-_ATP-dependent_zinc_metalloprotease_FtsH	7	2.0137	0.0353
12.11	12.11	41.1	contig00004_-_Acireductone_dioxygenase	7	0.2965	0.0154
12.06	12.06	21.1	contig00007_-_Phosphate-binding_protein_PstS_1	16	0.0406	0.0001
12.05	12.05	29.1	contig00008_-_CyoA,_Heme/copper-type_cytochrome/quinol_oxidases,_subunit_2	10	8.0168	0.0733
12.04	12.04	43.3	contig00017_-_Putative_lipoprotein	9	3.2509	0.0155
12	12	29.3	contig00017_-_transcriptional_regulator_PhoU	8	0.2228	0.319
12	12	37.2	contig00005_-_Putative_peptidoglycan_binding_domain	8	0.0213	0.0022
12	12	27.3	contig00030_-_50S_ribosomal_protein_L11	6	2.1677	0.2282
11.86	11.86	28	contig00004_-_peptidoglycan-associated_lipoprotein	21	2.2909	0.076
11.75	11.75	14.9	contig00012_-_hypothetical_protein	6	0.0421	0.02
11.73	11.73	42.6	contig00006_-_rpsE,_30S_ribosomal_protein_S5	8	0.5649	0.0837
11.64	11.64	47.3	contig00006_-_rplE,_50S_ribosomal_protein_L5	7	0.0661	0.2457
11.45	11.45	21.8	contig00010_-_glyA,_serine_hydroxymethyltransferase	7	0.1148	0.1212
11.37	11.37	22.7	contig00011_-_HflK_protein	8	2.0137	0.1351
11.18	11.43	16	contig00005_-_ResB-like_family	9	0.6855	0.6575
11.05	11.05	26.7	contig00006_-_rpsD,_30S_ribosomal_protein_S4	6	0.7586	0.3604
11.02	11.02	36.9	contig00015_-_Thioredoxin_protein	9	3.02	0.0124
10.97	10.97	18	contig00005_-_Thiazole_synthase	8	2.3988	0.3527
10.73	10.73	20.1	contig00002_-_GalU,_UDP-glucose_pyrophosphorylase	5	0.1486	0.0117
10.71	10.85	22.1	contig00001_-_Ubiquinol_oxidase_subunit_2	9	4.5709	0.0853
10.7	10.7	28.6	contig00006_-_Uncharacterized_enzyme_involved_in_biosynthesis_of_extracellular_polysaccharides	12	2.9107	0.0808
10.49	10.49	12.3	contig00001_-_MrcA,_Membrane_carboxypeptidase/penicillin-binding_protein	8	0.0421	0.0095
10.34	10.34	21.9	contig00001_-_6-phosphogluconate_dehydrogenase	6	0.0156	0.0046
10.34	10.34	67.4	contig00016_-_DNA-directed_RNA_polymerase_subunit_omega	8	0.3373	0.1084
10.13	10.13	24.9	contig00002_-_Universally_conserved_protein	8	4.5709	0.0032
10.09	10.09	32.6	contig00001_-_rpsB,_30S_ribosomal_protein_S2	9	0.169	0.1897
10.07	10.07	61.2	contig00006_-_rplW,_50S_ribosomal_protein_L23	6	0.0817	0.0095

10.02	10.02	46.6	contig00006_-_rpsM,_30S_ribosomal_protein_S13	6	0.0625	0.0087
10.02	10.02	21.7	contig00016_-_multidrug_efflux_system_protein_EmrA	7	0.0946	0.0093
10.02	10.02	55.7	contig00001_-_3-ketoacyl-(Acyl-carrier-protein)_reductase	7	4.6989	0.0057
10	10	24.2	contig00030_-_rplA,_50S_ribosomal_protein_L1	6	0.9036	0.7023
10	10	20.1	contig00013_-_Cytoskeleton_protein_RodZ	5	7.5858	0.0104
10	10	64.8	contig00006_-_rplX,_50S_ribosomal_protein_L24	8	0.9908	0.6142
10	10	53.3	contig00002_-_heat_shock_protein_GrpE	7	5.3951	0.0131
10	10	32.9	contig00013_-_ndk,_mulitfunctional_nucleoside_diphosphate_kinase/apyrimidinic_endonuclease/3	10	2.9376	0.0158
10	10	16.3	contig00003_-_Probable_chromosome-partitioning_protein_ParB	6	0.182	0.018
9.91	9.91	20.9	contig00012_-_RND_family_efflux_transporter,_MFP_subunit	8	0.3908	0.8334
9.75	9.75	43.2	contig00006_-_rpsH,_30S_ribosomal_protein_S8	8	0.9817	0.6696
9.5	9.58	21.1	contig00011_-_HflC_protein	5	0.2911	0.1586
9.45	9.45	52.8	contig00022_-_Ribulose_bisphosphate_carboxylase_small_chain	12	0.2228	0.0377
9.28	9.28	41.5	contig00006_-_30S_ribosomal_protein_S11	8	5.8614	0.207
9.13	9.13	54.1	contig00014_-_LysR,_Transcriptional_regulator	6	0.2729	0.1781
8.86	8.92	24.1	contig00019_-_Outer_membrane_beta-barrel_domain_protein	7	8.4723	0.1359
8.85	8.85	28.5	contig00010_-_50S_ribosomal_protein_L9	6	2.4889	0.0761
8.71	8.71	14.6	contig00025_-_5-methyltetrahydropteroyltriglutamate--_homocysteine_S-methyltransferase	6	0.8872	0.8383
8.54	8.54	33.9	contig00006_-_single-stranded_DNA-binding_protein	5	5.3951	0.0238
8.53	8.53	25.4	contig00016_-_SoxAX_cytochrome_complex_subunit_A	5	0.0855	0.0195
8.52	8.52	15.1	contig00016_-_tolC,_outer_membrane_channel_protein	10	1.8365	0.2049
8.49	8.49	24	contig00014_-_Chemotaxis_phosphatase,_CheZ	6	3.3113	0.0239
8.45	10.79	15.4	contig00006_-_Outer_membrane_protein_assembly_factor_BamC	15	1.8197	0.4253
8.4	8.4	15.5	contig00008_-_signal_peptide_peptidase_SppA,_67K_type	4	0.0738	0.0538
8.34	8.34	17.4	contig00004_-_recA,_recombinase_A	6	0.1419	0.0348
8.26	8.26	8.8	contig00006_-_Zn-finger_containing_NTP_pyrophosphohydrolase	4	1.6444	0.7129
8.25	8.25	37.3	contig00001_-_hypothetical_protein	5	1.1272	0.9361
8.19	8.19	33.9	contig00010_-_Membrane_protein_involved_in_aromatic_hydrocarbon_degradation	22	3.4995	0.087
8.15	8.15	55.8	contig00006_-_rpsJ,_30S_ribosomal_protein_S10	5	0.8395	0.8645
8.14	8.14	32.6	contig00009_-_tpx,_lipid_hydroperoxide_peroxidase	7	0.7244	0.5324
8.12	8.12	27.7	contig00001_-_Predicted_peroxiredoxins	7	0.6668	0.5035
8.11	8.11	27.6	contig00004_-_transaldolase	6	0.8395	0.4109
8.08	8.08	18.9	contig00026_-_Protein_CbbQ	5	1.5276	0.664
8.06	8.06	51.2	contig00001_-_NADPH-dependent_7-cyano-7-deazaguanine_reductase	4	1.3305	0.3981
8.05	8.05	42.2	contig00007_-_Protein_PilH	5	2.5823	0.0974
8.04	8.04	18.4	contig00006_-_RND_family_efflux_transporter,_MFP_subunit	5	0.5754	0.0563
8.03	8.03	25.9	contig00008_-_SCO1/SenC_protein	4	0.207	0.0021
8.01	8.03	7.5	contig00005_-_Multidrug_resistance_protein_MdtB	4	0.1213	0.0322
8.01	8.01	19.9	contig00001_-_FabD,_(acyl-carrier-protein)_S-malonyltransferase	6	0.3162	0.1794

8	10	15.1	contig00012_-_HDOD_domain	5	0.0565	0.0929
8	10	23.9	contig00008_-_GlnK,_Nitrogen_regulatory_protein_PII	5		
8	8	18	contig00009_-_gcvT,_glycine_cleavage_system_aminomethyltransferase_T	4	0.0581	0.009
8	8	24.8	contig00009_-_Conserved_hypothethical_protein,_UCP019302	4	3.9811	0.0235
8	8	26	contig00017_-_thioredoxin_protein	6	2.355	0.191
8	8	32.3	contig00016_-_reactive_intermediate/imine_deaminase	6	8.1658	0.0111
8	8	23.6	contig00006_-_30S_ribosomal_protein_S14	4	0.2704	0.3721
8	8	18.5	contig00005_-_outer_membrane_assembly_lipoprotein_YfiO	4	1.3552	0.6239
8	8	23.1	contig00003_-_ATP_synthase_subunit_b	5	1.9409	0.1352
7.92	7.92	38.8	contig00005_-_Ttg2C,_ABC-type_transport_system_involved_in_resistance_to_organic_solvents	4	1.2589	0.2041
7.89	7.89	37.2	contig00022_-_carboxysome_peptide_B	4	1.2706	0.6863
7.79	7.79	13.9	contig00002_-_nusA,_transcription_elongation_factor_NusA	4	1.6904	0.5207
7.62	7.62	12.2	contig00005_-_CirA,_Outer_membrane_receptor_proteins,_mostly_Fe_transport	5	1.1588	0.5966
7.6	7.6	15	contig00017_-_dihydrolipoamide_acetyltransferase	5	1.2023	0.7567
7.48	7.48	25.5	contig00018_-_Gram-negative_porin	7	0.8395	0.7894
7.38	7.38	13.7	contig00002_-_acetyl-CoA_synthetase	4	0.8091	0.9133
7.13	7.13	13.8	contig00005_-_Fimbrial_protein_P9-2	11	3.1046	0.1571
7.07	7.07	24.1	contig00004_-_ferredoxin-NADP_reductase	4	1.1169	0.3868
7.03	9.31	17.8	contig00003_-_DNA_polymerase_III_subunit_beta	6	1	0.7519
6.95	6.95	39.1	contig00006_-_rpsC,_30S_ribosomal_protein_S3	5	0.1067	0.0124
6.88	6.88	20.9	contig00026_-_ribulose-phosphate_3-epimerase	4	0.9638	0.5607
6.86	6.86	19.4	contig00007_-_Fimbrial_assembly_protein_PilQ	4	1.8707	0.3783
6.84	6.84	16.9	contig00003_-_pntA,_NAD(P)_transhydrogenase_subunit_alpha	4	0.955	0.922
6.77	6.77	14.5	contig00003_-_HemX,_Uncharacterized_enzyme_of_heme_biosynthesis	7	3.8371	0.0328
6.68	6.68	31.9	contig00021_-_yajC,_preprotein_translocase_subunit_YajC	5	1.5276	0.6314
6.57	6.57	34.2	contig00006_-_Cytochrome_c	5	0.9817	0.9912
6.45	6.45	36.3	contig00030_-_rpsL,_30S_ribosomal_protein_S12	5	0.8872	0.5215
6.24	6.24	18.3	contig00003_-_LivK,_ABC-type_branched-chain_amino_acid_transport_systems,_periplasmic_component	4	0.1096	0.0213
6.22	6.22	27.8	contig00005_-_phosphoheptose_isomerase	7	0.5861	0.1383
6.18	6.18	13.6	contig00015_-_rho,_transcription_termination_factor_Rho	3	0.9817	0.9218
6.13	6.13	32.1	contig00005_-_Thiol:disulfide_interchange_protein_DsbA	3	4.3251	0.0296
6.13	6.13	20.6	contig00006_-_Fe-S_oxidoreductase-like_protein	3	0.3802	0.1548
6.08	6.18	17	contig00008_-_AfuA,_ABC-type_Fe3+_transport_system,_periplasmic_component	6	0.787	0.4519
6.06	6.06	23	contig00012_-_YecA_family_protein	5	0.4169	0.144
6.03	6.03	14.2	contig00008_-_Outer_membrane_lipoprotein_Slp_family	4	1.2942	0.4429
6.03	6.03	13.8	contig00002_-_Aminoglycoside_phosphotransferase	3	0.2377	0.1349
6.02	6.02	14.1	contig00001_-_rne,_ribonuclease_E	3	3.281	0.1972
6.02	6.02	27.5	contig00002_-_elongation_factor_P	3	1.2023	0.2805
6.01	6.02	32.7	contig00006_-_ATPase_with_chaperone_activity,_ATP-binding_subunit	3	3.8371	0.2106

6.01	6.01	17.3	contig00012_-_gatB,_aspartyl/glutamyl-tRNA_amidotransferase_subunit_B	3	0.9817	0.9801
6.01	6.01	22.9	contig00010_-_The_CBS_domain,_is_a_small_domain_originally_identified_in_cystathionine_beta-synthase	4	0.0356	0.0463
6.01	6.01	13.3	contig00008_-_Cytochrome_c4	4	1.5704	0.3055
6.01	6.01	43.6	contig00020_-_Bcp,_Peroxiredoxin	3	1.5276	0.6307
6.01	6.01	14	contig00015_-_Disulphide_bond_corrector_protein_DsbC	5	2.421	0.3395
6.01	6.01	23.9	contig00011_-_FtsH_protease_regulator_HflC	5	1.0864	0.9904
6.01	6.01	29.1	contig00016_-_DsrE/DsrF-like_family	7	0.3532	0.1063
6	6.59	33.7	contig00002_-_SirA-like_protein	3	0.929	0.4046
6	6.09	12.7	contig00017_-_aceE,_pyruvate_dehydrogenase_subunit_E1	3	0.3873	0.214
6	6.01	23.6	contig00003_-_OmpR,_Response_regulators_consisting_of_a_CheY-like_receiver_domain_and_a_winged-helix_DNA-binding_domain	3	0.912	0.6659
6	6.01	11.9	contig00013_-_outer_membrane_assembly_lipoprotein_YfgL	7	1.7219	0.1958
6	6.01	12.1	contig00022_-_transketolase	5	0.52	0.0779
6	6.01	42.2	contig00006_-_rpsS,_30S_ribosomal_protein_S19	6	1.1803	0.3046
6	6.01	9.3	contig00003_-_Tas,_Predicted_oxidoreductases	3	0.0832	0.0416
6	6	25.8	contig00010_-_secreted_protein	3	5.4954	0.0811
6	6	6	contig00009_-_Protein_HelA	3	0.9727	0.2165
6	6	41.2	contig00006_-_rplV,_50S_ribosomal_protein_L22	5	0.1038	0.0499
6	6	26.9	contig00001_-_Elongation_factor_Ts	4	2.729	0.1876
6	6	21	contig00026_-_Predicted_redox_protein,_regulator_of_disulfide_bond_formation	4	4.2855	0.0547
6	6	10.2	contig00017_-_hypothetical_protein	3	1.5996	0.5067
6	6	17.8	contig00012_-_Cytochrome_C	3	0.056	0.1421
6	6	12.2	contig00004_-_HdrC,_Heterodisulfide_reductase,_subunit_C	4	0.8017	0.175
6	6	21.4	contig00004_-_Glucosamine-6-phosphate_isomerases/6-phosphogluconolactonase	4	0.4207	0.136
6	6	14.5	contig00002_-_tpiA,_triosephosphate_isomerase	3	0.787	0.2072
6	6	21.2	contig00030_-_nusG,_transcription_antitermination_protein_NusG	5	4.7424	0.0881
6	6	11	contig00013_-_type_IV_pilus_biogenesis/stability_protein_PilW	3	11.4815	0.0275
6	6	10.5	contig00007_-_1-(5-phosphoribosyl)-5-[(5-_phosphoribosylamino)methylideneamino]_imidazole-4-carboxamide_isomerase	3	0.4831	0.0316
6	6	10	contig00006_-_peptidase_PmbA	3	1.1912	0.5815
6	6	25.6	contig00005_-_YfaZ_family_protein	5	4.3251	0.0875
5.96	6	11.8	contig00013_-_Putative_methyl-accepting_chemotaxis_AlkN	3	1.7701	0.2692
5.89	6.02	25.6	contig00006_-_DsrE/DsrF-like_family	4	0.2168	0.1299
5.82	5.82	19.8	contig00005_-_RbsK,_Sugar_kinases,_ribokinase_family	3	0.5649	0.4171
5.78	5.78	4.5	contig00003_-_TonB-dependent_siderophore_receptor	3	1.1588	0.6452
5.77	5.8	43.9	contig00005_-_CopC_domain	6	1.1169	0.4053
5.73	5.73	22.3	contig00004_-_inorganic_pyrophosphatase	3	0.9908	0.7345
5.62	5.62	11.6	contig00005_-_argJ,_bifunctional_ornithine_acetyltransferase/N-acetylglutamate_synthase_protein	3	2.2491	0.2238
5.44	5.44	26.7	contig00006_-_arsenate_reductase	5	2.9648	0.3867

5.35	5.35	8.6	contig00003_-_phosphoenolpyruvate_synthase	3	1.0568	0.9662
5.35	5.35	9.1	contig00003_-_putative_quinone_oxidoreductase,_YhdH/YhfP_family	3	0.8954	0.9749
5.31	5.31	18.7	contig00016_-_Curved_DNA-binding_protein	3	1.888	0.3948
5.31	5.31	41.3	contig00009_-_molybdopterin_biosynthesis_protein_MoeB	5	1.1588	0.4628
5.1	5.1	39.9	contig00015_-_Dihydroneopterin_aldolase	3	2.0324	0.0022
4.92	4.92	7.7	contig00010_-_pyruvate_dehydrogenase	3	2.8054	0.1182
4.91	4.91	32.2	contig00016_-_IbpA,_Molecular_chaperone	4	0.1148	0.1601
4.9	4.93	29.9	contig00004_-_Putative_lipoprotein	3	1.1376	0.5471
4.82	4.82	14	contig00009_-_N-ethylmaleimide_reductase	3	0.3076	0.1531
4.8	4.8	42.7	contig00026_-_Putative_pterin-4_alpha-carbinolamine_dehydratase-like_protein	5	1.0375	0.706
4.78	4.78	52.8	contig00001_-_infA,_translation_initiation_factor_IF-1	3	1.3804	0.4493
4.76	4.76	19.7	contig00009_-_gabD1,_succinic_semialdehyde_dehydrogenase	3	0.2168	0.1433
4.75	4.75	16.1	contig00014_-_DdpA,_ABC-type_dipeptide_transport_system,_periplasmic_component	3	4.8306	0.0314
4.74	4.74	11.6	contig00002_-_Translation_initiation_factor_IF-2	3	1.3183	0.4626
4.63	4.63	12.6	contig00003_-_PKD_domain_containing_protein	3	2.3335	0.0802
4.6	4.6	51.7	contig00010_-_hypothetical_protein	11	3.6308	0.2713
4.57	4.57	24.9	contig00007_-_Pilus_assembly_protein,_PilP	3	5.6494	0.2619
4.53	4.63	12.6	contig00006_-_Lpd,_Pyruvate/2-oxoglutarate_dehydrogenase_complex,_dihydrolipoamide_dehydrogenase_(E3)_component,_and_related_enzymes	2	0.955	0.9282
4.47	4.47	15.9	contig00011_-_L,D-transpeptidase_catalytic_domain	4	7.2444	0.0382
4.43	4.43	25.2	contig00002_-_Protein_GrpE	4	2.9648	0.3765
4.37	5.07	25.2	contig00011_-_leuD,_isopropylmalate_isomerase_small_subunit	3	0.7798	0.0947
4.37	4.37	24.5	contig00010_-_nusB,_transcription_antitermination_protein_NusB	2	1.2706	0.328
4.36	4.37	23.1	contig00008_-_Uncharacterized_isochorismatase_family_protein_YwoC	3	0.1738	0.0566
4.32	4.33	16.3	contig00005_-_ATP-dependent_chaperone_ClpB	2	0.1614	0.2816
4.3	4.3	22.5	contig00001_-_lolA,_lipoprotein_chaperone	3	5.445	0.1055
4.28	4.28	50.6	contig00003_-_rpmA,_50S_ribosomal_protein_L27	3	0.9376	0.1983
4.22	4.22	11.5	contig00002_-_pyridoxine_5'-phosphate_synthase	2	0.8472	0.4347
4.18	4.18	11.5	contig00016_-_ATP-dependent_protease_subunit_HslV	3	3.4674	0.2176
4.17	4.17	21.9	contig00002_-_oligoribonuclease	3	2.884	0.0849
4.17	4.17	17.9	contig00002_-_Tetratricopeptide_TPR_2_repeat_protein	2	2.0701	0.4485
4.15	4.15	23	contig00026_-_Bacterioferritin_protein	3	0.3873	0.5503
4.14	4.15	22.8	contig00002_-_Thioredoxin_domain-containing_protein	2	2.2699	0.106
4.12	4.14	24.8	contig00006_-_This_family_is_most_closely_related_to_the_GT1_family_of_glycosyltransferases	3	2.884	0.2185
4.12	4.12	28.6	contig00001_-_putative_nucleotide-binding_protein	3	2.355	0.2082
4.12	4.12	11.6	contig00001_-_TolC,_Outer_membrane_protein	2	0.7656	0.515
4.11	4.11	23.7	contig00007_-_Yqey-like_protein	5	2.2284	0.3252
4.08	4.08	19.8	contig00002_-_Cupin_domain	2	0.1556	0.2545
4.06	4.06	14.3	contig00021_-_inositol_monophosphatase	3	2.1478	0.3907

4.06	4.06	13.7	contig00003_-_Sporulation_related_domain	3	0.3133	0.3123
4.05	4.05	18	contig00003_-_F0F1_ATP_synthase_subunit_delta	2	3.9446	0.267
4.05	4.05	18.2	contig00008_-_ubiquinol-cytochrome_c_reductase,_iron-sulfur_subunit	2	2.5586	0.0778
4.05	4.05	13	contig00002_-_Cytidylate_kinase-like_family	2	1.7219	0.4678
4.03	4.03	17.2	contig00016_-_rph,_ribonuclease_PH	2	1.7701	0.353
4.03	4.03	13.8	contig00005_-_Cation_efflux_system_protein_CzcB	2	0.1057	0.0264
4.02	4.02	9.2	contig00002_-_DNA_gyrase_subunit_A	2	0.5012	0.3235
4.02	4.02	29.4	contig00014_-_short_chain_dehydrogenase	3	1.6904	0.0642
4.02	4.02	14.7	contig00035_-_TolQ,_Biopolymer_transport_proteins	2	1.7378	0.1225
4.01	4.03	12.2	contig00001_-_fabG,_3-ketoacyl-(acyl-carrier-protein)_reductase	2	0.7943	0.3255
4.01	4.01	20.9	contig00009_-_Membrane_Fusion_Protein_cluster_2	3	0.0328	0.0659
4.01	4.01	7.4	contig00001_-_periplasmic_serine_protease,_Do/DeqQ_family	2	0.1486	0.2378
4.01	4.01	8.4	contig00007_-_Phosphomannomutase/phosphoglucomutase	2	0.5861	0.4606
4.01	4.01	14.4	contig00006_-_rbcR,_LysR_transcriptional_regulator	2	0.7447	0.6496
4.01	4.01	23.9	contig00006_-_50S_ribosomal_protein_L18	3	1.4588	0.6116
4.01	4.01	20.3	contig00011_-_Putative_signal_peptide_protein	4	0.1977	0.166
4.01	4.01	14.5	contig00010_-_Nucleoprotein/polynucleotide-associated_enzyme	2	0.5702	0.2557
4.01	4.01	24.4	contig00004_-_Penicillin-binding_protein_5	2	0.4446	0.316
4	6	23	contig00016_-_Putative_phosphoribosylformimino-5-aminoimidazole_carboxamide_ribotide_isomerase	3	2.9648	0.2299
4	4.29	36.9	contig00026_-_rpsI,_30S_ribosomal_protein_S9	3	1.0965	0.8527
4	4.04	8.2	contig00015_-_BisC,_Anaerobic_dehydrogenases,_typically_selenocysteine-containing	2	1.4322	0.2058
4	4.02	12.4	contig00009_-_Prc,_Periplasmic_protease	2	0.0637	0.1264
4	4.01	27.5	contig00024_-_3-hydroxydecanoyl-(acyl_carrier_protein)_dehydratase	2	1.4191	0.6027
4	4	27.7	contig00007_-_Pilus_assembly_protein,_PilO	2	0.0832	0.1017
4	4	27.6	contig00026_-_2-nonaprenyl-3-methyl-6-methoxy-1,4-benzoquinol_hydroxylase	2	7.2444	0.3373
4	4	15.7	contig00025_-_DsrE/DsrF/DrsH-like_family	2	0.2884	0.0975
4	4	8.3	contig00020_-_Outer_membrane_beta_barrel_protein	2	2.1086	0.2232
4	4	6.4	contig00003_-_ileS,_isoleucyl-tRNA_synthetase	2	0.9376	0.5134
4	4	37.2	contig00030_-_Elongation_factor_G_C-terminus	3	1.0568	0.9506
4	4	24.7	contig00026_-_rplM,_50S_ribosomal_protein_L13	3	1.0186	0.9556
4	4	21.7	contig00015_-_hypothetical_protein	2	2.5823	0.322
4	4	26	contig00011_-_Predicted_peroxiredoxins	10	1.7539	0.3621
4	4	11.9	contig00009_-_glycine_dehydrogenase_subunit_2	2	0.2443	0.1249
4	4	8.6	contig00009_-_glycine_dehydrogenase_subunit_1	4	1.0471	0.7298
4	4	11.3	contig00005_-_succinyl-CoA_synthetase_subunit_alpha	3	0.6026	0.4466
4	4	6.1	contig00002_-_mltD,_membrane-bound_lytic_murein_transglycosylase_D	3	0.2535	0.1779
4	4	29	contig00002_-_Universal_stress_protein_MJ0531	2	0.6194	0.6752
4	4	17.3	contig00002_-_DsrE/DsrF-like_family	3	3.5975	0.1818
4	4	6.9	contig00020_-_Succinate-semialdehyde_dehydrogenase_[NADP(+)]_GabD	3	2.9923	0.2049

4	4	23.7	contig00014_-_Uncharacterized_BCR,_YaiI/YqxD_family_COG1671	2	3.6308	0.1867
4	4	30.2	contig00013_-_RNA_recognition_motif	2	1.5704	0.4497
4	4	8	contig00012_-_GTP-binding_protein_YchF	4	0.9036	0.4865
4	4	39.6	contig00007_-_Sec-independent_protein_translocase_protein_TatA	2	2.9923	0.3202
4	4	9.9	contig00007_-_Orotate_phosphoribosyltransferase	3	1.2359	0.4627
4	4	33.7	contig00006_-_rpsP,_30S_ribosomal_protein_S16	2	0.0692	0.3567
4	4	24.8	contig00006_-_iojap-like_ribosome-associated_protein	2	1.7219	0.2798
4	4	4.5	contig00006_-_glpC,_sn-glycerol-3-phosphate_dehydrogenase_subunit_C	2	1.3428	0.5751
4	4	30.1	contig00006_-_Putative_lipoprotein	2	1.8197	0.3252
4	4	15.1	contig00006_-_Proline-rich_region	2	0.1213	0.2337
4	4	43.2	contig00006_-_50S_ribosomal_protein_L15	2	1.1169	0.8103
4	4	19	contig00005_-_UspA_domain_protein	3	0.9908	0.9572
4	4	11.1	contig00005_-_Putative_NADH_dehydrogenase/NAD(P)H_nitroreductase_AF_0226	2	0.6855	0.4753
4	4	12	contig00004_-_OmpR,_Response_regulators_consisting_of_a_CheY-like_receiver_domain_and_a_winged-helix_DNA-binding_domain	2	0.0565	0.1414
4	4	7.7	contig00003_-_glyQ,_glycyl-tRNA_synthetase_subunit_alpha	2	0.1096	0.2485
4	4	11.6	contig00003_-_PF03625_domain_protein	2		
4	4	24.2	contig00002_-_ihfB,_integration_host_factor_subunit_beta	2	1.0186	0.7729
4	4	2.8	contig00026_-_anthranilate_synthase_component_I	2	0.0366	0.1861
4	4	25.8	contig00022_-_carboxysome_peptide_A	5	1.4723	0.4259
4	4	19.1	contig00017_-_minE,_cell_division_topological_specificity_factor_MinE	2	1.0471	0.7766
4	4	17.9	contig00016_-_rpmE,_50S_ribosomal_protein_L31	3	2.3768	0.3227
4	4	6.2	contig00016_-_periplasmic_serine_protease,_Do/DeqQ_family	2	3.2211	0.2944
4	4	18.4	contig00015_-_acetyl-CoA_carboxylase_biotin_carboxyl_carrier_protein_subunit	4		
4	4	6.1	contig00014_-_HDOD_domain	2	0.0445	0.1738
4	4	3.8	contig00013_-_hscA,_chaperone_protein_HscA	3		
4	4	16.6	contig00012_-_EVE_domain	2	0.8872	0.8065
4	4	1.9	contig00008_-_cytochrome_c_oxidase,_subunit_I	2	1.0765	0.941
4	4	9.9	contig00008_-_Hemerythrin_HHE_cation_binding_domain	2	0.1995	0.3376
4	4	5.4	contig00005_-_glutamate-1-semialdehyde_aminotransferase	2	0.2032	0.1204
4	4	16.9	contig00005_-_PTS_IIA-like_nitrogen-regulatory_protein_PtsN	2	1.9588	0.4088
4	4	19	contig00005_-_ManX,_Phosphotransferase_system,_mannose/fructose-specific_component_IIA	2	0.6427	0.4217
4	4	5.4	contig00003_-_hemC,_porphobilinogen_deaminase	2	1.0666	0.7906
4	4	18.3	contig00001_-_PII-like_signaling_protein	2	3.4356	0.2217
3.92	4	7.2	contig00003_-_dihydroorotate_dehydrogenase_1B	2	0.4966	0.1903
3.92	4	34.4	contig00001_-_Trm112p-like_protein	2	0.4786	0.5236
3.89	3.89	11.7	contig00006_-_putative_GTP_cyclohydrolase	2	0.955	0.4819
3.85	3.85	21.2	contig00005_-_Cell_division_protein_ZapD	2	0.263	0.1839
3.82	3.82	5.6	contig00003_-_ATP_synthase_epsilon_chain	2	10.7647	0.2683

3.81	3.81	15.8	contig00006_-_tldD,_protease_TldD	3	0.6982	0.7371
3.8	3.8	14.8	contig00002_-_Pterin_4_alpha_carbinolamine_dehydratase	2	0.7798	0.695
3.78	3.78	40.7	contig00010_-_Putative_cytoplasmic_protein	2	1.1695	0.7387
3.75	3.75	14.3	contig00003_-_Glutaredoxin,_GrxC_family	2	0.5495	0.5196
3.66	3.66	9	contig00004_-_SuhB,_Archaeal_fructose-1,6-bisphosphatase_and_related_enzymes_of_inositol_monophosphatase_family	2	2.7542	0.3649
3.64	3.76	14.7	contig00017_-_dihydrolipoamide_dehydrogenase	2	0.8472	0.4897
3.62	3.63	14.8	contig00007_-_RNA_polymerase_sigma_factor_RpoD	2	0.9204	0.5404
3.62	3.62	16.2	contig00026_-_Cytochrome_c553	2	5.445	0.2301
3.59	3.59	25.9	contig00004_-_Phospholipid-binding_protein	2	0.6252	0.5678
3.54	3.58	3.9	contig00007_-_phosphoglyceromutase	2	0.6194	0.3698
3.48	3.48	5	contig00022_-_CheA,_Chemotaxis_protein_histidine_kinase_and_related_kinases	2	0.012	0.0723
3.47	3.56	12.7	contig00016_-_VacJ,_Surface_lipoprotein	2	1.028	0.8787
3.41	3.41	14.8	contig00013_-_acetyl-CoA_carboxylase_carboxyltransferase_subunit_alpha	2	0.8017	0.9108
3.39	3.39	46.9	contig00008_-_Membrane_fusogenic_activity	5	0.5152	0.3496
3.29	5.56	14.9	contig00001_-_clpA,_ATP-dependent_Clp_protease_ATP-binding_subunit	3	1.028	0.8213
3.28	3.28	14.4	contig00016_-_dapF,_diaminopimelate_epimerase	2	1.2246	0.5795
3.26	3.26	8.4	contig00001_-_Phosphoketolase	3	0.0425	0.1464
3.2	3.2	47.3	contig00015_-_50S_ribosomal_protein_L33	3	1.556	0.7749
3.14	3.14	8.9	contig00002_-_Lon,_ATP-dependent_Lon_protease,_bacterial_type	3	0.9638	0.8535
3.11	3.11	6.5	contig00008_-_NirB,_NAD(P)H-nitrite_reductase	3		
3.1	3.1	16.3	contig00001_-_PepB,_Leucyl_aminopeptidase	2	0.2051	0.1962
3.05	3.09	9.4	contig00003_-_glyS,_glycyl-tRNA_synthetase_subunit_beta	2	2.1478	0.2921
3.05	3.05	37.9	contig00004_-_glycine_cleavage_system_protein_H	5	2.466	0.4026
3.03	3.03	21.1	contig00006_-_rplS,_50S_ribosomal_protein_L19	3	0.169	0.1882
3	3	9.3	contig00002_-_Segregation_and_condensation_protein_B_homolog	2	1.2359	0.177
2.98	2.98	14.8	contig00007_-_DNA_topoisomerase_1	2	1.3552	0.2763
2.88	2.88	21.5	contig00002_-_peptidyl-prolyl_cis-trans_isomerase_B	4	2.3988	0.2888
2.83	2.83	13.6	contig00007_-_pyrroline-5-carboxylate_reductase	2	0.9462	0.8207
2.79	2.79	50.8	contig00006_-_rpmD,_50S_ribosomal_protein_L30	3	1.1272	0.5974
2.76	2.81	12.5	contig00008_-_Tas,_Predicted_oxidoreductases	2	0.3373	0.2221
2.74	2.74	8.8	contig00007_-_DNA_topoisomerase_I	2	1.8707	0.7037
2.62	2.62	16.5	contig00012_-_ribose-5-phosphate_isomerase_A	2	0.169	0.202
2.55	2.55	15.2	contig00010_-_bifunctional_3,4-dihydroxy-2-butanone_4-phosphate_synthase/GTP_cyclohydrolase_II-like_protein	2	0.15	0.1234
2.51	2.51	19.4	contig00004_-_Sulfur_oxidation_protein_SoxY	2	3.9446	0.2766
2.48	2.48	16.5	contig00004_-_Protein_TolB	2	2.466	0.3356
2.47	2.47	9.6	contig00007_-_Hpt_domain	2	1.1169	0.6297
2.44	2.45	4.8	contig00005_-_Cellulose_synthase_operon_protein_C_C-terminus	2	0.3733	0.2056
2.43	2.43	34.3	contig00001_-_Lipoprotein	2	6.8549	0.2312

2.38	2.38	19.2	contig00001_-_nudF,_ADP-ribose_pyrophosphatase_NudF	2	11.272	0.0322
2.38	2.38	13.6	contig00015_-_Domain_of_unknown_function_(DUF4340)	1	0.0384	0.1019
2.36	2.37	5.6	contig00002_-_clpX,_ATP-dependent_protease_ATP-binding_subunit_ClpX	1	0.7311	0.5781
2.36	2.36	6.3	contig00022_-_S-adenosylmethionine_synthetase	1	0.6982	0.3642
2.33	2.33	8.5	contig00025_-_Cobalamin_adenosyltransferase	2	7.0469	0.1257
2.32	2.33	21.9	contig00008_-_Uncharacterized_ACR,_YkgG_family_COG1556	1	1.3428	0.5764
2.32	2.32	45.1	contig00006_-_rplN,_50S_ribosomal_protein_L14	2	1.7539	0.2249
2.32	2.32	5.9	contig00012_-_TPR_repeat-containing_protein_PA4667	1	0.4487	0.7858
2.31	2.32	17.9	contig00001_-_hypoxanthine-guanine_phosphoribosyltransferase	1	0.8551	0.9216
2.3	2.3	20.5	contig00014_-_Flagellar_basal_body-associated_protein_FliL	1	10.5682	0.1687
2.29	2.29	16.2	contig00008_-_SCO1_protein_homolog	2	0.6368	0.4529
2.28	2.28	19.8	contig00003_-_30S_ribosomal_protein_S20	2	1.4191	0.5279
2.26	4.2	9.3	contig00012_-_efflux_transporter,_outer_membrane_factor_(OMF)_lipoprotein,_NodT_family	4	1.977	0.3295
2.26	2.26	14.4	contig00022_-_ATP-dependent_dethiobiotin_synthetase_BioD	2	0.871	0.9023
2.25	2.26	17.7	contig00021_-_hypothetical_protein	1	1.0186	0.7346
2.24	2.24	19.2	contig00004_-_Cytochrome_C_oxidase,_cbb3-type,_subunit_III	2	1.4588	0.6691
2.23	2.23	27	contig00002_-_Cupredoxin-like_domain	1	3.1333	0.249
2.2	2.2	8.2	contig00004_-_aspartate_kinase	2	1.0471	0.7961
2.16	2.16	26.8	contig00014_-_Tetratricopeptide_TPR_1_repeat-containing_protein	1	1.0186	0.1106
2.14	2.15	5.5	contig00006_-_AcrB,_Cation/multidrug_efflux_pump	1	0.6081	0.4707
2.14	2.14	17.7	contig00026_-_Indole-3-glycerol_phosphate_synthase	1	0.169	0.3522
2.12	2.12	11.2	contig00004_-_antiporter_inner_membrane_protein	1	1.0666	0.8721
2.11	2.11	9.2	contig00020_-_Ferredoxin--NADP_reductase	1	1.0568	0.779
2.1	2.1	11.6	contig00012_-_prfA,_peptide_chain_release_factor_1	1	0.6194	0.3628
2.08	2.08	17.1	contig00016_-_AhpC/TSA_family	2	11.1686	0.0018
2.08	2.08	7.4	contig00005_-_UDP-N-acetylmuramoyl-tripeptide--D-alanyl-D-_alanine_ligase	2	0.4656	0.234
2.07	2.08	7.5	contig00012_-_Asparaginase	2	1.6444	0.4164
2.06	2.06	7	contig00015_-_dipZ,_thiol:disulfide_interchange_protein_precursor	1	1.9588	0.3358
2.06	2.06	5.5	contig00001_-_NMT1-like_family	1	0.5105	0.3398
2.05	2.05	12.5	contig00010_-_recombination_and_DNA_strand_exchange_inhibitor_protein	1	1.7865	0.1784
2.05	2.05	13.2	contig00006_-_Uncharacterized_conserved_secreted_or_membrane_protein	2	1.2474	0.8153
2.05	2.05	4.7	contig00005_-_aspS,_aspartyl-tRNA_synthetase	1	0.8318	0.2532
2.04	2.04	11.3	contig00017_-_ABC_transporter,_phosphonate,_periplasmic_substrate-binding_protein	2	1.2589	0.4888
2.04	2.04	3.7	contig00001_-_phosphoribosylformylglycinamidine_synthase	1	0.1445	0.2672
2.04	2.04	7.2	contig00001_-_fructose-bisphosphate_aldolase	2	0.0115	0.1395
2.04	2.04	27.4	contig00001_-_50S_ribosomal_protein_L32	1	0.5152	0.3469
2.03	2.03	13.6	contig00001_-_General_stress_protein_69	1	0.7798	0.4583
2.03	2.03	4.8	contig00006_-_purT,_phosphoribosylglycinamide_formyltransferase_2	1	0.7727	0.4661
2.03	2.03	12.2	contig00017_-_PleD,_Response_regulator_containing_a_CheY-like_receiver_domain_and_a_GGDEF_domain	2	0.6855	0.5612

2.02	2.03	39.7	contig00006_-_50S_ribosomal_protein_L17	1	1.0666	0.8868
2.02	2.02	13.9	contig00006_-_signal_recognition_particle_protein	1	2.1478	0.2963
2.02	2.02	8.4	contig00018_-_Protein_RcaC	1	0.4406	0.2463
2.02	2.02	5	contig00016_-_dihydrolipoamide_dehydrogenase	1	3.5318	0.19
2.02	2.02	4.9	contig00010_-_glutamate_decarboxylase	1	0.4055	0.2626
2.02	2.02	2.8	contig00003_-_c-di-GMP_phosphodiesterase_class_II	1	0.6918	0.5291
2.02	2.02	14.9	contig00002_-_OlmA,_Outer_membrane_lipoprotein_OmlA	2	2.2491	0.2727
2.02	2.02	14.3	contig00001_-_ATP-dependent_Clp_protease_adapter_protein_ClpS	1	5.5463	0.0337
2.01	4.03	10.5	contig00010_-_Hemolysin_secretion_protein	3	0.8318	0.7302
2.01	2.03	11.3	contig00006_-_heat_shock_protein_90	1	0.15	0.531
2.01	2.02	4.7	contig00012_-_multidrug_efflux_protein	1	0.3908	0.267
2.01	2.01	9.9	contig00010_-_dihydroxy-acid_dehydratase	1	2.1281	0.3001
2.01	2.01	4.1	contig00006_-_leuS,_leucyl-tRNA_synthetase	1		
2.01	2.01	12	contig00026_-_Bacterial_microcompartments_are_primitive_organelles_composed_entirely_of_protein_subunits	2	0.6668	0.3482
2.01	2.01	11.4	contig00006_-_GuaA,_GMP_synthase_-_Glutamine_amidotransferase_domain	2	0.0417	0.1298
2.01	2.01	13.9	contig00022_-_N5-carboxyaminoimidazole_ribonucleotide_mutase	1	0.3436	0.2119
2.01	2.01	8.7	contig00013_-_hscB,_co-chaperone_HscB	1	1.4723	0.3554
2.01	2.01	13.7	contig00007_-_bifunctional_pyrimidine_regulatory_protein_PyrR_uracil_phosphoribosyltransferase	1	0.673	0.6141
2.01	2.01	7.3	contig00004_-_Dyp-type_peroxidase_family	1	1.2942	0.0339
2.01	2.01	9.6	contig00001_-_Predicted_transcriptional_regulator	1	1.2942	0.6224
2	17.88	41.7	contig00027_-_Gram-negative_porin	28	3.5975	0.2243
2	8	25	contig00005_-_Nitrogen_regulatory_protein_P-II	5	4.6132	0.2238
2	4.01	8	contig00009_-_Hemolysin_secretion_protein	2	5.1051	0.1493
2	4	4.8	contig00005_-_Transcriptional_regulator	2	0.8091	0.6906
2	3.59	3.9	contig00001_-_aspartate_aminotransferase	2	0.2535	0.1775
2	2.09	4.7	contig00011_-_methylmalonic_acid_semialdehyde_dehydrogenase	2		
2	2.03	5.3	contig00005_-_LppC_putative_lipoprotein	2	0.6668	0.4958
2	2.03	7	contig00002_-_Predicted_3'-5'_exonuclease_related_to_the_exonuclease_domain_of_PolB	1	1.2823	0.6251
2	2.02	8.5	contig00015_-_ATP-dependent_RNA_helicase_RhlB	2		
2	2.02	9.6	contig00008_-_Cytochrome_C1_family	1		
2	2.01	15.1	contig00011_-_Vi_polysaccharide_biosynthesis_protein_TviB	1	0.3664	0.2779
2	2.01	9.8	contig00006_-_prolyl-tRNA_synthetase	1	0.8091	0.1091
2	2.01	7.9	contig00011_-_tryptophan_synthase_subunit_beta	1	1.5996	0.3556
2	2.01	14.3	contig00007_-_Competence_protein_A	1	1.1272	0.9082
2	2.01	6.8	contig00020_-_Curved_DNA-binding_protein	1	0.8017	0.7275
2	2.01	4.3	contig00007_-_Voltage_gated_chloride_channel	1	0.7586	0.8729
2	2.01	5.7	contig00011_-_Protein_CapI	1	0.0112	0.0579
2	2.01	8.4	contig00009_-_histidinol-phosphatase,_inositol_monophosphatase_family	1	0.0631	0.2863
2	2.01	12.8	contig00003_-_helix_turn_helix,_Arsenical_Resistance_Operon_Repressor	1	1.0471	0.833

2	2.01	10.8	contig00002_-_Carbamoyl-phosphate_synthase_small_chain,_CPSase_domain	1	0.7798	0.8329
2	2.01	13.3	contig00001_-_ihfA,_integration_host_factor_subunit_alpha	1	1.2246	0.5777
2	2	7.2	contig00004_-_metG,_methionyl-tRNA_synthetase	1	0.2188	0.1613
2	2	8.7	contig00024_-_thiamine_biosynthesis_protein_ThiC	1	0.6546	0.4848
2	2	12.8	contig00005_-_sucC,_succinyl-CoA_synthetase_subunit_beta	1	0.4285	0.3515
2	2	14.5	contig00005_-_Bacterial_cellulose_synthase_subunit	1	0.9638	0.9558
2	2	32.8	contig00005_-_3-deoxy-D-manno-octulosonate_8-phosphate_phosphatase_KdsC	1	0.0111	0.0639
2	2	10.1	contig00003_-_membrane_protein_insertase	1	0.1905	0.1481
2	2	18.2	contig00002_-_RsuA,_16S_rRNA_uridine-516_pseudouridylate_synthase_and_related_pseudouridylate_synthases	1	1.1169	0.8113
2	2	11.3	contig00026_-_Trypsin-like_peptidase_domain	1	1.0568	0.992
2	2	6.4	contig00026_-_Putative_transmembrane_protein_coupled_to_NADH-ubiquinone_oxidoreductase	1	0.8241	0.7242
2	2	9.9	contig00022_-_S-adenosyl-L-homocysteine_hydrolase	1	0.1528	0.3147
2	2	18.5	contig00003_-_ispH,_4-hydroxy-3-methylbut-2-enyl_diphosphate_reductase	1	1.0965	0.8996
2	2	11.6	contig00002_-_SseA,_Rhodanese-related_sulfurtransferase	1	0.1837	0.2728
2	2	24.8	contig00002_-_Dienelactone_hydrolase_family	1	1.4588	0.9086
2	2	8.5	contig00015_-_moaA,_molybdenum_cofactor_biosynthesis_protein_A	1	2.0324	0.2863
2	2	11.4	contig00013_-_Histidine--tRNA_ligase	1	0.2704	0.2828
2	2	17.7	contig00011_-_Ttg2C,_ABC-type_transport_system_involved_in_resistance_to_organic_solvents	1	0.5058	0.3355
2	2	9.4	contig00010_-_Probable_ATP-dependent_RNA_helicase_ddx27	2	3.3729	0.3537
2	2	13.1	contig00007_-_glutamate_synthase_small_subunit_family_protein,_proteobacterial	1	0.673	0.5083
2	2	9.5	contig00007_-_Protein_PilJ	1	1.5704	0.6052
2	2	8.8	contig00006_-_phosphoribosylaminoimidazole_synthetase	1	0.7798	0.6476
2	2	13.6	contig00004_-_Glucokinase	1		
2	2	10.9	contig00003_-_Sulfide:quinone_oxidoreductase,_mitochondrial	1	0.2249	0.1647
2	2	8.6	contig00001_-_isocitrate_dehydrogenase	1	0.278	0.1899
2	2	11.1	contig00001_-_Cytochrome_c4	1	2.0701	0.3123
2	2	4.8	contig00027_-_TonB-dependent_siderophore_receptor	1		
2	2	28.1	contig00024_-_Predicted_transcriptional_regulator	1	2.8576	0.226
2	2	6.5	contig00022_-_Carboxysome_Shell_Carbonic_Anhydrase	1	0.6855	0.9774
2	2	13.6	contig00017_-_monothiol_glutaredoxin,_Grx4_family	1		
2	2	18.4	contig00016_-_PAS_sensor_protein	1	0.6026	0.4019
2	2	6.9	contig00015_-_bifunctional_phosphoribosylaminoimidazolecarboxamide_formyltransferase/IMP_cyclohydrolase	1	0.6252	0.449
2	2	25.3	contig00013_-_Chaperone_protein_HscA_homolog	1	1.7865	0.3647
2	2	7.8	contig00012_-_GIY-YIG_catalytic_domain	1	0.0787	0.2523
2	2	12.2	contig00011_-_trpA,_tryptophan_synthase_subunit_alpha	1	0.787	0.6587
2	2	6.1	contig00010_-_phosphoenolpyruvate_carboxylase	1	0.0586	0.1421
2	2	9.8	contig00010_-_pgi,_glucose-6-phosphate_isomerase	1	0.1941	0.237
2	2	6.2	contig00009_-_tyrosyl-tRNA_synthetase	1	1.7539	0.4555
2	2	8.5	contig00009_-_ZnuA,_ABC-type_Zn2+_transport_system,_periplasmic_component/surface_adhesin	2	8.6298	0.2196

2	2	19.8	contig00007_-_hisE,_phosphoribosyl-ATP_pyrophosphatase	1	0.4529	0.2953
2	2	6.4	contig00005_-_Type_4_fimbrial_assembly_protein_PilC	1	0.263	0.1825
2	2	9.4	contig00004_-_helix_turn_helix_multiple_antibiotic_resistance_protein	1	11.3763	0.3157
2	2	13.3	contig00004_-_YdcF-like_protein	1	0.7656	0.6462
2	2	5.5	contig00004_-_LPS-assembly_protein_LptD	1	1.0093	0.3766
2	2	24.2	contig00002_-_Signal_peptidase_I	2	0.5297	0.3432
2	2	10.4	contig00002_-_Predicted_kinase	1	0.4207	0.2722
2	2	5.2	contig00002_-_N-ethylammeline_chlorohydrolase	1	1.1695	0.5378
2	2	11.7	contig00002_-_FHA_domain	4	1.6444	0.5073
2	2	16.7	contig00002_-_Escherichia_coli_YhbY_is_associated_with_pre-50S_ribosomal_subunits	1	3.6644	0.375
2	2	16.3	contig00001_-_lipoprotein_releasing_system,_ATP-binding_protein	1	0.7047	0.4222
2	2	6.5	contig00001_-_ferrochelatase	1	0.787	0.8523
2	2	24.3	contig00036_-_Cysteine_desulfhydrase_/_Selenocysteine_lyase	1	0.5395	0.3635
2	2	6.5	contig00022_-_RuBisCO_operon_transcriptional_regulator	1	0.52	0.3458
2	2	4.8	contig00020_-_periplasmic_serine_protease,_Do/DeqQ_family	1	0.7311	0.5772
2	2	12.3	contig00017_-_Thioredoxin_domain-containing_protein	1	0.6607	0.4912
2	2	10.4	contig00015_-_acetyl-CoA_carboxylase_biotin_carboxylase_subunit	1	1.7865	0.3744
2	2	12.2	contig00015_-_WcaG,_Nucleoside-diphosphate-sugar_epimerases	1	0.8551	0.7719
2	2	6.9	contig00012_-_GntR,_Transcriptional_regulators	1	1.0568	0.8981
2	2	19	contig00012_-_Aspartyl/glutamyl-tRNA(Asn/Gln)_amidotransferase_subunit_C	1	0.7447	0.5908
2	2	7.6	contig00011_-_chorismate_synthase	1	1.2134	0.736
2	2	3.8	contig00011_-_amidophosphoribosyltransferase	1		
2	2	17.7	contig00010_-_ribH,_6,7-dimethyl-8-ribityllumazine_synthase	1	0.0249	0.1211
2	2	11.3	contig00010_-_Oxidoreductase,_aldo/keto_reductase_family	1	1.1803	0.7771
2	2	4.1	contig00010_-_Outer_membrane_protein_transport_protein	1	2.7542	0.2331
2	2	6.7	contig00010_-_NADP-dependent_L-serine/L-allo-threonine_dehydrogenase_YdfG	1	0.4446	0.2887
2	2	8.3	contig00009_-_glycine_cleavage_system_protein_H	2	0.9638	0.9173
2	2	11.7	contig00009_-_Vitamin_B12_dependent_methionine_synthase,_activation_domain	1	0.5297	0.3555
2	2	9.8	contig00009_-_Predicted_proteasome-type_protease	2	0.1294	0.3469
2	2	13.8	contig00009_-_Phosphoesterase_family	1	0.4656	0.6264
2	2	2.2	contig00008_-_Periplasmic_binding_protein	1	0.3945	0.297
2	2	7.9	contig00007_-_tRNA_threonylcarbamoyladenosine_biosynthesis_protein_RimN	1	0.871	0.7934
2	2	11.3	contig00007_-_ATPase_components_of_ABC_transporters_with_duplicated_ATPase_domains	1	0.3342	0.2204
2	2	28.2	contig00007_-_30S_ribosomal_protein_S21	1	0.7798	0.6595
2	2	11.4	contig00007_-_3'(2'),5'-bisphosphate_nucleotidase,_bacterial	1	0.6668	0.4909
2	2	6.3	contig00006_-_PhoH,_Phosphate_starvation-inducible_protein_PhoH,_predicted_ATPase	1	0.3565	0.2712
2	2	10.2	contig00006_-_Glycine-zipper_containing_OmpA-like_membrane_domain	2	0.4406	0.4984
2	2	4.4	contig00006_-_Flagellin_protein	1	33.1131	0.1016
2	2	6.4	contig00006_-_Chaperone_protein_HtpG	1	0.3221	0.213

2	2	5.4	contig00005_-_ddl,_D-alanine--D-alanine_ligase	1		
2	2	4.5	contig00005_-_UDP-N-acetylglucosamine_1-carboxyvinyltransferase	1		
2	2	10.4	contig00005_-_TolQ_protein	1	0.0331	0.1221
2	2	5.1	contig00005_-_Cell_division_protein_FtsA	1	1.556	0.4574
2	2	23	contig00004_-_TolA_protein	2	4.5709	0.2742
2	2	19.3	contig00004_-_PspE,_Rhodanese-related_sulfurtransferase	1	1.5704	0.4562
2	2	5.8	contig00004_-_Enolase-phosphatase_E1	1	0.9036	0.8582
2	2	3.4	contig00003_-_argininosuccinate_lyase	1	0.8551	0.7057
2	2	7.6	contig00003_-_Bacterial_protein_of_unknown_function_(DUF945)	1	1.3183	0.5955
2	2	4.7	contig00003_-_6-phosphofructokinase	1	0.5916	0.4112
2	2	5.7	contig00002_-_clpP,_ATP-dependent_Clp_protease_proteolytic_subunit	1	0.1236	0.3903
2	2	5.3	contig00002_-_Uncharacterized_oxidoreductase_YfjR	1	0.0692	0.1922
2	2	10.2	contig00002_-_FabI,_Enoyl-[acyl-carrier-protein]	1	0.1995	0.1526
2	2	6.1	contig00002_-_3-phosphoshikimate_1-carboxyvinyltransferase	1	1.5136	0.4782
2	2	9	contig00001_-_tmk,_thymidylate_kinase	1	0.1086	0.1118
2	2	12	contig00001_-_pyrH,_uridylate_kinase	1	0.5395	0.363
2	2	4.6	contig00001_-_3-oxoacyl-(acyl_carrier_protein)_synthase_III	1	0.5808	0.4048
2	2	9.4	contig00027_-_Thioredoxin-like_protein	2	5.9704	0.1584
2	2	3.4	contig00027_-_Tas,_Predicted_oxidoreductases	1	1.4454	0.5085
2	2	11.1	contig00025_-_AmpD_protein	1	1.2474	0.5091
2	2	2.9	contig00024_-_rfaD,_ADP-L-glycero-D-mannoheptose-6-epimerase	1		
2	2	3.5	contig00022_-_Probable_transcriptional_regulator_LumQ	1	2.884	0.2243
2	2	2.5	contig00018_-_Outer_membrane_protein_beta-barrel_domain	1	4.4463	0.317
2	2	5.2	contig00017_-_Chemotaxis_protein_CheY	1	0.4055	0.2678
2	2	5.4	contig00016_-_gmk,_guanylate_kinase	1	3.8019	0.216
2	2	1.6	contig00016_-_dihydrolipoamide_succinyltransferase	1	1.1803	0.727
2	2	3.6	contig00016_-_Sterol-binding_domain_protein	1	3.1915	0.3437
2	2	21.4	contig00015_-_CopZ,_Copper_chaperone	1	0.5702	0.9698
2	2	3.6	contig00014_-_Hydrogen_peroxide-inducible_genes_activator	1	1.5849	0.4062
2	2	5.5	contig00013_-_RNA_polymerase_sigma-70_factor,_TIGR02943_family	1	0.2858	0.1945
2	2	5	contig00012_-_upp,_uracil_phosphoribosyltransferase	1		
2	2	4.9	contig00012_-_Uncharacterized_Fe-S_protein	1	0.4831	0.3181
2	2	5.2	contig00012_-_SlpA,_FKBP-type_peptidyl-prolyl_cis-trans_isomerases_2	3	2.9648	0.29
2	2	2	contig00011_-_beta_alanine--pyruvate_transaminase	1		
2	2	4.4	contig00011_-_Peptidase_C26	1	0.5861	0.4066
2	2	7.2	contig00011_-_ABC-type_uncharacterized_transport_system,_auxiliary_component	1	3.5318	0.1906
2	2	5.3	contig00010_-_heavy_metal_response_regulator	1	2.1478	0.2981
2	2	14	contig00010_-_YciI-like_protein	1		
2	2	11.8	contig00009_-_iron-sulfur_cluster_insertion_protein_ErpA	2	2.5351	0.2516

2	2	8.1	contig00009_-_Rhodanese_Homology_Domain	1	1.2359	0.4743
2	2	5.3	contig00009_-_NAD(P)H:quinone_oxidoreductase	1	1.0471	0.9261
2	2	6.1	contig00008_-_Transcriptional_regulator_containing_an_AraC-type_DNA-binding_HTH_domain	1	0.3664	0.2386
2	2	10.2	contig00007_-_Hemerythrin_HHE_cation_binding_domain	2	0.9908	0.9876
2	2	6.3	contig00007_-_Cyclic_nucleotide-binding_domain	1	0.6982	0.5096
2	2	3.5	contig00006_-_secY,_preprotein_translocase_subunit_SecY	1	0.055	0.1487
2	2	4.6	contig00006_-_LPS-assembly_lipoprotein_LptE	1	2.4434	0.2615
2	2	7.9	contig00005_-_Sigma_54_modulation_protein_/_S30EA_ribosomal_protein	1	3.3729	0.2389
2	2	4	contig00005_-_Probable_3-mercaptopyruvate_sulfurtransferase	1	0.4487	0.2925
2	2	7.2	contig00005_-_Predicted_peroxiredoxins	1	0.7379	0.618
2	2	7.6	contig00004_-_fabG,_3-ketoacyl-(acyl-carrier-protein)_reductase	1	0.4406	0.2871
2	2	2.4	contig00004_-_Tetratricopeptide_TPR_4	1	1	0.9834
2	2	8.6	contig00004_-_Lipoate_regulatory_protein_YbeD	1	3.5318	0.1907
2	2	7.5	contig00004_-_Ferredoxin_1	1	1.0568	0.9012
2	2	3	contig00002_-_cmk,_cytidylate_kinase	1	1.3305	0.5934
2	2	4.7	contig00002_-_Ureidoglycolate_lyase	1	0.5649	0.4911
2	2	9.1	contig00002_-_Protein_YciI	1	1.2359	0.6145
2	2	21.7	contig00002_-_Protein-export_membrane_protein_SecG	1	0.7047	0.7053
2	2	3.4	contig00002_-_NADPH_dehydrogenase_NamA	1	0.3767	0.487
2	2	9.7	contig00001_-_ubiquinol-cytochrome_c_reductase,_iron-sulfur_subunit	1	5.7544	0.1394
2	2	5.4	contig00001_-_ompR,_osmolarity_response_regulator	1		
2	2	4.9	contig00001_-_UDP-N-acetylglucosamine_acyltransferase	1	3.8019	0.1803
2	2	18	contig00001_-_Acyl_carrier_protein	1	1.2246	0.7755
2	2	4.7	contig00001_-_5'-methylthioadenosine_phosphorylase	1		
1.92	2	10.9	contig00007_-_Putative_cell_wall_biogenesis_protein	1	1.3804	0.5856
1.92	2	11.4	contig00002_-_Scavenger_mRNA_decapping_enzyme_C-term_binding	1	0.929	0.9003
1.92	2	4.4	contig00002_-_PTC1,_Serine/threonine_protein_phosphatase	1	0.9817	0.9782
1.92	2	4.7	contig00002_-_NADH-quinone_oxidoreductase_subunit_J	1	2.466	0.4132
1.82	2	14.3	contig00002_-_PilZ_domain	1	1.3428	0.5821
1.74	2	9.2	contig00012_-_gatA,_aspartyl/glutamyl-tRNA_amidotransferase_subunit_A	1	1.1066	0.4531
1.74	2	2	contig00011_-_adenylosuccinate_synthetase	1	0.0191	0.1594
1.68	1.68	13.5	contig00014_-_ilvH,_acetolactate_synthase_3_regulatory_subunit	1	1.1272	0.5885
1.68	1.68	6.3	contig00013_-_lysS,_lysyl-tRNA_synthetase	1	0.492	0.1985
1.6	1.6	9.7	contig00003_-_branched-chain_amino_acid_aminotransferase	1	1.3305	0.5881
1.57	1.57	8.3	contig00006_-_RfbB,_dTDP-D-glucose_4,6-dehydratase	1	1.6144	0.4287
1.57	1.57	17.7	contig00026_-_Ferredoxin,_2Fe-2S	1	1.3428	0.579
1.52	1.52	1.7	contig00024_-_bifunctional_heptose_7-phosphate_kinase/heptose_1-phosphate_adenyltransferase	1	0.2992	0.1946
1.48	1.48	6.8	contig00017_-_Carboxymuconolactone_decarboxylase	1	0.879	0.8143
1.41	1.41	12.9	contig00003_-_GTPase_obg	1	1.8197	0.3646

Unused	Total	% Cov	Accession #	Peptides (95%)	117:115	PVal 117:115
45.99	45.99	62.8	contig00006_-_ectoine/hydroxyectoine_ABC_transporter_solute-binding_protein	44	55.9758	0
16.81	16.81	37.4	contig00012_-_50S_ribosomal_protein_L25/general_stress_protein_Ctc	15	41.3047	0.0169
2	2	5.4	contig00016_-_gmk,_guanylate_kinase	1	25.1189	0.1339
8	8	32.3	contig00016_-_reactive_intermediate/imine_deaminase	6	24.6604	0.0051
6.68	6.68	31.9	contig00021_-_yajC,_preprotein_translocase_subunit_YajC	5	23.9883	0.1389
22.23	22.23	62.5	contig00015_-_10_kDa_chaperonin	23	22.4905	0.0001
6	6	11	contig00013_-_type_IV_pilus_biogenesis/stability_protein_PilW	3	16.293	0.0218
8.05	8.05	42.2	contig00007_-_Protein_PilH	5	15.5597	0.0147
3.41	3.41	14.8	contig00013_-_acetyl-CoA_carboxylase_carboxyltransferase_subunit_alpha	2	15.2757	0.21
3.66	3.66	9	contig00004_-_SuhB,_Archaeal_fructose-1,6-bisphosphatase_and_related_enzymes_of_inositol_monophosphatase_family	2	14.9969	0.2053
14.98	14.98	67.1	contig00016_-_SirA-like_protein	14	14.9969	0.0016
4.3	4.3	22.5	contig00001_-_lolA,_lipoprotein_chaperone	3	14.5881	0.3028
16	16	48	contig00030_-_50S_ribosomal_protein_L7/L12	13	14.5881	0.0019
2	2	4.4	contig00006_-_Flagellin_protein	1	14.3219	0.1189
10.13	10.13	24.9	contig00002_-_Universally_conserved_protein	8	13.9316	0.0009
12.47	12.47	31.9	contig00002_-_greA,_transcription_elongation_factor_GreA	8	12.2462	0.6589
5.73	5.73	22.3	contig00004_-_inorganic_pyrophosphatase	3	11.9124	0.1849
8	8	23.1	contig00003_-_ATP_synthase_subunit_b	5	11.0662	0.0193
11.86	11.86	28	contig00004_-_peptidoglycan-associated_lipoprotein	21	10.8643	0.1507
26.12	26.12	47.3	contig00001_-_2,3,4,5-tetrahydropyridine-2,6-dicarboxylate_N-succinyltransferase	16	10.7647	0
15.72	15.72	57.1	contig00001_-_RNA_polymerase-binding_transcription_factor_DksA	10	10.666	0.0015
34.54	34.58	61.5	contig00008_-_Rusticyanin_protein	52	10.5682	0.0001
10	10	20.1	contig00013_-_Cytoskeleton_protein_RodZ	5	10.3753	0.0064
12.23	12.25	39.9	contig00005_-_Toluene_tolerance,_Ttg2	9	9.8175	0.0001
97.08	97.08	60	contig00015_-_60_kDa_chaperonin	107	9.8175	0
2.88	2.88	21.5	contig00002_-_peptidyl-prolyl_cis-trans_isomerase_B	4	9.7275	0.1585
4.6	4.6	51.7	contig00010_-_hypothetical_protein	11	9.1201	0.172
12.83	12.83	48.4	contig00002_-_bacterial_(prokaryotic)_histone_like_domain	18	9.0365	0.1923
12.05	12.05	29.1	contig00008_-_CyoA,_Heme/copper-type_cytochrome/quinol_oxidases,_subunit_2	10	8.8716	0.0436
14.13	14.13	56.7	contig00009_-_CspC,_Cold_shock_proteins	11	8.8716	0.247
4	4	23.7	contig00014_-_Uncharacterized_BCR,_YaiI/YqxD_family_COG1671	2	8.5507	0.1143
6.57	6.57	34.2	contig00006_-_Cytochrome_c	5	8.3946	0.1246
8	8	26	contig00017_-_thioredoxin_protein	6	8.3946	0.0523
8.49	8.49	24	contig00014_-_Chemotaxis_phosphatase,_CheZ	6	8.1658	0.009
6.18	6.18	13.6	contig00015_-_rho,_transcription_termination_factor_Rho	3	7.7983	0.2016
7.92	7.92	38.8	contig00005_-_Ttg2C,_ABC-type_transport_system_involved_in_resistance_to_organic_solvents,_periplasmic_component	4	7.5858	0.0393
4	4	29	contig00002_-_Universal_stress_protein_MJ0531	2	7.4473	0.427

2	2	9.4	contig00027_-_Thioredoxin-like_protein	2	7.4473	0.1148
17.71	17.71	28.4	contig00003_-_Periplasmic_binding_protein	16	7.379	0.0001
12.04	12.04	43.3	contig00017_-_Putative_lipoprotein	9	7.379	0.0288
2	2	8.5	contig00009_-_ZnuA,_ABC-type_Zn2+_transport_system,_periplasmic_component/surface_adhesin	2	7.1121	0.1899
8.86	8.92	24.1	contig00019_-_Outer_membrane_beta-barrel_domain_protein	7	7.0469	0.3211
2	2	11.1	contig00001_-_Cytochrome_c4	1	6.9183	0.1266
11.02	11.02	36.9	contig00015_-_Thioredoxin_protein	9	6.792	0.1011
2.51	2.51	19.4	contig00004_-_Sulfur_oxidation_protein_SoxY	2	6.7298	0.2199
16.05	16.05	41.1	contig00008_-_hypothetical_protein	12	6.6069	0.001
15.33	15.33	34	contig00003_-_preprotein_translocase_subunit_SecB	9	6.5464	0.0144
6	6	21.2	contig00030_-_nusG,_transcription_antitermination_protein_NusG	5	6.4863	0.0672
11.37	11.37	22.7	contig00011_-_HflK_protein	8	6.3096	0.0664
2.08	2.08	17.1	contig00016_-_AhpC/TSA_family	2	6.0813	0.0529
6	6	26.9	contig00001_-_Elongation_factor_Ts	4	6.0256	0.0855
2	2	21.7	contig00002_-_Protein-export_membrane_protein_SecG	1	6.0256	0.3026
20.36	20.36	35.6	contig00004_-_Chaperone_SurA	11	5.9704	0.0171
4.01	4.01	8.4	contig00007_-_Phosphomannomutase/phosphoglucomutase	2	5.9704	0.9668
4	4.01	27.5	contig00024_-_3-hydroxydecanoyl-(acyl_carrier_protein)_dehydratase	2	5.9704	0.3835
22.46	22.46	61.5	contig00056_-_Gram-negative_porin	47	5.9704	0.0389
39.73	39.73	20.3	contig00011_-_FimV,_Tfp_pilus_assembly_protein_FimV	26	5.8614	0.0032
4.75	4.75	16.1	contig00014_-_DdpA,_ABC-type_dipeptide_transport_system,_periplasmic_component	3	5.8076	0.0193
9.28	9.28	41.5	contig00006_-_30S_ribosomal_protein_S11	8	5.7544	0.2103
2.02	2.02	14.9	contig00002_-_OlmA,_Outer_membrane_lipoprotein_OmlA	2	5.7016	0.1398
2.31	2.32	17.9	contig00001_-_hypoxanthine-guanine_phosphoribosyltransferase	1	5.6494	0.2384
4.17	4.17	17.9	contig00002_-_Tetratricopeptide_TPR_2_repeat_protein	2	5.5463	0.382
2.3	2.3	20.5	contig00014_-_Flagellar_basal_body-associated_protein_FliL	1	5.2966	0.2154
13.15	13.15	36.5	contig00007_-_putative_outer_membrane_lipoprotein	11	5.2	0.1422
2	8	25	contig00005_-_Nitrogen_regulatory_protein_P-II	5	5.1523	0.2077
21.35	21.35	66.5	contig00001_-_frr,_ribosome_recycling_factor	15	5.1051	0
23.71	23.71	57.6	contig00006_-_rplF,_50S_ribosomal_protein_L6	20	5.1051	0.2703
6	6	21	contig00026_-_Predicted_redox_protein,_regulator_of_disulfide_bond_formation	4	5.0119	0.0616
8.19	8.19	33.9	contig00010_-_Membrane_protein_involved_in_aromatic_hydrocarbon_degradation	22	4.9204	0.0646
6.01	6.02	32.7	contig00006_-_ATPase_with_chaperone_activity,_ATP-binding_subunit	3	4.8753	0.1874
6.02	6.02	14.1	contig00001_-_rne,_ribonuclease_E	3	4.8306	0.1231
35.75	35.75	83.2	contig00026_-_Major_outer_membrane_protein_P.IB	66	4.8306	0.0063
6	6	25.8	contig00010_-_secreted_protein	3	4.7863	0.054
2	2.01	15.1	contig00011_-_Vi_polysaccharide_biosynthesis_protein_TviB	1	4.7424	0.7199
4.43	4.43	25.2	contig00002_-_Protein_GrpE	4	4.6989	0.2472
2	2	9.7	contig00001_-_ubiquinol-cytochrome_c_reductase,_iron-sulfur_subunit	1	4.6559	0.1576

3.05	3.05	37.9	contig00004_-_glycine_cleavage_system_protein_H	5	4.6559	0.3055
5.62	5.62	11.6	contig00005_-_argJ,_bifunctional_ornithine_acetyltransferase/N-acetylglutamate_synthase_protein	3	4.6132	0.0862
12.39	12.39	42.7	contig00007_-_Protein_PilG	7	4.529	0.0589
41.87	41.87	51.3	contig00002_-_dnaK,_molecular_chaperone_DnaK	33	4.4875	0
16	16	42.1	contig00013_-_Transmembrane_protein	9	4.4875	0.0138
2.43	2.43	34.3	contig00001_-_Lipoprotein	2	4.4055	0.2815
10.02	10.02	55.7	contig00001_-_3-ketoacyl-(Acyl-carrier-protein)_reductase	7	4.3652	0.0067
6.86	6.86	19.4	contig00007_-_Fimbrial_assembly_protein_PilQ	4	4.2855	0.1148
10.34	10.34	67.4	contig00016_-_DNA-directed_RNA_polymerase_subunit_omega	8	4.2073	0.6043
17.35	17.35	53.5	contig00001_-_tsf,_elongation_factor_Ts	14	4.1687	0.0209
13.55	13.55	71.6	contig00006_-_50S_ribosomal_protein_L29	9	4.1687	0.0044
8.45	10.79	15.4	contig00006_-_Outer_membrane_protein_assembly_factor_BamC	15	4.1687	0.1881
6.13	6.13	32.1	contig00005_-_Thiol:disulfide_interchange_protein_DsbA	3	4.1305	0.1722
5.44	5.44	26.7	contig00006_-_arsenate_reductase	5	4.1305	0.3177
2	2	3.6	contig00016_-_Sterol-binding_domain_protein	1	3.9446	0.304
2.25	2.26	17.7	contig00021_-_hypothetical_protein	1	3.9084	0.3745
2	2	9.4	contig00004_-_helix_turn_helix_multiple_antibiotic_resistance_protein	1	3.8726	0.4583
17.6	17.6	39.5	contig00006_-_LysM_domain/BON_superfamily_protein	34	3.8726	0.0104
23.4	23.4	30.2	contig00025_-_BtuB,_Outer_membrane_cobalamin_receptor_protein	19	3.8726	0.0033
31.17	32.54	35	contig00002_-_rpsA,_30S_ribosomal_protein_S1	19	3.8371	0.959
13.72	13.72	50.5	contig00004_-_Sulphur_oxidation_protein_SoxZ	16	3.8019	0.0032
7.13	7.13	13.8	contig00005_-_Fimbrial_protein_P9-2	11	3.767	0.1233
30.46	30.46	43.1	contig00002_-_Probable_parvulin-type_peptidyl-prolyl_cis-trans_isomerase	24	3.7325	0
2	2	9.5	contig00007_-_Protein_PilJ	1	3.7325	0.3969
8.85	8.85	28.5	contig00010_-_50S_ribosomal_protein_L9	6	3.7325	0.1385
2	2	23	contig00004_-_TolA_protein	2	3.6983	0.376
14	14	15.3	contig00024_-_Porins_form_aqueous_channels_for_the_diffusion_of_hydrophillic_molecules_across_the_outer_membrane	8	3.6983	0.0009
4.14	4.15	22.8	contig00002_-_Thioredoxin_domain-containing_protein	2	3.6644	0.054
3.85	3.85	21.2	contig00005_-_Cell_division_protein_ZapD	2	3.6644	0.1874
4	4	39.6	contig00007_-_Sec-independent_protein_translocase_protein_TatA	2	3.5645	0.2226
4	4	27.6	contig00026_-_2-nonaprenyl-3-methyl-6-methoxy-1,4-benzoquinol_hydroxylase	2	3.5645	0.2939
2.23	2.23	27	contig00002_-_Cupredoxin-like_domain	1	3.4995	0.2098
4.01	4.01	23.9	contig00006_-_50S_ribosomal_protein_L18	3	3.4995	0.2791
6.01	6.01	14	contig00015_-_Disulphide_bond_corrector_protein_DsbC	5	3.4674	0.2714
8.06	8.06	51.2	contig00001_-_NADPH-dependent_7-cyano-7-deazaguanine_reductase	4	3.4356	0.3071
4	4	17.9	contig00016_-_rpmE,_50S_ribosomal_protein_L31	3	3.4356	0.239
2.06	2.06	7	contig00015_-_dipZ,_thiol:disulfide_interchange_protein_precursor	1	3.4041	0.1969
2	2	9.4	contig00010_-_Probable_ATP-dependent_RNA_helicase_ddx27	2	3.3729	0.3186

2	2	2.5	contig00018_-_Outer_membrane_protein_beta-barrel_domain	1	3.3729	0.3103
6.03	6.03	14.2	contig00008_-_Outer_membrane_lipoprotein_Slp_family	4	3.3419	0.2492
8	8	24.8	contig00009_-_Conserved_hypothethical_protein,_UCP019302	4	3.3113	0.028
23.3	23.3	60.1	contig00010_-_30S_ribosomal_protein_S6	15	3.281	0.0003
4.15	4.15	23	contig00026_-_Bacterioferritin_protein	3	3.281	0.8767
10	10	32.9	contig00013_-_ndk,_mulitfunctional_nucleoside_diphosphate_kinase/apyrimidinic_endonuclease/3	10	3.2509	0.0291
4	6	23	contig00016_-_Putative_phosphoribosylformimino-5-aminoimidazole_carboxamide_ribotide_isomerase	3	3.2509	0.2619
6.77	6.77	14.5	contig00003_-_HemX,_Uncharacterized_enzyme_of_heme_biosynthesis	7	3.2211	0.0681
3.2	3.2	47.3	contig00015_-_50S_ribosomal_protein_L33	3	3.2211	0.2962
1.41	1.41	12.9	contig00003_-_GTPase_obg	1	3.1915	0.2062
12.74	12.74	26.3	contig00004_-_tol-pal_system_protein_YbgF	11	3.1915	0.0435
2	2	13.1	contig00007_-_glutamate_synthase_small_subunit_family_protein,_proteobacterial	1	3.1915	0.2533
6	6	10.2	contig00017_-_hypothetical_protein	3	3.1915	0.2699
12.12	12.12	15.8	contig00002_-_ATP-dependent_zinc_metalloprotease_FtsH	7	3.1046	0.0624
4	4	18.3	contig00001_-_PII-like_signaling_protein	2	3.0761	0.2379
2	2	4.1	contig00010_-_Outer_membrane_protein_transport_protein	1	3.0761	0.2125
2	2	11.7	contig00002_-_FHA_domain	4	3.0479	0.3015
2.38	2.38	19.2	contig00001_-_nudF,_ADP-ribose_pyrophosphatase_NudF	2	3.02	0.0397
1.92	2	4.7	contig00002_-_NADH-quinone_oxidoreductase_subunit_J	1	2.9648	0.3479
4.06	4.06	13.7	contig00003_-_Sporulation_related_domain	3	2.9648	0.347
2.07	2.08	7.5	contig00012_-_Asparaginase	2	2.9376	0.2197
2	2	10.2	contig00002_-_FabI,_Enoyl-[acyl-carrier-protein]	1	2.8576	0.2252
4.17	4.17	21.9	contig00002_-_oligoribonuclease	3	2.8314	0.0731
2.02	2.02	13.9	contig00006_-_signal_recognition_particle_protein	1	2.8314	0.2244
8.52	8.52	15.1	contig00016_-_tolC,_outer_membrane_channel_protein	10	2.8314	0.0607
10	10	53.3	contig00002_-_heat_shock_protein_GrpE	7	2.8054	0.0349
4	4	19	contig00005_-_UspA_domain_protein	3	2.8054	0.2887
2.02	2.02	14.3	contig00001_-_ATP-dependent_Clp_protease_adapter_protein_ClpS	1	2.7797	0.3465
2	2.01	13.3	contig00001_-_ihfA,_integration_host_factor_subunit_alpha	1	2.7797	0.1971
4	4	21.7	contig00015_-_hypothetical_protein	2	2.7797	0.3278
4.18	4.18	11.5	contig00016_-_ATP-dependent_protease_subunit_HslV	3	2.7797	0.2457
2	2	17.7	contig00011_-_Ttg2C,_ABC-type_transport_system_involved_in_resistance_to_organic_solvents,_periplasmic_component	1	2.7542	0.2331
20.56	20.56	48	contig00002_-_signal_peptidase_I	14	2.6792	0.0122
32.43	32.43	62.1	contig00006_-_Rubrerythrin_protein	66	2.6062	0.6951
10.7	10.7	28.6	contig00006_-_Uncharacterized_enzyme_involved_in_biosynthesis_of_extracellular_polysaccharides	12	2.5823	0.0727
4	4	6.9	contig00020_-_Succinate-semialdehyde_dehydrogenase_[NADP(+)]_GabD	3	2.5823	0.2795
2.28	2.28	19.8	contig00003_-_30S_ribosomal_protein_S20	2	2.5119	0.2546
4.57	4.57	24.9	contig00007_-_Pilus_assembly_protein,_PilP	3	2.5119	0.3138
14.7	14.7	30.8	contig00004_-_tolB,_translocation_protein_TolB	14	2.466	0.0104

4.05	4.05	18.2	contig00008_-_ubiquinol-cytochrome_c_reductase,_iron-sulfur_subunit	2	2.4434	0.1543
8.71	8.71	14.6	contig00025_-_5-methyltetrahydropteroyltriglutamate--_homocysteine_S-methyltransferase	6	2.355	0.6912
4	4	11.9	contig00009_-_glycine_dehydrogenase_subunit_2	2	2.3335	0.0543
2	4.01	8	contig00009_-_Hemolysin_secretion_protein	2	2.2699	0.2822
2	2	7.6	contig00003_-_Bacterial_protein_of_unknown_function_(DUF945)	1	2.2491	0.2826
10.71	10.85	22.1	contig00001_-_Ubiquinol_oxidase_subunit_2	9	2.208	0.2744
14	14	48.1	contig00016_-_oxidative_damage_protection_protein	16	2.208	0.1501
2	2	13.3	contig00004_-_YdcF-like_protein	1	2.1878	0.2843
6	6	25.6	contig00005_-_YfaZ_family_protein	5	2.1878	0.2164
2.74	2.74	8.8	contig00007_-_DNA_topoisomerase_I	2	2.1281	0.3939
4	4	26	contig00011_-_Predicted_peroxiredoxins	10	2.1281	0.3141
4.63	4.63	12.6	contig00003_-_PKD_domain_containing_protein	3	2.1086	0.0967
2	2	11.8	contig00009_-_iron-sulfur_cluster_insertion_protein_ErpA	2	2.1086	0.3048
4.03	4.03	17.2	contig00016_-_rph,_ribonuclease_PH	2	2.1086	0.3158
2	2	3.5	contig00022_-_Probable_transcriptional_regulator_LumQ	1	2.1086	0.3029
2	2	18	contig00001_-_Acyl_carrier_protein	1	2.0893	0.3242
2	2	7.9	contig00005_-_Sigma_54_modulation_protein_/_S30EA_ribosomal_protein	1	2.0893	0.3476
2.05	2.05	13.2	contig00006_-_Uncharacterized_conserved_secreted_or_membrane_protein	2	2.0701	0.3671
2.01	2.01	9.6	contig00001_-_Predicted_transcriptional_regulator	1	2.0512	0.3143
4	4	30.1	contig00006_-_Putative_lipoprotein	2	2.0324	0.2626
2	2.01	7.9	contig00011_-_tryptophan_synthase_subunit_beta	1	2.0137	0.2726
4.11	4.11	23.7	contig00007_-_Yqey-like_protein	5	1.9953	0.3576
2.02	2.02	5	contig00016_-_dihydrolipoamide_dehydrogenase	1	1.9953	0.3241
2.33	2.33	8.5	contig00025_-_Cobalamin_adenosyltransferase	2	1.9588	0.3325
1.74	2	9.2	contig00012_-_gatA,_aspartyl/glutamyl-tRNA_amidotransferase_subunit_A	1	1.9409	0.4118
5.77	5.8	43.9	contig00005_-_CopC_domain	6	1.9231	0.1035
8.25	8.25	37.3	contig00001_-_hypothetical_protein	5	1.9055	0.8475
4.12	4.12	28.6	contig00001_-_putative_nucleotide-binding_protein	3	1.8707	0.1771
10	10	64.8	contig00006_-_rplX,_50S_ribosomal_protein_L24	8	1.8707	0.5836
2	2	3	contig00002_-_cmk,_cytidylate_kinase	1	1.8535	0.3563
2.26	4.2	9.3	contig00012_-_efflux_transporter,_outer_membrane_factor_(OMF)_lipoprotein,_NodT_family	4	1.8535	0.3544
6.22	6.22	27.8	contig00005_-_phosphoheptose_isomerase	7	1.8365	0.1172
4.47	4.47	15.9	contig00011_-_L,D-transpeptidase_catalytic_domain	4	1.8197	0.147
4.05	4.05	13	contig00002_-_Cytidylate_kinase-like_family	2	1.803	0.3771
3.92	4	34.4	contig00001_-_Trm112p-like_protein	2	1.7865	0.4457
2.01	2.01	9.9	contig00010_-_dihydroxy-acid_dehydratase	1	1.7701	0.3755
2	17.88	41.7	contig00027_-_Gram-negative_porin	28	1.7701	0.3623
8.26	8.26	8.8	contig00006_-_Zn-finger_containing_NTP_pyrophosphohydrolase	4	1.7539	0.5771
6.01	6.01	43.6	contig00020_-_Bcp,_Peroxiredoxin	3	1.7539	0.4673

8.01	8.01	19.9	contig00001_-_FabD,_(acyl-carrier-protein)_S-malonyltransferase	6	1.7378	0.5561
8.54	8.54	33.9	contig00006_-_single-stranded_DNA-binding_protein	5	1.7219	0.0731
2	2	12.3	contig00017_-_Thioredoxin_domain-containing_protein	1	1.7219	0.3945
4.78	4.78	52.8	contig00001_-_infA,_translation_initiation_factor_IF-1	3	1.7061	0.3076
4	4	24.8	contig00006_-_iojap-like_ribosome-associated_protein	2	1.6904	0.2865
2.03	2.03	12.2	contig00017_-_PleD,_Response_regulator_containing_a_CheY-like_receiver_domain_and_a_GGDEF_domain	2	1.6904	0.2912
16	16	23.3	contig00027_-_Dienelactone_hydrolase_and_related_enzymes	12	1.6904	0.8167
2.48	2.48	16.5	contig00004_-_Protein_TolB	2	1.6749	0.549
2	2	14.5	contig00005_-_Bacterial_cellulose_synthase_subunit	1	1.6749	0.4099
2	2	19.8	contig00007_-_hisE,_phosphoribosyl-ATP_pyrophosphatase	1	1.6749	0.4085
2	2	4.7	contig00002_-_Ureidoglycolate_lyase	1	1.6596	0.3681
2.32	2.32	45.1	contig00006_-_rplN,_50S_ribosomal_protein_L14	2	1.6444	0.354
5.31	5.31	18.7	contig00016_-_Curved_DNA-binding_protein	3	1.6444	0.5634
13.08	13.08	21.1	contig00036_-_cysteine_desulfurase	7	1.6444	0.5416
7.6	7.6	15	contig00017_-_dihydrolipoamide_acetyltransferase	5	1.6293	0.4717
2	2	4.9	contig00001_-_UDP-N-acetylglucosamine_acyltransferase	1	1.6144	0.4318
4.92	4.92	7.7	contig00010_-_pyruvate_dehydrogenase	3	1.6144	0.1934
2.01	2.01	8.7	contig00013_-_hscB,_co-chaperone_HscB	1	1.5996	0.3722
2	2	24.2	contig00002_-_Signal_peptidase_I	2	1.5849	0.5195
6.01	6.01	17.3	contig00012_-_gatB,_aspartyl/glutamyl-tRNA_amidotransferase_subunit_B	3	1.5849	0.4649
5.96	6	11.8	contig00013_-_Putative_methyl-accepting_chemotaxis_AlkN	3	1.5849	0.3063
2	2	8.5	contig00015_-_moaA,_molybdenum_cofactor_biosynthesis_protein_A	1	1.5849	0.3928
2.47	2.47	9.6	contig00007_-_Hpt_domain	2	1.556	0.208
8.08	8.08	18.9	contig00026_-_Protein_CbbQ	5	1.556	0.9069
2	2	11.3	contig00010_-_Oxidoreductase,_aldo/keto_reductase_family	1	1.5417	0.5157
23.27	23.27	37.9	contig00002_-_Trigger_factor	25	1.5276	0.532
4	4	8.3	contig00020_-_Outer_membrane_beta_barrel_protein	2	1.5276	0.3741
3.81	3.81	15.8	contig00006_-_tldD,_protease_TldD	3	1.5136	0.5571
1.82	2	14.3	contig00002_-_PilZ_domain	1	1.4997	0.4823
4.12	4.14	24.8	contig00006_-_This_family_is_most_closely_related_to_the_GT1_family_of_glycosyltransferases	3	1.4997	0.369
4	4	25.8	contig00022_-_carboxysome_peptide_A	5	1.4997	0.4687
2.24	2.24	19.2	contig00004_-_Cytochrome_C_oxidase,_cbb3-type,_subunit_III	2	1.4859	0.6008
1.92	2	10.9	contig00007_-_Putative_cell_wall_biogenesis_protein	1	1.4859	0.4189
3.64	3.76	14.7	contig00017_-_dihydrolipoamide_dehydrogenase	2	1.4859	0.7718
4.74	4.74	11.6	contig00002_-_Translation_initiation_factor_IF-2	3	1.4723	0.3262
4	4	6.2	contig00016_-_periplasmic_serine_protease,_Do/DeqQ_family	2	1.4723	0.4587
4.02	4.02	14.7	contig00035_-_TolQ,_Biopolymer_transport_proteins	2	1.4588	0.3719
2	2	8.1	contig00009_-_Rhodanese_Homology_Domain	1	1.4454	0.3955
3.82	3.82	5.6	contig00003_-_ATP_synthase_epsilon_chain	2	1.4322	0.5057

2.01	4.03	10.5	contig00010_-_Hemolysin_secretion_protein	3	1.4322	0.5157
6.01	6.01	23.9	contig00011_-_FtsH_protease_regulator_HflC	5	1.4322	0.281
3.59	3.59	25.9	contig00004_-_Phospholipid-binding_protein	2	1.4191	0.8831
6	6.01	42.2	contig00006_-_rpsS,_30S_ribosomal_protein_S19	6	1.4191	0.1431
4	4	9.9	contig00007_-_Orotate_phosphoribosyltransferase	3	1.3932	0.4015
1.48	1.48	6.8	contig00017_-_Carboxymuconolactone_decarboxylase	1	1.3932	0.5408
2	2	9.1	contig00002_-_Protein_YciI	1	1.3677	0.5064
2.02	2.02	2.8	contig00003_-_c-di-GMP_phosphodiesterase_class_II	1	1.3677	0.5619
7.03	9.31	17.8	contig00003_-_DNA_polymerase_III_subunit_beta	6	1.3552	0.8728
26.61	26.61	29.8	contig00003_-_F0F1_ATP_synthase_subunit_beta	22	1.3552	0.7563
4	4	16.9	contig00005_-_PTS_IIA-like_nitrogen-regulatory_protein_PtsN	2	1.3552	0.6052
4	4	5.4	contig00003_-_hemC,_porphobilinogen_deaminase	2	1.3428	0.6101
2.16	2.16	26.8	contig00014_-_Tetratricopeptide_TPR_1_repeat-containing_protein	1	1.3428	0.048
4.37	4.37	24.5	contig00010_-_nusB,_transcription_antitermination_protein_NusB	2	1.3305	0.2314
5.1	5.1	39.9	contig00015_-_Dihydroneopterin_aldolase	3	1.3305	0.0466
2.04	2.04	11.3	contig00017_-_ABC_transporter,_phosphonate,_periplasmic_substrate-binding_protein	2	1.3183	0.4322
4	4	37.2	contig00030_-_Elongation_factor_G_C-terminus	3	1.3183	0.6772
2	2	8.6	contig00004_-_Lipoate_regulatory_protein_YbeD	1	1.3062	0.6102
2	2	5.1	contig00005_-_Cell_division_protein_FtsA	1	1.3062	0.6061
2.79	2.79	50.8	contig00006_-_rpmD,_50S_ribosomal_protein_L30	3	1.2942	0.6931
3.62	3.62	16.2	contig00026_-_Cytochrome_c553	2	1.2942	0.6155
1.92	2	11.4	contig00002_-_Scavenger_mRNA_decapping_enzyme_C-term_binding	1	1.2706	0.7285
2	2	7.2	contig00011_-_ABC-type_uncharacterized_transport_system,_auxiliary_component	1	1.2706	0.6387
4.06	4.06	14.3	contig00021_-_inositol_monophosphatase	3	1.2706	0.7203
6	6	10	contig00006_-_peptidase_PmbA	3	1.2589	0.5325
2.98	2.98	14.8	contig00007_-_DNA_topoisomerase_1	2	1.2589	0.3209
38.78	38.78	37.1	contig00008_-_Putative_outer_membrane_cytochrome_c	50	1.2589	0.135
3.28	3.28	14.4	contig00016_-_dapF,_diaminopimelate_epimerase	2	1.2589	0.5456
2	2	11.1	contig00025_-_AmpD_protein	1	1.2359	0.4208
4.01	4.01	14.4	contig00006_-_rbcR,_LysR_transcriptional_regulator	2	1.2246	0.94
2	2	10.2	contig00007_-_Hemerythrin_HHE_cation_binding_domain	2	1.2246	0.6334
2	2	25.3	contig00013_-_Chaperone_protein_HscA_homolog	1	1.2246	0.7979
2.83	2.83	13.6	contig00007_-_pyrroline-5-carboxylate_reductase	2	1.2134	0.4662
4	4	30.2	contig00013_-_RNA_recognition_motif	2	1.2023	0.7103
1.6	1.6	9.7	contig00003_-_branched-chain_amino_acid_aminotransferase	1	1.1803	0.7382
2	2.03	7	contig00002_-_Predicted_3'-5'_exonuclease_related_to_the_exonuclease_domain_of_PolB	1	1.1695	0.7465
5.82	5.82	19.8	contig00005_-_RbsK,_Sugar_kinases,_ribokinase_family	3	1.1695	0.5318
4	4.29	36.9	contig00026_-_rpsI,_30S_ribosomal_protein_S9	3	1.1588	0.6787
2	2	11.3	contig00026_-_Trypsin-like_peptidase_domain	1	1.1588	0.71

4.05	4.05	18	contig00003_-_F0F1_ATP_synthase_subunit_delta	2	1.1482	0.9093
2	2	5.2	contig00012_-_SlpA,_FKBP-type_peptidyl-prolyl_cis-trans_isomerases_2	3	1.1482	0.415
2	2	2.4	contig00004_-_Tetratricopeptide_TPR_4	1	1.1376	0.7892
4.02	4.02	29.4	contig00014_-_short_chain_dehydrogenase	3	1.1376	0.4656
2	2	7.5	contig00004_-_Ferredoxin_1	1	1.1272	0.8536
7.07	7.07	24.1	contig00004_-_ferredoxin-NADP_reductase	4	1.1272	0.6086
4.9	4.93	29.9	contig00004_-_Putative_lipoprotein	3	1.1272	0.4448
7.89	7.89	37.2	contig00022_-_carboxysome_peptide_B	4	1.1272	0.9964
1.92	2	4.4	contig00002_-_PTC1,_Serine/threonine_protein_phosphatase	1	1.1169	0.8159
1.57	1.57	17.7	contig00026_-_Ferredoxin,_2Fe-2S	1	1.1169	0.814
2	2	5.2	contig00002_-_N-ethylammeline_chlorohydrolase	1	1.0965	0.817
4	4	24.2	contig00002_-_ihfB,_integration_host_factor_subunit_beta	2	1.0864	0.6312
4.28	4.28	50.6	contig00003_-_rpmA,_50S_ribosomal_protein_L27	3	1.0864	0.8591
2	2.01	14.3	contig00007_-_Competence_protein_A	1	1.0765	0.9987
2	2	3.6	contig00014_-_Hydrogen_peroxide-inducible_genes_activator	1	1.0765	0.8966
2	2	10.4	contig00015_-_acetyl-CoA_carboxylase_biotin_carboxylase_subunit	1	1.0765	0.8612
4	4	19.1	contig00017_-_minE,_cell_division_topological_specificity_factor_MinE	2	1.0765	0.7656
4	4	17.3	contig00002_-_DsrE/DsrF-like_family	3	1.0666	0.7928
13.32	13.32	22.3	contig00004_-_Multidrug_resistance_outer_membrane_protein_MdtP	7	1.0666	0.6796
2	2	19.3	contig00004_-_PspE,_Rhodanese-related_sulfurtransferase	1	1.0666	0.9006
2.01	2.01	12	contig00026_-_Bacterial_microcompartments_are_primitive_organelles_composed_entirely_of_protein_subunits	2	1.0568	0.7073
2	2	4.8	contig00020_-_periplasmic_serine_protease,_Do/DeqQ_family	1	1.0471	0.904
6.02	6.02	27.5	contig00002_-_elongation_factor_P	3	1.0375	0.5119
6.08	6.18	17	contig00008_-_AfuA,_ABC-type_Fe3+_transport_system,_periplasmic_component	6	1.0375	0.7108
2	2	4.7	contig00003_-_6-phosphofructokinase	1	1.028	0.9356
2	2	5.5	contig00004_-_LPS-assembly_protein_LptD	1	1.028	0.7194
15.27	15.27	12.5	contig00001_-_Phosphoesterase_family	9	1.0186	0.7552
9.75	9.75	43.2	contig00006_-_rpsH,_30S_ribosomal_protein_S8	8	1.0186	0.9243
12	12	27.3	contig00030_-_50S_ribosomal_protein_L11	6	1.0186	0.9229
13.6	13.6	23.4	contig00007_-_LysM_domain/BON_superfamily_protein	7	1.0093	0.5803
2	2	21.4	contig00015_-_CopZ,_Copper_chaperone	1	1.0093	0.5817
6.01	6.01	13.3	contig00008_-_Cytochrome_c4	4	1	0.7758
4	4	8.6	contig00009_-_glycine_dehydrogenase_subunit_1	4	1	0.7058
2	2	13.8	contig00009_-_Phosphoesterase_family	1	1	0.4083
2	2	6.9	contig00015_-_purH,_bifunctional_phosphoribosylaminoimidazolecarboxamide_formyltransferase/IMP_cyclohydrolase	1	1	0.9867
7.62	7.62	12.2	contig00005_-_CirA,_Outer_membrane_receptor_proteins,_mostly_Fe_transport	5	0.9908	0.6031
2	2.01	8.4	contig00009_-_histidinol-phosphatase,_inositol_monophosphatase_family	1	0.9908	0.8938
4	4	24.7	contig00026_-_rplM,_50S_ribosomal_protein_L13	3	0.9908	0.988
5.31	5.31	41.3	contig00009_-_molybdopterin_biosynthesis_protein_MoeB	5	0.9817	0.5473

1.68	1.68	13.5	contig00014_-_ilvH,_acetolactate_synthase_3_regulatory_subunit	1	0.9817	0.7683
8.15	8.15	55.8	contig00006_-_rpsJ,_30S_ribosomal_protein_S10	5	0.9638	0.9047
10	10	24.2	contig00030_-_rplA,_50S_ribosomal_protein_L1	6	0.9638	0.7603
2	2	12.2	contig00015_-_WcaG,_Nucleoside-diphosphate-sugar_epimerases	1	0.955	0.943
6.88	6.88	20.9	contig00026_-_ribulose-phosphate_3-epimerase	4	0.955	0.5979
3	3	9.3	contig00002_-_Segregation_and_condensation_protein_B_homolog	2	0.9462	0.1179
2	2.01	12.8	contig00003_-_helix_turn_helix,_Arsenical_Resistance_Operon_Repressor	1	0.929	0.9553
2	2	12	contig00001_-_pyrH,_uridylate_kinase	1	0.9204	0.875
5.35	5.35	8.6	contig00003_-_phosphoenolpyruvate_synthase	3	0.9204	0.8342
21.56	21.59	45.3	contig00004_-_HdrA,_Heterodisulfide_reductase,_subunit_A_and_related_polyferredoxins	14	0.9036	0.7025
11.73	11.73	42.6	contig00006_-_rpsE,_30S_ribosomal_protein_S5	8	0.9036	0.2665
2.05	2.05	12.5	contig00010_-_recombination_and_DNA_strand_exchange_inhibitor_protein	1	0.9036	0.741
2.11	2.11	9.2	contig00020_-_Ferredoxin--NADP_reductase	1	0.9036	0.7937
28.36	28.36	16.1	contig00030_-_DNA-directed_RNA_polymerase_subunit_beta	16	0.9036	0.2599
6	6	14.5	contig00002_-_tpiA,_triosephosphate_isomerase	3	0.8954	0.1375
2	2	8.3	contig00009_-_glycine_cleavage_system_protein_H	2	0.8954	0.9405
3.89	3.89	11.7	contig00006_-_putative_GTP_cyclohydrolase	2	0.8872	0.316
2	2	5.3	contig00009_-_NAD(P)H:quinone_oxidoreductase	1	0.8872	0.7428
3.62	3.63	14.8	contig00007_-_RNA_polymerase_sigma_factor_RpoD	2	0.879	0.6194
2	2	6.4	contig00026_-_Putative_transmembrane_protein_coupled_to_NADH-ubiquinone_oxidoreductase_chain_5-like_protein	1	0.879	0.81
13.19	13.19	34.1	contig00006_-_rplC,_50S_ribosomal_protein_L3	8	0.871	0.2932
4	4	16.6	contig00012_-_EVE_domain	2	0.871	0.7091
7.48	7.48	25.5	contig00018_-_Gram-negative_porin	7	0.863	0.7274
3.29	5.56	14.9	contig00001_-_clpA,_ATP-dependent_Clp_protease_ATP-binding_subunit	3	0.8551	0.3884
6	6.59	33.7	contig00002_-_SirA-like_protein	3	0.8472	0.2291
3.39	3.39	46.9	contig00008_-_Membrane_fusogenic_activity	5	0.8472	0.773
2	2	16.3	contig00001_-_lipoprotein_releasing_system,_ATP-binding_protein	1	0.8395	0.8523
2.1	2.1	11.6	contig00012_-_prfA,_peptide_chain_release_factor_1	1	0.8318	0.653
2	2.01	6.8	contig00020_-_Curved_DNA-binding_protein	1	0.8318	0.8052
17.76	17.76	18.1	contig00001_-_polynucleotide_phosphorylase/polyadenylase	9	0.8166	0.4265
2.12	2.12	11.2	contig00004_-_antiporter_inner_membrane_protein	1	0.8166	0.6914
10.97	10.97	18	contig00005_-_Thiazole_synthase	8	0.8166	0.9925
3.47	3.56	12.7	contig00016_-_VacJ,_Surface_lipoprotein	2	0.8166	0.4073
2.01	2.01	13.7	contig00007_-_bifunctional_pyrimidine_regulatory_protein_PyrR_uracil_phosphoribosyltransferase	1	0.8091	0.5473
2	2	6.2	contig00009_-_tyrosyl-tRNA_synthetase	1	0.8091	0.8879
6	6.01	11.9	contig00013_-_outer_membrane_assembly_lipoprotein_YfgL	7	0.8091	0.5746
2	2	3.4	contig00027_-_Tas,_Predicted_oxidoreductases	1	0.8091	0.6961
4	4	11.1	contig00005_-_Putative_NADH_dehydrogenase/NAD(P)H_nitroreductase_AF_0226	2	0.8017	0.7586
11.18	11.43	16	contig00005_-_ResB-like_family	9	0.8017	0.3748

2.03	2.03	4.8	contig00006_-_purT,_phosphoribosylglycinamide_formyltransferase_2	1	0.8017	0.6764
2	2.01	9.8	contig00006_-_prolyl-tRNA_synthetase	1	0.7943	0.1068
2	2	28.2	contig00007_-_30S_ribosomal_protein_S21	1	0.7943	0.7092
5.35	5.35	9.1	contig00003_-_putative_quinone_oxidoreductase,_YhdH/YhfP_family	3	0.787	0.7115
1.57	1.57	8.3	contig00006_-_RfbB,_dTDP-D-glucose_4,6-dehydratase	1	0.787	0.6584
2	2	3.4	contig00003_-_argininosuccinate_lyase	1	0.7798	0.6415
2	2	5.8	contig00004_-_Enolase-phosphatase_E1	1	0.7798	0.6509
2	2	12.2	contig00011_-_trpA,_tryptophan_synthase_subunit_alpha	1	0.7798	0.6471
2	2	1.6	contig00016_-_dihydrolipoamide_succinyltransferase	1	0.7798	0.6492
6.45	6.45	36.3	contig00030_-_rpsL,_30S_ribosomal_protein_S12	5	0.7798	0.5318
2	2.01	10.8	contig00002_-_Carbamoyl-phosphate_synthase_small_chain,_CPSase_domain	1	0.7727	0.4409
2	2	4.9	contig00012_-_Uncharacterized_Fe-S_protein	1	0.7727	0.6407
7.38	7.38	13.7	contig00002_-_acetyl-CoA_synthetase	4	0.7586	0.1956
6.84	6.84	16.9	contig00003_-_pntA,_NAD(P)_transhydrogenase_subunit_alpha	4	0.7586	0.1295
4	4	4.5	contig00006_-_glpC,_sn-glycerol-3-phosphate_dehydrogenase_subunit_C	2	0.7586	0.6234
2	2.01	4.3	contig00007_-_Voltage_gated_chloride_channel	1	0.7586	0.8705
4	4.04	8.2	contig00015_-_BisC,_Anaerobic_dehydrogenases,_typically_selenocysteine-containing	2	0.7516	0.6917
4.01	4.03	12.2	contig00001_-_fabG,_3-ketoacyl-(acyl-carrier-protein)_reductase	2	0.7379	0.3449
2.01	2.01	7.3	contig00004_-_Dyp-type_peroxidase_family	1	0.7379	0.2227
2.26	2.26	14.4	contig00022_-_ATP-dependent_dethiobiotin_synthetase_BioD	2	0.7379	0.6649
4	4	11.3	contig00005_-_succinyl-CoA_synthetase_subunit_alpha	3	0.7311	0.5344
6	6.01	23.6	contig00003_-_OmpR,_Response_regulators_consisting_of_a_CheY-like_receiver_domain_and_a_DNA-binding_domain	3	0.7244	0.5934
6	6	12.2	contig00004_-_HdrC,_Heterodisulfide_reductase,_subunit_C	4	0.7244	0.1391
2.05	2.05	4.7	contig00005_-_aspS,_aspartyl-tRNA_synthetase	1	0.7244	0.1679
6	6	17.8	contig00012_-_Cytochrome_C	3	0.7112	0.3385
2	2	6.3	contig00006_-_PhoH,_Phosphate_starvation-inducible_protein_PhoH,_predicted_ATPase	1	0.7047	0.4857
2.04	2.04	27.4	contig00001_-_50S_ribosomal_protein_L32	1	0.6982	0.5291
3.03	3.03	21.1	contig00006_-_rplS,_50S_ribosomal_protein_L19	3	0.6982	0.3334
4	4	6.4	contig00003_-_ileS,_isoleucyl-tRNA_synthetase	2	0.6918	0.0468
2	2	19	contig00012_-_Aspartyl/glutamyl-tRNA(Asn/Gln)_amidotransferase_subunit_C	1	0.6855	0.6938
2	2	7.6	contig00011_-_chorismate_synthase	1	0.6792	0.5085
7.79	7.79	13.9	contig00002_-_nusA,_transcription_elongation_factor_NusA	4	0.673	0.6249
4.53	4.63	12.6	contig00006_-_Lpd,_Pyruvate/2-oxoglutarate_dehydrogenase_complex,_dihydrolipoamide_dehydrogenase_(E3)_component	2	0.673	0.5112
4.01	4.01	14.5	contig00010_-_Nucleoprotein/polynucleotide-associated_enzyme	2	0.673	0.2439
14.28	14.28	11.2	contig00001_-_aconitate_hydratase	8	0.6668	0.3492
8.11	8.11	27.6	contig00004_-_transaldolase	6	0.6668	0.11
19.7	19.7	30.4	contig00022_-_pyruvate_kinase	17	0.6668	0.3083
2	2	24.3	contig00036_-_Cysteine_desulfhydrase_/_Selenocysteine_lyase	1	0.6668	0.4991

8.14	8.14	32.6	contig00009_-_tpx,_lipid_hydroperoxide_peroxidase	7	0.6607	0.6942
4.37	5.07	25.2	contig00011_-_leuD,_isopropylmalate_isomerase_small_subunit	3	0.6546	0.0429
2	2	6.5	contig00022_-_RuBisCO_operon_transcriptional_regulator	1	0.6427	0.4692
3.78	3.78	40.7	contig00010_-_Putative_cytoplasmic_protein	2	0.6368	0.4597
6	6.09	12.7	contig00017_-_aceE,_pyruvate_dehydrogenase_subunit_E1	3	0.6368	0.3062
2.03	2.03	13.6	contig00001_-_General_stress_protein_69	1	0.631	0.2774
15.27	15.27	15.6	contig00021_-_Protein_translocase_subunit_SecD	8	0.631	0.5343
2	2	8.8	contig00006_-_phosphoribosylaminoimidazole_synthetase	1	0.6252	0.4477
8	8	18.5	contig00005_-_outer_membrane_assembly_lipoprotein_YfiO	4	0.6194	0.1662
3.8	3.8	14.8	contig00002_-_Pterin_4_alpha_carbinolamine_dehydratase	2	0.6138	0.4043
2	2	10.4	contig00002_-_Predicted_kinase	1	0.6081	0.431
2.14	2.15	5.5	contig00006_-_AcrB,_Cation/multidrug_efflux_pump	1	0.6081	0.4197
4.22	4.22	11.5	contig00002_-_pyridoxine_5'-phosphate_synthase	2	0.6026	0.6456
2	2.03	5.3	contig00005_-_LppC_putative_lipoprotein	2	0.6026	0.4249
2	2	6.3	contig00007_-_Cyclic_nucleotide-binding_domain	1	0.6026	0.4023
2.36	2.37	5.6	contig00002_-_clpX,_ATP-dependent_protease_ATP-binding_subunit_ClpX	1	0.5861	0.4105
2	2	18.4	contig00016_-_PAS_sensor_protein	1	0.5861	0.3727
4	4	8	contig00012_-_GTP-binding_protein_YchF	4	0.5754	0.3047
3.05	3.09	9.4	contig00003_-_glyS,_glycyl-tRNA_synthetase_subunit_beta	2	0.5649	0.5829
2	2	6.1	contig00002_-_3-phosphoshikimate_1-carboxyvinyltransferase	1	0.5598	0.3822
4	4	6.1	contig00002_-_mltD,_membrane-bound_lytic_murein_transglycosylase_D	3	0.5598	0.3835
68.35	68.35	46.4	contig00002_-_NADH_dehydrogenase	48	0.5598	0.6891
2.29	2.29	16.2	contig00008_-_SCO1_protein_homolog	2	0.5546	0.3664
4	4	19	contig00005_-_ManX,_Phosphotransferase_system,_mannose/fructose-specific_component_IIA	2	0.5495	0.3661
2	2	4.4	contig00011_-_Peptidase_C26	1	0.5495	0.3743
9.91	9.91	20.9	contig00012_-_RND_family_efflux_transporter,_MFP_subunit	8	0.5445	0.6185
9.5	9.58	21.1	contig00011_-_HflC_protein	5	0.5395	0.8826
2	2	5.3	contig00010_-_heavy_metal_response_regulator	1	0.5346	0.3599
4.01	4.01	24.4	contig00004_-_Penicillin-binding_protein_5	2	0.5248	0.3846
4.8	4.8	42.7	contig00026_-_Putative_pterin-4_alpha-carbinolamine_dehydratase-like_protein	5	0.5248	0.4177
2	2	18.5	contig00003_-_ispH,_4-hydroxy-3-methylbut-2-enyl_diphosphate_reductase	1	0.52	0.3514
2	2	24.8	contig00002_-_Dienelactone_hydrolase_family	1	0.5152	0.2421
2	4	4.8	contig00005_-_Transcriptional_regulator	2	0.5152	0.3451
2.06	2.06	5.5	contig00001_-_NMT1-like_family	1	0.5105	0.3393
12.11	12.11	41.1	contig00004_-_Acireductone_dioxygenase	7	0.5105	0.2318
2	2	6.9	contig00012_-_GntR,_Transcriptional_regulators	1	0.5105	0.338
3.92	4	7.2	contig00003_-_dihydroorotate_dehydrogenase_1B	2	0.5058	0.194
1.68	1.68	6.3	contig00013_-_lysS,_lysyl-tRNA_synthetase	1	0.5058	0.2981
8.04	8.04	18.4	contig00006_-_RND_family_efflux_transporter,_MFP_subunit	5	0.4875	0.0235

2.01	2.02	4.7	contig00012_-_multidrug_efflux_protein	1	0.4875	0.3288
2	2	6.5	contig00001_-_ferrochelatase	1	0.4742	0.2894
8.12	8.12	27.7	contig00001_-_Predicted_peroxiredoxins	7	0.4699	0.3416
4	4	43.2	contig00006_-_50S_ribosomal_protein_L15	2	0.4699	0.3061
2.01	2.01	13.9	contig00022_-_N5-carboxyaminoimidazole_ribonucleotide_mutase	1	0.4656	0.2857
6	6.01	12.1	contig00022_-_transketolase	5	0.4613	0.086
12.33	12.33	22.1	contig00004_-_HdrB,_Heterodisulfide_reductase,_subunit_B	12	0.4529	0.2573
26.72	26.72	43.9	contig00003_-_phosphoribulokinase	20	0.4446	0.0138
3.54	3.58	3.9	contig00007_-_phosphoglyceromutase	2	0.4406	0.2383
12	12	29.3	contig00017_-_transcriptional_regulator_PhoU	8	0.4406	0.9741
2	2	6.4	contig00006_-_Chaperone_protein_HtpG	1	0.4365	0.285
6.13	6.13	20.6	contig00006_-_Fe-S_oxidoreductase-like_protein	3	0.4325	0.2951
2.36	2.36	6.3	contig00022_-_S-adenosylmethionine_synthetase	1	0.4169	0.2231
4.12	4.12	11.6	contig00001_-_TolC,_Outer_membrane_protein	2	0.4093	0.2634
2	2	4.6	contig00001_-_3-oxoacyl-(acyl_carrier_protein)_synthase_III	1	0.4055	0.2637
2	2	18.2	contig00002_-_RsuA,_16S_rRNA_uridine-516_pseudouridylate_synthase_and_related_pseudouridylate_synthases	1	0.4055	0.2631
2	2	10.2	contig00006_-_Glycine-zipper_containing_OmpA-like_membrane_domain	2	0.4055	0.5216
2.02	2.02	8.4	contig00018_-_Protein_RcaC	1	0.4055	0.2238
2	2	16.7	contig00002_-_Escherichia_coli_YhbY_is_associated_with_pre-50S_ribosomal_subunits,_ribosome_assembly	1	0.4018	0.3803
2.32	2.32	5.9	contig00012_-_TPR_repeat-containing_protein_PA4667	1	0.4018	0.734
14.02	14.02	29.1	contig00003_-_F0F1_ATP_synthase_subunit_alpha	10	0.3981	0.5399
2	2	5.2	contig00017_-_Chemotaxis_protein_CheY	1	0.3981	0.2627
2	2	4.6	contig00006_-_LPS-assembly_lipoprotein_LptE	1	0.3945	0.2563
6	6	10.5	contig00007_-_1-(5-phosphoribosyl)-5-[(5-_phosphoribosylamino)methylideneamino]_imidazole-4-carboxamide_isomerase	3	0.3873	0.0143
2.02	2.03	39.7	contig00006_-_50S_ribosomal_protein_L17	1	0.3837	0.2498
6	6	6	contig00009_-_Protein_HelA	3	0.3802	0.0615
4.02	4.02	9.2	contig00002_-_DNA_gyrase_subunit_A	2	0.3767	0.2377
2.02	2.02	4.9	contig00010_-_glutamate_decarboxylase	1	0.3767	0.2457
2.2	2.2	8.2	contig00004_-_aspartate_kinase	2	0.3664	0.1916
31.82	31.82	52.7	contig00044_-_elongation_factor_Tu	33	0.3664	0.2337
2	2	11.3	contig00007_-_ATPase_components_of_ABC_transporters_with_duplicated_ATPase_domains	1	0.3631	0.2363
15.33	15.33	57.7	contig00030_-_30S_ribosomal_protein_S7	13	0.3565	0.4999
27.76	27.76	50.8	contig00003_-_ATP_synthase_subunit_alpha	20	0.3532	0.006
2	2	6.4	contig00005_-_Type_4_fimbrial_assembly_protein_PilC	1	0.3532	0.2317
4	4	12	contig00004_-_OmpR,_Response_regulators_consisting_of_a_CheY-like_receiver_domain_and_a_DNA-binding_domain	2	0.3499	0.4762
2	2	28.1	contig00024_-_Predicted_transcriptional_regulator	1	0.3342	0.2207
27.28	27.28	23.7	contig00030_-_elongation_factor_G	16	0.3342	0.1145
2.55	2.55	15.2	contig00010_-_bifunctional_3,4-dihydroxy-2-butanone_4-phosphate_synthase/GTP_cyclohydrolase_II-like_protein	2	0.3311	0.1526
2	2	10.9	contig00003_-_Sulfide:quinone_oxidoreductase,_mitochondrial	1	0.3221	0.2138

2	2	7.2	contig00005_-_Predicted_peroxiredoxins	1	0.3221	0.3778
10.07	10.07	61.2	contig00006_-_rplW,_50S_ribosomal_protein_L23	6	0.3192	0.947
4.82	4.82	14	contig00009_-_N-ethylmaleimide_reductase	3	0.3162	0.1761
25.61	25.61	33.7	contig00004_-_mdoG,_glucan_biosynthesis_protein_G	16	0.3133	0.2827
8.03	8.03	25.9	contig00008_-_SCO1/SenC_protein	4	0.3133	0.0087
3.75	3.75	14.3	contig00003_-_Glutaredoxin,_GrxC_family	2	0.3076	0.7004
2	2	6.1	contig00008_-_Transcriptional_regulator_containing_an_amidase_domain_and_an_AraC-type_DNA-binding_HTH_domain	1	0.3076	0.205
3.14	3.14	8.9	contig00002_-_Lon,_ATP-dependent_Lon_protease,_bacterial_type	3	0.3048	0.0366
6	6	21.4	contig00004_-_Glucosamine-6-phosphate_isomerases/6-phosphogluconolactonase	4	0.3048	0.0724
2	2	7.2	contig00004_-_metG,_methionyl-tRNA_synthetase	1	0.302	0.2032
23.91	23.91	36.8	contig00008_-_Carbohydrate-selective_porin,_OprB_family	19	0.2992	0.1011
2	2	10.1	contig00003_-_membrane_protein_insertase	1	0.2965	0.1996
2	2	8.7	contig00024_-_thiamine_biosynthesis_protein_ThiC	1	0.2965	0.1996
14.63	14.71	38.3	contig00030_-_50S_ribosomal_protein_L10	9	0.2965	0.0301
14.68	14.68	33.1	contig00014_-_ketol-acid_reductoisomerase	11	0.2938	0.1682
2	2	2.2	contig00008_-_Periplasmic_binding_protein	1	0.2911	0.2129
6.06	6.06	23	contig00012_-_YecA_family_protein	5	0.2911	0.0715
22.03	22.03	27.1	contig00003_-_Prc,_Periplasmic_protease	14	0.2884	0.1398
2.44	2.45	4.8	contig00005_-_Cellulose_synthase_operon_protein_C_C-terminus	2	0.2884	0.1703
8	8	23.6	contig00006_-_30S_ribosomal_protein_S14	4	0.2884	0.4474
2	2	11.4	contig00007_-_3'(2'),5'-bisphosphate_nucleotidase,_bacterial	1	0.2858	0.1983
2	2	4	contig00005_-_Probable_3-mercaptopyruvate_sulfurtransferase	1	0.2831	0.1933
2	2	6.7	contig00010_-_NADP-dependent_L-serine/L-allo-threonine_dehydrogenase_YdfG	1	0.2729	0.1881
1.52	1.52	1.7	contig00024_-_bifunctional_heptose_7-phosphate_kinase/heptose_1-phosphate_adenyltransferase	1	0.2704	0.1695
2	2	3.4	contig00002_-_NADPH_dehydrogenase_NamA	1	0.2679	0.3181
4.08	4.08	19.8	contig00002_-_Cupin_domain	2	0.263	0.2909
2.04	2.04	3.7	contig00001_-_phosphoribosylformylglycinamidine_synthase	1	0.2606	0.4463
2.76	2.81	12.5	contig00008_-_Tas,_Predicted_oxidoreductases	2	0.2606	0.1819
11.05	11.05	26.7	contig00006_-_rpsD,_30S_ribosomal_protein_S4	6	0.2559	0.0371
16.62	16.62	29.6	contig00022_-_Glyceraldehyde-3-phosphate_dehydrogenase	11	0.2512	0.005
9.13	9.13	54.1	contig00014_-_LysR,_Transcriptional_regulator	6	0.2466	0.0541
43.59	43.59	39.8	contig00022_-_Carboxysome_shell_peptide_mid-region	42	0.2421	0.4596
12.69	12.69	22.8	contig00003_-_fructose-1,6-bisphosphatase	9	0.2355	0.2032
2.14	2.14	17.7	contig00026_-_Indole-3-glycerol_phosphate_synthase	1	0.2333	0.2996
2	3.59	3.9	contig00001_-_aspartate_aminotransferase	2	0.2312	0.1674
4.01	4.01	20.3	contig00011_-_Putative_signal_peptide_protein	4	0.2291	0.2194
6	6.01	9.3	contig00003_-_Tas,_Predicted_oxidoreductases	3	0.227	0.0695
6.03	6.03	13.8	contig00002_-_Aminoglycoside_phosphotransferase	3	0.2228	0.1224
2.08	2.08	7.4	contig00005_-_UDP-N-acetylmuramoyl-tripeptide--D-alanyl-D-_alanine_ligase	2	0.2228	0.1334

4	4	2.8	contig00026_-_anthranilate_synthase_component_I	2	0.2208	0.9516
2	2	7.6	contig00004_-_fabG,_3-ketoacyl-(acyl-carrier-protein)_reductase	1	0.2188	0.1618
13.6	15.69	22	contig00004_-_DacC,_D-alanyl-D-alanine_carboxypeptidase	9	0.2109	0.0449
4.01	4.01	7.4	contig00001_-_periplasmic_serine_protease,_Do/DeqQ_family	2	0.2051	0.333
4.32	4.33	16.3	contig00005_-_ATP-dependent_chaperone_ClpB	2	0.2051	0.3183
4	4	5.4	contig00005_-_glutamate-1-semialdehyde_aminotransferase	2	0.2051	0.1227
2	2	10.4	contig00005_-_TolQ_protein	1	0.2051	0.3356
2	2	9.8	contig00010_-_pgi,_glucose-6-phosphate_isomerase	1	0.2014	0.245
19.75	21.77	29.9	contig00013_-_eno,_enolase	15	0.2014	0.0454
2	2	11.7	contig00009_-_Vitamin_B12_dependent_methionine_synthase,_activation_domain	1	0.1977	0.1513
4	4	1.9	contig00008_-_cytochrome_c_oxidase,_subunit_I	2	0.1941	0.185
25.88	25.88	39	contig00003_-_glnA,_glutamine_synthetase	21	0.1871	0.093
8	10	15.1	contig00012_-_HDOD_domain	5	0.1871	0.8529
2	2	6.5	contig00022_-_Carboxysome_Shell_Carbonic_Anhydrase	1	0.1871	0.3058
4	4	15.7	contig00025_-_DsrE/DsrF/DrsH-like_family	2	0.1871	0.0922
24.06	24.06	58.4	contig00006_-_rplB,_50S_ribosomal_protein_L2	15	0.1854	0.0041
4.36	4.37	23.1	contig00008_-_Uncharacterized_isochorismatase_family_protein_YwoC	3	0.182	0.0735
9.45	9.45	52.8	contig00022_-_Ribulose_bisphosphate_carboxylase_small_chain	12	0.182	0.025
2.62	2.62	16.5	contig00012_-_ribose-5-phosphate_isomerase_A	2	0.1786	0.2231
10	10	16.3	contig00003_-_Probable_chromosome-partitioning_protein_ParB	6	0.177	0.0136
5.89	6.02	25.6	contig00006_-_DsrE/DsrF-like_family	4	0.1754	0.1143
30.92	30.92	37.6	contig00022_-_Ribulose_bisphosphate_carboxylase_large_chain	26	0.1738	0.0001
2	2	11.6	contig00002_-_SseA,_Rhodanese-related_sulfurtransferase	1	0.169	0.2259
2	2	11.4	contig00013_-_Histidine--tRNA_ligase	1	0.1644	0.1959
2	2	8.6	contig00001_-_isocitrate_dehydrogenase	1	0.1528	0.1312
8.01	8.03	7.5	contig00005_-_Multidrug_resistance_protein_MdtB	4	0.15	0.0687
4	4	9.9	contig00008_-_Hemerythrin_HHE_cation_binding_domain	2	0.15	0.2384
2	2	5.5	contig00013_-_RNA_polymerase_sigma-70_factor,_TIGR02943_family	1	0.1459	0.1286
2	2	12.8	contig00005_-_sucC,_succinyl-CoA_synthetase_subunit_beta	1	0.1445	0.1557
10.02	10.02	21.7	contig00016_-_multidrug_efflux_system_protein_EmrA	7	0.1445	0.0063
6.24	6.24	18.3	contig00003_-_LivK,_ABC-type_branched-chain_amino_acid_transport_systems,_periplasmic_component	4	0.1419	0.2314
10.73	10.73	20.1	contig00002_-_GalU,_UDP-glucose_pyrophosphorylase	5	0.1406	0.005
4.76	4.76	19.7	contig00009_-_gabD1,_succinic_semialdehyde_dehydrogenase	3	0.138	0.12
23.78	24.31	40.4	contig00006_-_DNA-directed_RNA_polymerase_subunit_alpha	17	0.1368	0.0009
2.32	2.33	21.9	contig00008_-_Uncharacterized_ACR,_YkgG_family_COG1556	1	0.1306	0.1217
6.01	6.01	29.1	contig00016_-_DsrE/DsrF-like_family	7	0.1306	0.0278
4.03	4.03	13.8	contig00005_-_Cation_efflux_system_protein_CzcB	2	0.1225	0.0332
5.78	5.78	4.5	contig00003_-_TonB-dependent_siderophore_receptor	3	0.1213	0.0553
36.97	37.02	30.4	contig00016_-_Protein_AsmA	25	0.1148	0.0001

14	14.03	23.5	contig00022_-_Phosphoglycerate_kinase	9	0.1127	0.024
2.01	2.01	11.4	contig00006_-_GuaA,_GMP_synthase_-_Glutamine_amidotransferase_domain	2	0.1107	0.2073
4.91	4.91	32.2	contig00016_-_IbpA,_Molecular_chaperone	4	0.1096	0.1381
4	4	15.1	contig00006_-_Proline-rich_region	2	0.1076	0.201
6	6	41.2	contig00006_-_rplV,_50S_ribosomal_protein_L22	5	0.1038	0.0532
10.09	10.09	32.6	contig00001_-_rpsB,_30S_ribosomal_protein_S2	9	0.1009	0.0368
2	2	5.7	contig00002_-_clpP,_ATP-dependent_Clp_protease_proteolytic_subunit	1	0.0964	0.2692
4	4	7.7	contig00003_-_glyQ,_glycyl-tRNA_synthetase_subunit_alpha	2	0.0964	0.2216
11.45	11.45	21.8	contig00010_-_glyA,_serine_hydroxymethyltransferase	7	0.0964	0.0969
4	4	27.7	contig00007_-_Pilus_assembly_protein,_PilO	2	0.0938	0.1112
4	4.02	12.4	contig00009_-_Prc,_Periplasmic_protease	2	0.0929	0.1217
8.53	8.53	25.4	contig00016_-_SoxAX_cytochrome_complex_subunit_A	5	0.0929	0.0215
2	2	5.3	contig00002_-_Uncharacterized_oxidoreductase_YfjR	1	0.0895	0.2918
8.34	8.34	17.4	contig00004_-_recA,_recombinase_A	6	0.0887	0.0034
2	2	9.9	contig00022_-_S-adenosyl-L-homocysteine_hydrolase	1	0.0887	0.1856
2	2	7.9	contig00007_-_tRNA_threonylcarbamoyladenosine_biosynthesis_protein_RimN	1	0.0832	0.0999
10.02	10.02	46.6	contig00006_-_rpsM,_30S_ribosomal_protein_S13	6	0.0817	0.0079
2	2	7.8	contig00012_-_GIY-YIG_catalytic_domain	1	0.0817	0.2533
47.72	47.72	73.3	contig00004_-_outer_membrane_protein_A	44	0.0802	0.0013
8	8	18	contig00009_-_gcvT,_glycine_cleavage_system_aminomethyltransferase_T	4	0.078	0.012
2	2	9.8	contig00009_-_Predicted_proteasome-type_protease	2	0.0643	0.2635
8.4	8.4	15.5	contig00008_-_signal_peptide_peptidase_SppA,_67K_type	4	0.0637	0.0194
13.44	14.82	37.2	contig00001_-_RND_family_efflux_transporter,_MFP_subunit	11	0.0631	0.0394
2.01	2.03	11.3	contig00006_-_heat_shock_protein_90	1	0.0575	0.1689
36.27	36.27	41.2	contig00022_-_fructose-1,6-bisphosphate_aldolase	29	0.056	0.0003
2	2	9	contig00001_-_tmk,_thymidylate_kinase	1	0.0555	0.086
2	2	3.5	contig00006_-_secY,_preprotein_translocase_subunit_SecY	1	0.0555	0.1405
6.95	6.95	39.1	contig00006_-_rpsC,_30S_ribosomal_protein_S3	5	0.0545	0.0007
4	4	33.7	contig00006_-_rpsP,_30S_ribosomal_protein_S16	2	0.0545	0.2534
2.38	2.38	13.6	contig00015_-_Domain_of_unknown_function_(DUF4340)	1	0.0525	0.1044
12.06	12.06	21.1	contig00007_-_Phosphate-binding_protein_PstS_1	16	0.0506	0.0011
14.88	14.88	22.1	contig00002_-_periplasmic_folding_chaperone	8	0.0497	0.0004
2	2	6.1	contig00010_-_phosphoenolpyruvate_carboxylase	1	0.0497	0.1279
11.75	11.75	14.9	contig00012_-_hypothetical_protein	6	0.0497	0.0176
6.01	6.01	22.9	contig00010_-_The_CBS_domain,_a_small_domain_originally_identified_in_cystathionine_beta-synthase	4	0.0492	0.0505
21.96	21.96	73.3	contig00066_-_Major_carboxysome_shell_protein_1A	38	0.0453	0.0276
4	4	6.1	contig00014_-_HDOD_domain	2	0.0449	0.1198
11.64	11.64	47.3	contig00006_-_rplE,_50S_ribosomal_protein_L5	7	0.0413	0.0585
14	14	39.3	contig00001_-_Translation_initiation_factor_IF-3	8	0.0391	0.0002

16.59	16.59	20	contig00014_-_multifunctional_aminopeptidase_A	12	0.0363	0.0018
3.26	3.26	8.4	contig00001_-_Phosphoketolase	3	0.0331	0.1334
10.49	10.49	12.3	contig00001_-_MrcA,_Membrane_carboxypeptidase/penicillin-binding_protein	8	0.0322	0.0067
4.01	4.01	20.9	contig00009_-_Membrane_Fusion_Protein_cluster_2	3	0.0302	0.0608
12	12	37.2	contig00005_-_Putative_peptidoglycan_binding_domain	8	0.0231	0.0051
2	2	17.7	contig00010_-_ribH,_6,7-dimethyl-8-ribityllumazine_synthase	1	0.0223	0.1107
3.1	3.1	16.3	contig00001_-_PepB,_Leucyl_aminopeptidase	2	0.0194	0.0689
16.77	17.73	20.7	contig00017_-_UshA,_5'-nucleotidase/2',3'-cyclic_phosphodiesterase_and_related_esterases	14	0.0149	0
10.34	10.34	21.9	contig00001_-_6-phosphogluconate_dehydrogenase	6	0.0136	0.0036
2.04	2.04	7.2	contig00001_-_fructose-bisphosphate_aldolase	2	0.0112	0.1077
2	2.01	5.7	contig00011_-_Protein_CapI	1	0.0112	0.0571
2	2	32.8	contig00005_-_3-deoxy-D-manno-octulosonate_8-phosphate_phosphatase_KdsC	1	0.0111	0.0657
1.74	2	2	contig00011_-_adenylosuccinate_synthetase	1	0.0111	0.0642
3.48	3.48	5	contig00022_-_CheA,_Chemotaxis_protein_histidine_kinase_and_related_kinases	2	0.0111	0.0561
2	2	4.7	contig00001_-_5'-methylthioadenosine_phosphorylase	1		
2	2	5.4	contig00001_-_ompR,_osmolarity_response_regulator	1		
4	4	11.6	contig00003_-_PF03625_domain_protein	2		
2	2	13.6	contig00004_-_Glucokinase	1		
2	2	5.4	contig00005_-_ddl,_D-alanine--D-alanine_ligase	1		
2	2	4.5	contig00005_-_UDP-N-acetylglucosamine_1-carboxyvinyltransferase	1		
2.01	2.01	4.1	contig00006_-_leuS,_leucyl-tRNA_synthetase	1		
2	2.02	9.6	contig00008_-_Cytochrome_C1_family	1		
8	10	23.9	contig00008_-_GlnK,_Nitrogen_regulatory_protein_PII	5		
3.11	3.11	6.5	contig00008_-_NirB,_NAD(P)H-nitrite_reductase	3		
2	2	14	contig00010_-_YciI-like_protein	1		
2	2	3.8	contig00011_-_amidophosphoribosyltransferase	1		
2	2	2	contig00011_-_beta_alanine--pyruvate_transaminase	1		
2	2.09	4.7	contig00011_-_methylmalonic_acid_semialdehyde_dehydrogenase	2		
2	2	5	contig00012_-_upp,_uracil_phosphoribosyltransferase	1		
4	4	3.8	contig00013_-_hscA,_chaperone_protein_HscA	3		
4	4	18.4	contig00015_-_acetyl-CoA_carboxylase_biotin_carboxyl_carrier_protein_subunit	4		
2	2.02	8.5	contig00015_-_ATP-dependent_RNA_helicase_RhlB	2		
2	2	13.6	contig00017_-_monothiol_glutaredoxin,_Grx4_family	1		
2	2	2.9	contig00024_-_rfaD,_ADP-L-glycero-D-mannoheptose-6-epimerase	1		
2	2	4.8	contig00027_-_TonB-dependent_siderophore_receptor	1		

Supplemental File 3 COG classification with the highest number of differentially expressed *Ac. prosperus*^{T} proteins identified by iTRAQ analysis

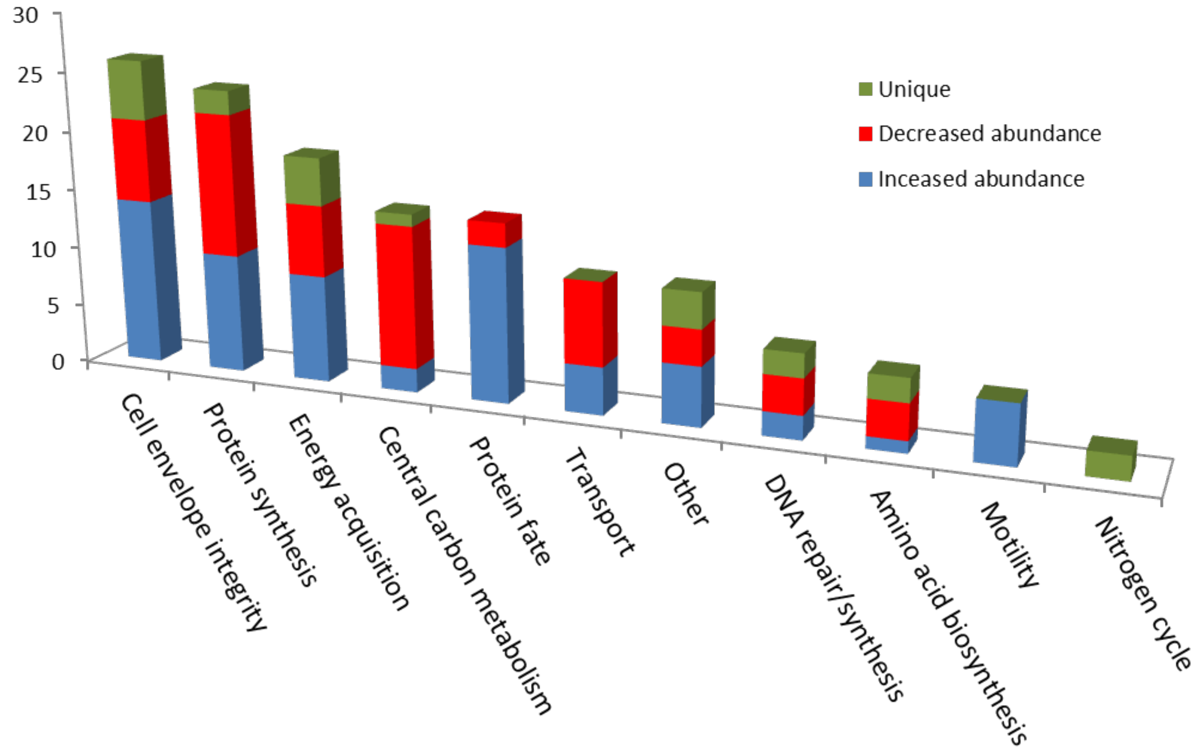

Supplemental File 4 Two dimensional polyacrylamide gels of *At. ferrooxidans*^{T} total soluble protein (400 µg/gel) stained with blue silver colloidal coomassie blue when grown in the presence of low (0 g/L NaCl; left image) or high (8 g/L NaCl; right image).

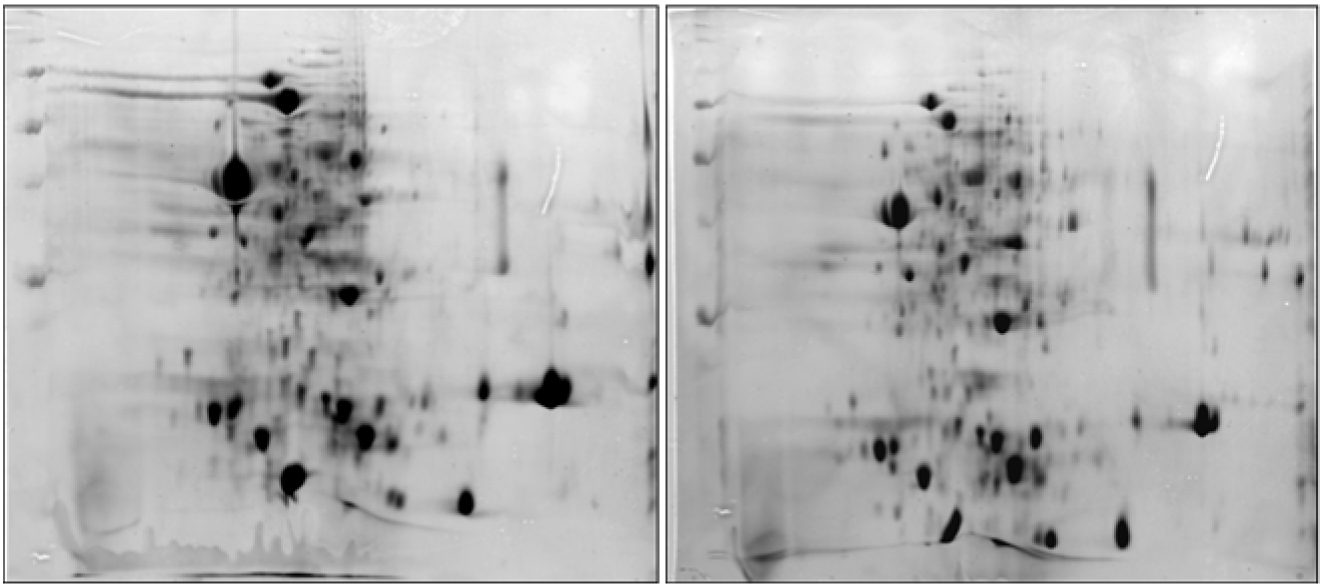

Supplemental File 5 *At. ferrooxidans*^{T} proteins with statistically supported altered abundance when grown in high or low NaCl concentration.

UniProt Number[a]	Protein	Experimental Mw/pI[b]	Theoretical Mw/pI[c]	Anova (p)[d]	Fold Increase[e]
**Proteins with increased abundance in high NaCl conditions**					
B7J7R8	Pyridine nucleotide-disulfide oxidoreductase	46/6.81	42.454/6.19	1.2E-04	4.2
B7JBC9	Glyceraldehyde-3-phosphate dehydrogenase, type I	37/7.16	36.75/6.45	0.004	4
B7J9P4	Ribosome recycling factor	22/6.81	20.74/6.22	0.009	4
B7JBN3	Inosine-5'-monophosphate dehydrogenase	55/7.02	51.40/6.37	0.049	3.5
B7J432	5-methyltetrahydropteroyltriglutamate--homocysteine methyltransferase	98/6.79	88.27/5.86	0.025	3.4
B7JA08	Survival protein SurA	54/6.61	52.43/6.40	0.049	3.4
B7J3E6	Sulphur/pyrite/thiosulphate/sulphide-induced protein	19/6.11	24.05/9.36	0.001	2.9
B7J541	PpiC-type peptidyl-prolyl *cis-trans* isomerase	30/9.47	28.005/9.24	0.026	2.8
B7J3E4	Periplasmic solute binding protein	36/8.76	36.707/9.10	0.038	2.8
B7J3E4	Periplasmic solute binding protein	35/9.28	36.707/9.10	0.04	2.6
B7J541	PpiC-type peptidyl-prolyl *cis-trans* isomerase	31/8.89	28.005/9.24	3.9E-04	2.5
B7J4U6	Heat shock protein Hsp20	18/6.61	16.92/6.20	0.026	2.5
B7J3E4	Periplasmic solute binding protein	34/6.74	36.70/9.10	0.007	2.3
B7J4J7	Gamma-glutamyl phosphate reductase	45/7.06	45.68/6.35	0.038	2.3
B7J6R4	Enolase	46/5.04	45.87/4.68	0.026	2.2
B7J6Y5	Putative uncharacterized protein	16/5	13.86/5.01	0.024	2.1
B7JBC9	Glyceraldehyde-3-phosphate dehydrogenase, type I	37/7	36.75/6.45	0.05	2
B7J942	Serine protease, DO/DeqQ family	59/7.4	53.42/7.3	0.123	2
B5EQ90*	Aminomethyltransferase	41/6.7	39.55/6.0	0.129	2.3
**Proteins with increased abundance in low NaCl conditions**					
B7J3E4	Periplasmic solute binding protein	34/5.0	36.70/9.1	3.71E-04	3.1
P0C918	Rusticyanin	19/7.95	19.90/8.80	0.006	2.5
B7J7L4	Glycine cleavage system H protein	31/4.8	15.19/4.70	0.007	1.9
B7J8H1	Major outer membrane protein 40	39/5.02	42.22/4.93	0.005	1.8
B7J913	50S ribosomal protein L9	18/7.25	15.99/6.52	0.022	1.8

[a]Uniprot accession number, refers to the identified protein within this database.
[b]Experimental Mw/pI was determined by protein standard and pH strip (linear gradient) annotations made within Progenesis Same Spots (Non Linear Dynamic, USA) program.
[c]Theoretical MR/PI was determined from the Uniprot database.
[d]Anova P value is a measure provided by Progenesis Same Spot (Non Linear Dynamic, USA) program which accounts for the multiple gels spot intensities for individual protein spots. P values below 0.05 are statistically significant.
[e]Average fold up-regulation between the high and low salt proteomes.

Supplemental File 6 Genetic contexts of the rusticyanin genes (*rus*) Forms I and II from *Ac. prosperus*^{T} compared to *rus* from *At. ferrooxidans*^{T}. The information for *At. ferrooxidans* is taken from Valdés et al (2008) *Acidithiobacillus ferrooxidans* Metabolism: From Genome Sequence to Industrial Applications. BMC Genomics, 9:597.

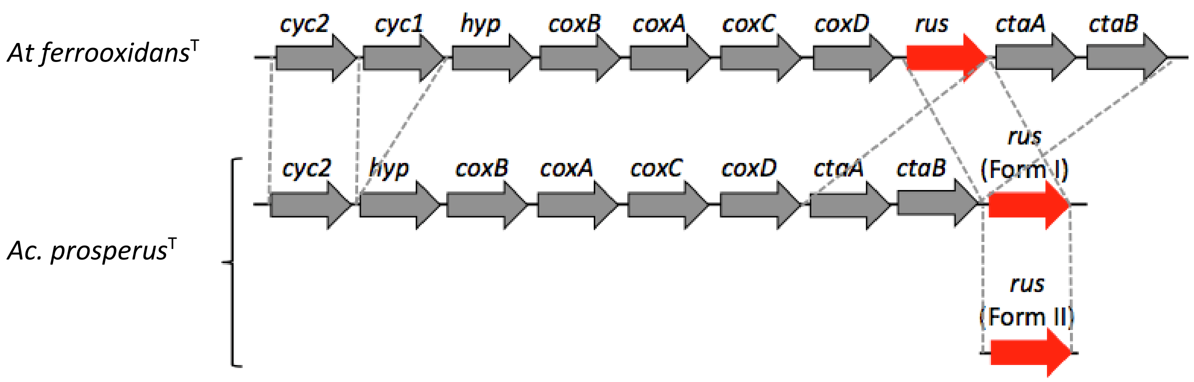

Supplement: Supplementary file 1 [file DataSheet1.PDF]
